# Supplementary material for: A Comparative Analysis of RNAi Trigger Uptake and Distribution in Mosquito Vectors of Disease
Source: Insects. 2023 Jun 15;14(6):556. doi: 10.3390/insects14060556 (PMC10299510; doi:10.3390/insects14060556)

Supplementary File – Supplementary Tables & Figures for:

A Comparative Analysis of RNAi Trigger Uptake and Distribution in Mosquito Vectors of Disease

Paul M. Airs [1], Katherine E. Kudrna [1], Bailey Lubinski [1], Yashdeep Phanse [1] and Lyric C. Bartholomay [1,2,*]

[1] Department of Pathobiological Sciences, University of Wisconsin—Madison, Madison, WI, 53706, USA
[2] Midwest Center of Excellence for Vector-Borne Diseases, University of Wisconsin—Madison, Madison, WI, USA
* Correspondence: lyric.bartholomay@wisc.edu

Contents

Table S1 – Primers used in study 2

Supplementary Figures 2

Bio-distribution of fluorescently labelled iLacZ in mosquitoes 2

Exposure by Intrathoracic Injection 3

Figure S1 – Adult female *Ae. aegypti* - intrathoracic injection 3

Figure S2 – Adult female *An. gambiae* - intrathoracic injection 10

Figure S3 – Adult female *Cx. Pipiens* - intrathoracic injection 17

Figure S4 – Adult female *Ae. aegypti* 25-48 hours post blood/meal - intrathoracic injection 25

Figure S5 – Adult female *An. gambiae* 25-48 hours post blood/meal - intrathoracic injection 29

Figure S6 – Adult female *Cx. pipiens* 25-48 hours post blood/meal - intrathoracic injection 33

Figure S7 – 4th instar *Ae. Aegypti* - intrathoracic injection 37

Figure S8 – 4th instar *An. gambiae* – intrathoracic injection 39

Figure S9 – 4th instar *Cx. pipiens* – intrathoracic injection 41

Adult exposure by Topical Application 44

Figure S10 – Adult female *Ae. aegypti* – topical exposure 44

Figure S11 – Adult female *An. gambiae* – topical exposure 47

Figure S12 – Adult female *Cx. pipiens* – topical exposure 49
Adult exposure *Per os* 53
Figure S13 – Adult female *Ae. aegypti – per os* 53
Figure S14 – Adult female *Cx. pipiens – per os* 58
Larval exposure by Soaking 63
Figure S15 – 1st instar *Ae. aegypti* - soaking 63
Figure S16 – Stability of iLacZ in sucrose meal following exposure to *Ae. aegypti* in insectary conditions. 65
Figure S17 – Confocal Z/stack of *Ae. aegypti* ovary 24 hours post peritoneal exposure of fluorescent/iLacZ. 66
Figure S18 – Northern blot time-course of C6/36 cells following iLacZ exposure. 67

Table S1 – Primers used in study

Various LacZ PCR products were produced as template for production of dsRNA and siRNA. For RNAi trigger synthesis and northern blot probe synthesis the T7 sequence (TAATACGACTCACTATAGGG) was added to the 5' of both the forward and reverse primers.

Product	Primer Name	Direction	Sequence	Product Size
LacZ 377†	LacZ 377 F	Forward	CTTTTGCTGGCCTTTTGCTC	377
	LacZ 377 R	Reverse	CGTAATCATGGTCATAGCTGTTTCC	
LacZ 200	LacZ 200 R	Reverse	GCATTAATGAATCGGCCAAC	199
LacZ 400‡	LacZ 400 R	Reverse	GTCACCTAAATAGCTTGGCGTAA	395
LacZ 600	LacZ 600 R	Reverse	GTTTTCCCAGTCACGACGTT	602

† = PCR products used for northern blot probe generation
‡ = PCR product used for fluorescent labeling experiments.
* The same F primer was used for all iLacZ products

Supplementary Figures

Bio-distribution of fluorescently labelled iLacZ in mosquitoes

Supplementary figures 1 – 15 include representative images taken of live mosquito tissues dissected in PBS and imaged following exposure to fluorescent Cy/3 labelled iLacZ (377 bp). Tables are formatted showing bright field, iLacZ (Cy3 fluorescence) and merged image panels. Image panel descriptions are formatted as

'Region / Tissue or cell types / Hours Post Exposure (HPE). * Tissues or cells of interest containing iLacZ signal'. Figures are ordered into subsections by exposure route (in bold) with each figure representing a single species at a single life/stage. Due to the number of conditions images have not been included for every tissue for every time/point in every experiment but represent an image collection reference for Table 1.

Exposure by Intrathoracic Injection

Figure S1 – Adult female *Ae. aegypti* - intrathoracic injection

Description	Bright Field	RNAi trigger	Merge
Head / injection site / 24 HPE	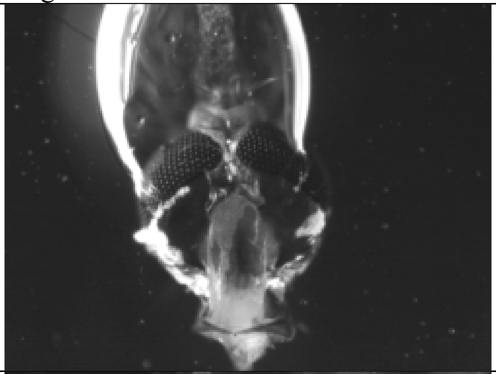	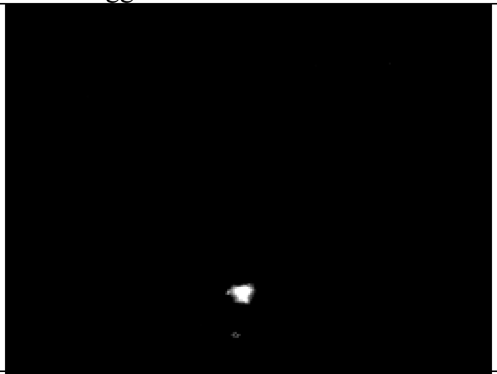	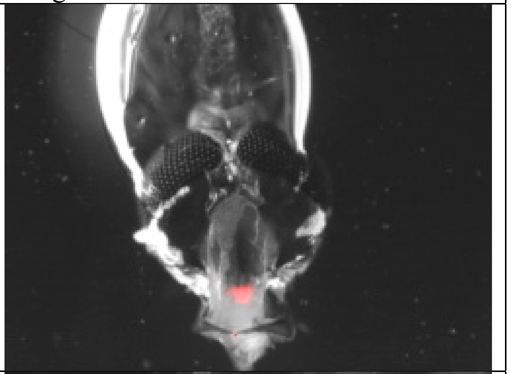
Thorax / cuticle, muscle* / 24 HPE<br><br>* hemocytes with iLacZ	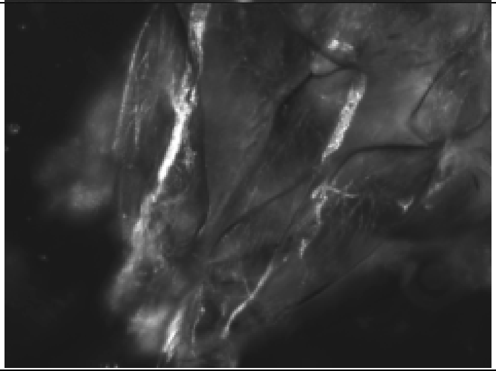	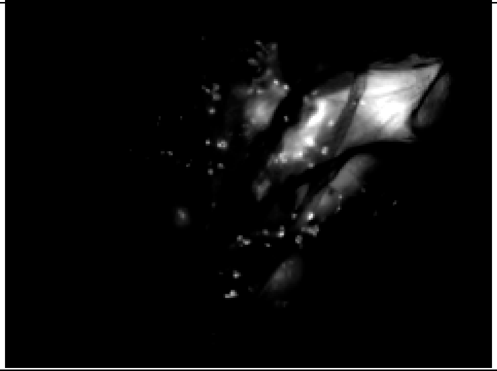	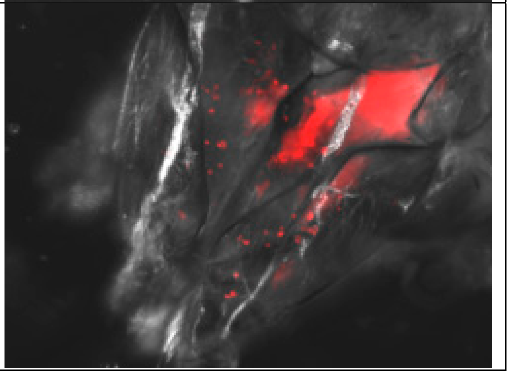

Thorax / Cuticle, wing, muscle, fat body* / 72 HPE<br>* Pericardial cells with iLacZ, auto-fluorescence in cuticle	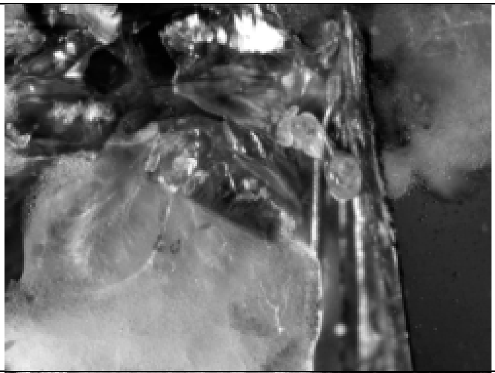	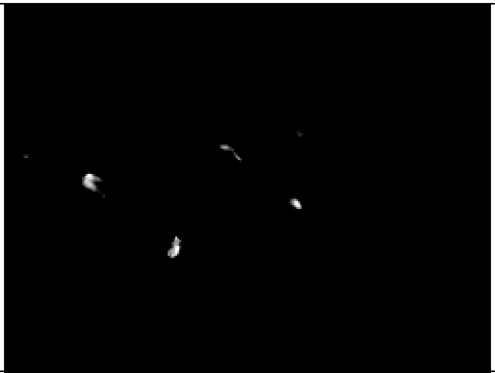	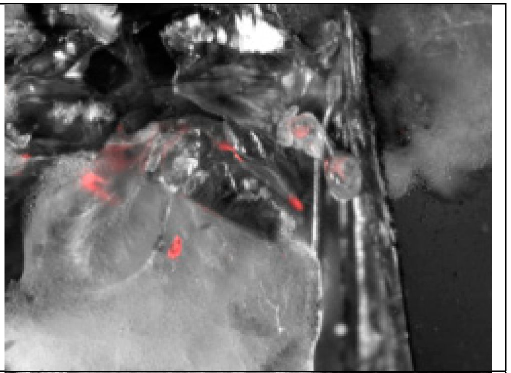
Abdomen / cuticle, fat body * / 72 HPE<br><br>* pericardial cells with iLacZ	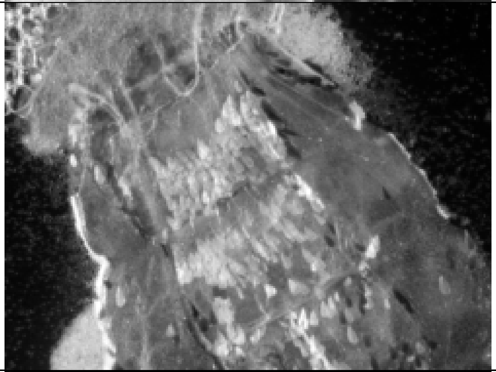	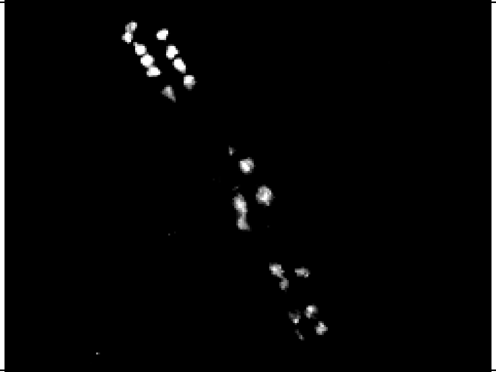	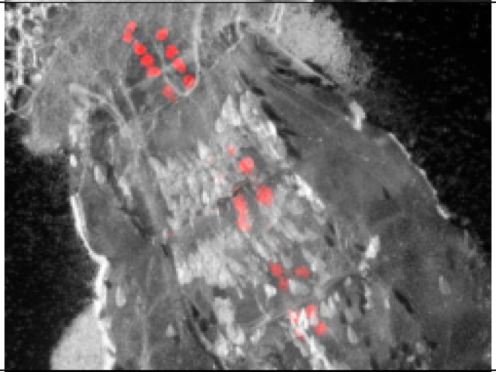
Abdomen / Cuticle, fat body * / 24 HPE<br><br>* pericardial cells, nerve cord ganglia with iLacZ	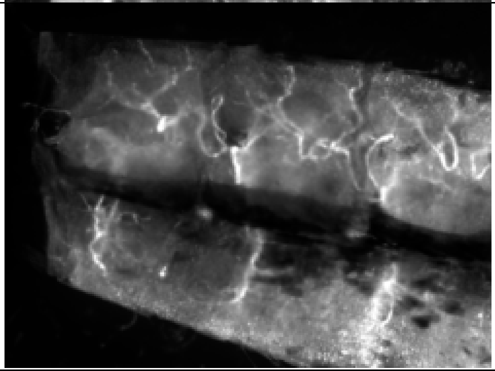	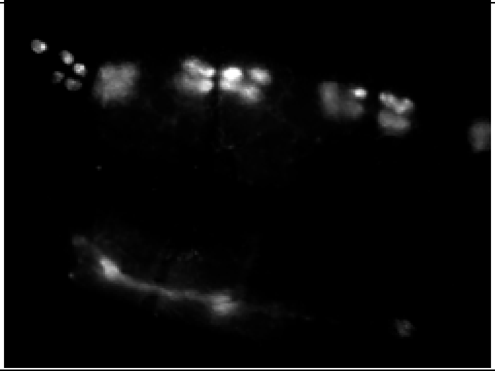	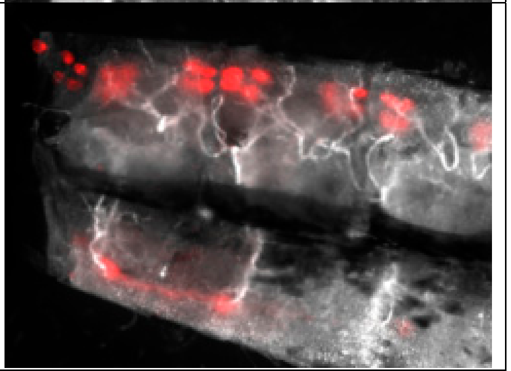

Abdomen / Fat body & hemocytes* / 120 HPE<br><br>* hemocytes with iLacZ	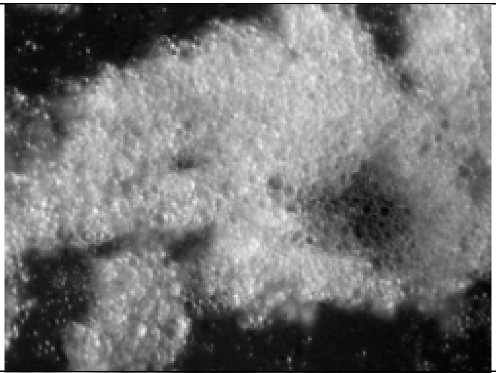	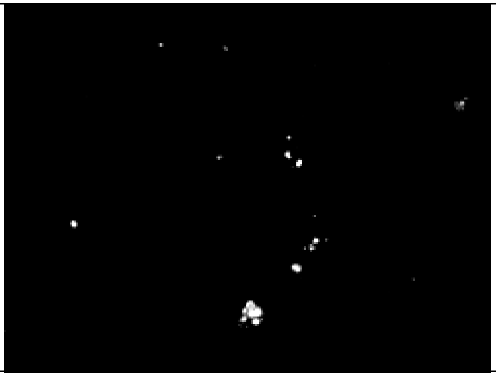	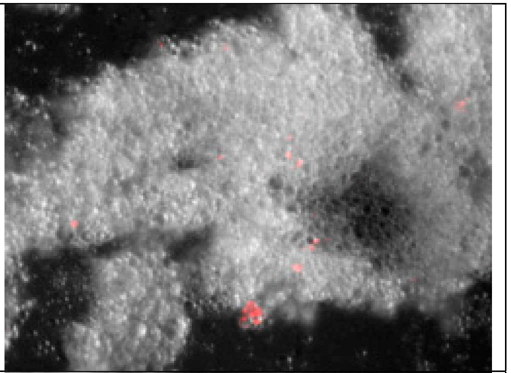
Abdomen / Malpighian tubule and tracheoles* / 72 HPE<br><br>* hemocytes with iLacZ	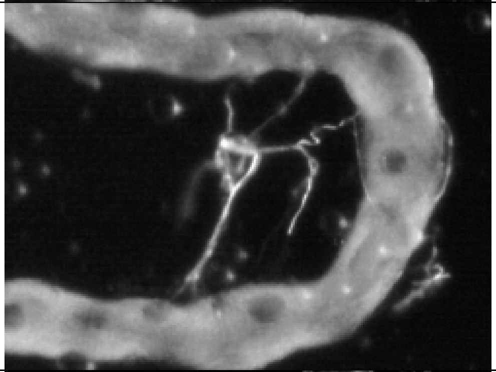	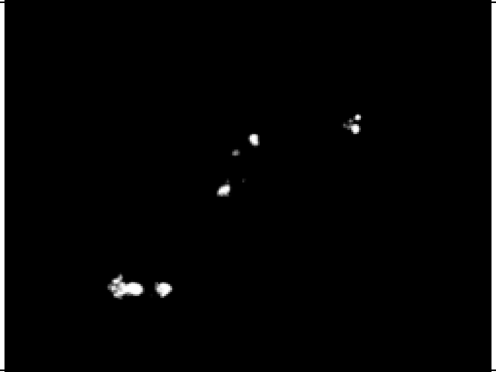	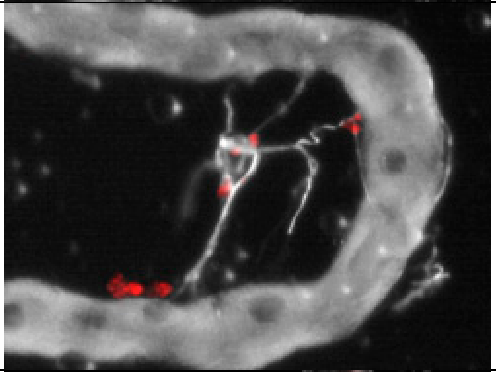
Leg / Cuticle & hemocytes* / 72 HPE<br><br>* hemocytes with iLacZ	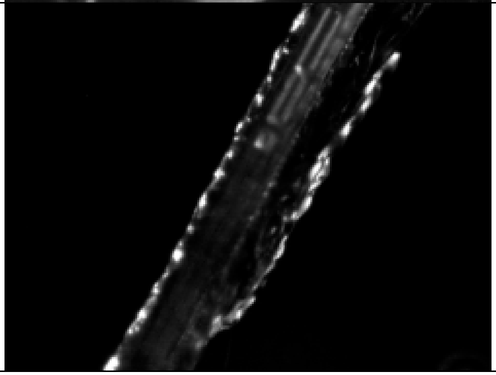	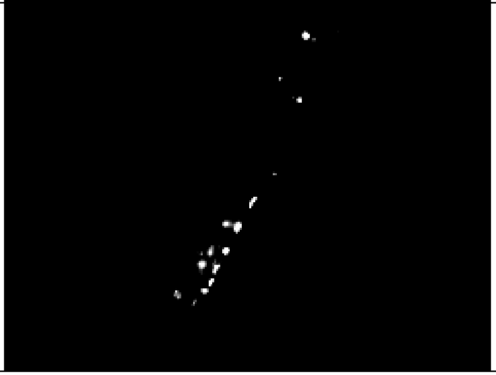	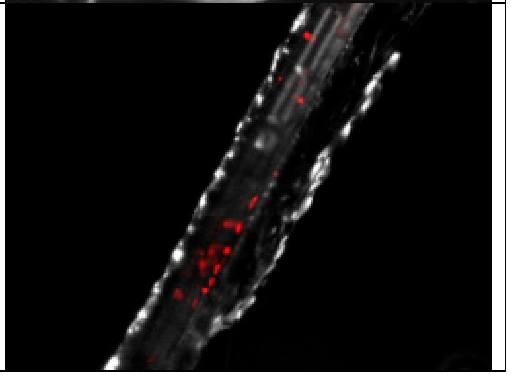

Abdomen / Anterior alimentary tract / 72 HPE	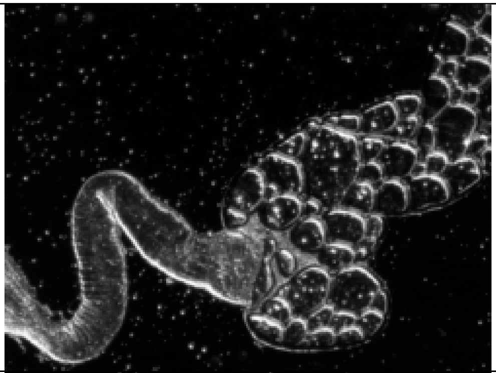	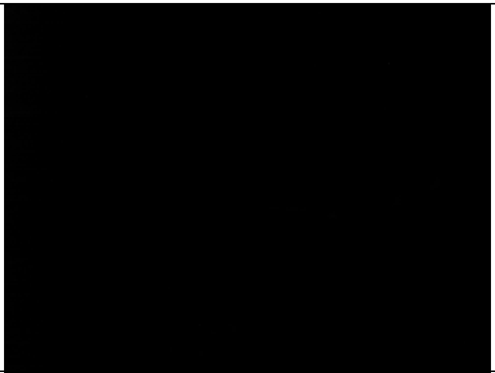	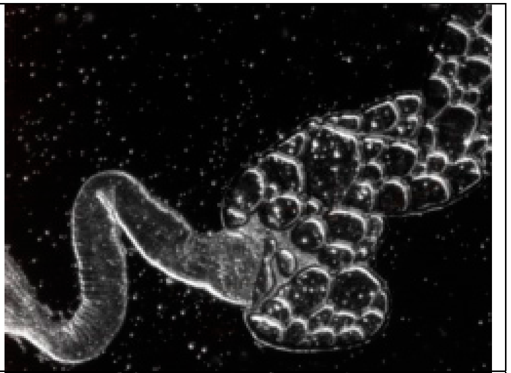
Abdomen / Central alimentary tract / 120 HPE	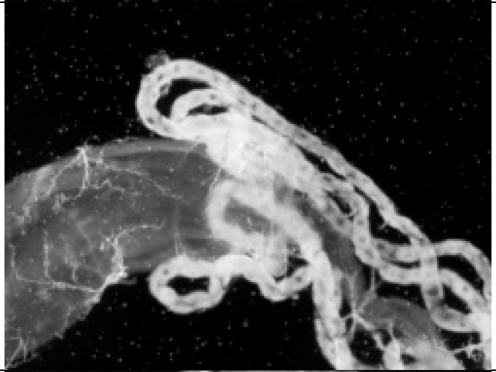	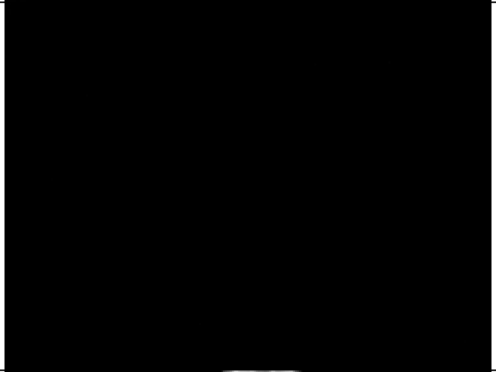	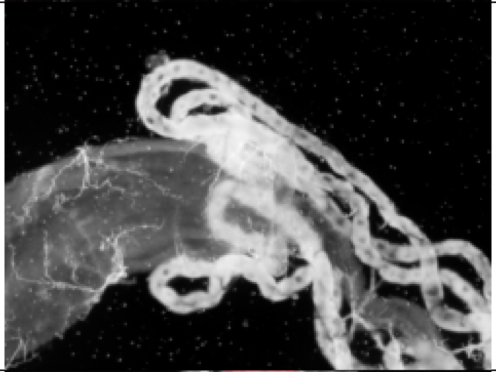
Abdomen / Posterior alimentary tract * / 72 HPE<br><br>* hemocytes and gut lumen with iLacZ	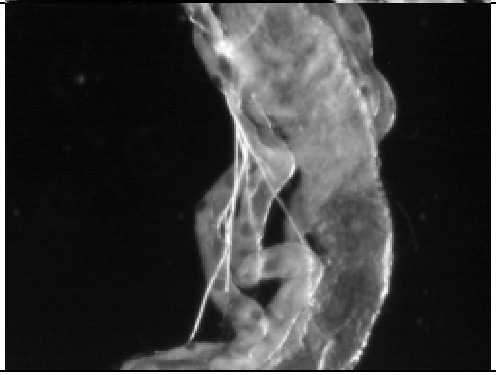	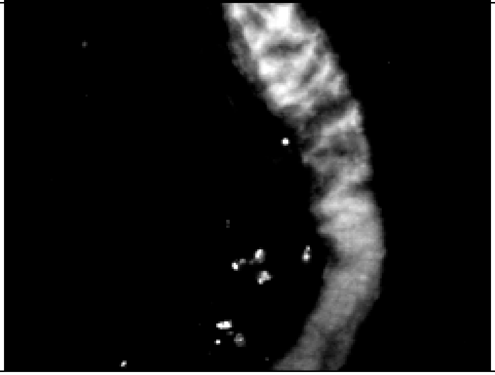	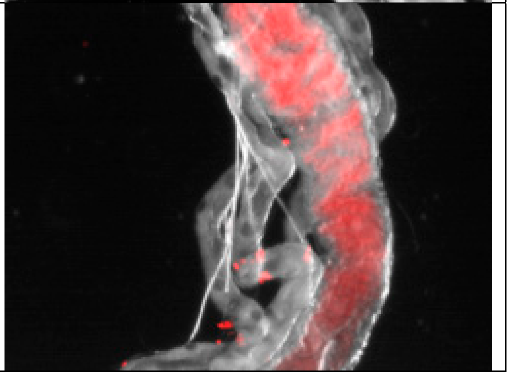

Abdomen / Posterior alimentary tract * / 72 HPE<br><br>* hemocytes and rectum lumen with iLacZ	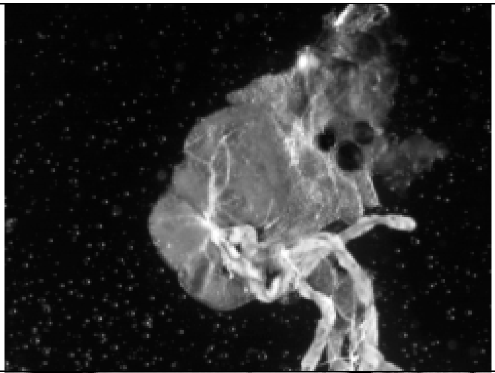	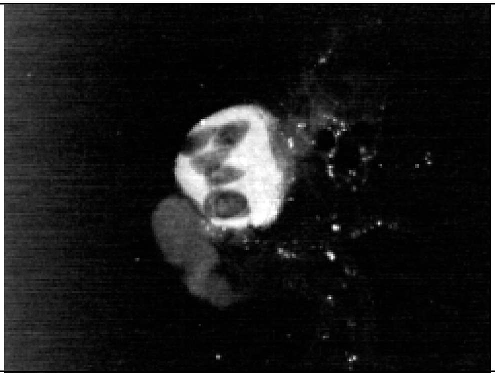	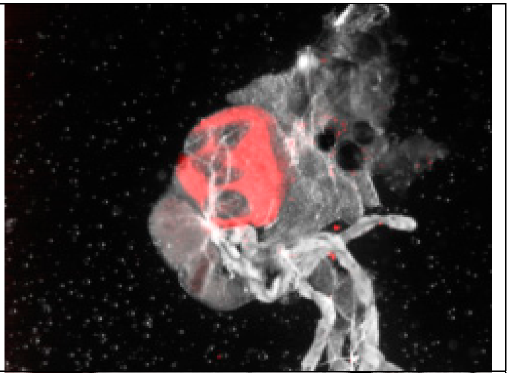
Abdomen / Ovary */ 24 HPE<br><br>* hemocytes with iLacZ	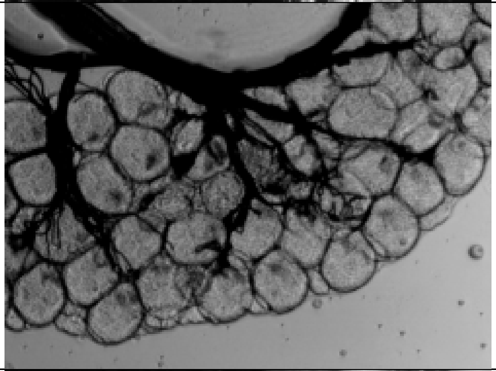	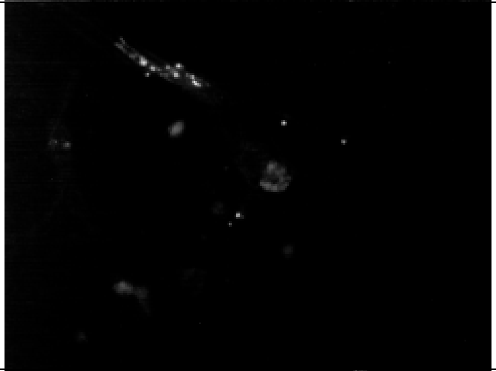	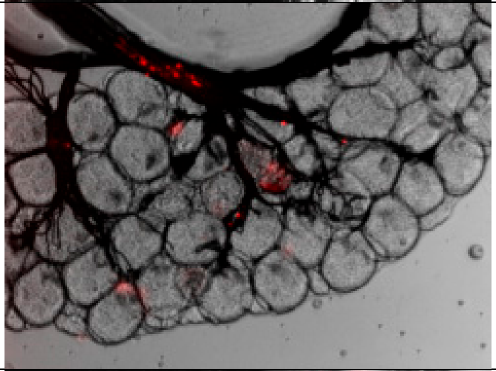
Abdomen Ovary */ 120 HPE<br><br>* Oocytes with iLacZ	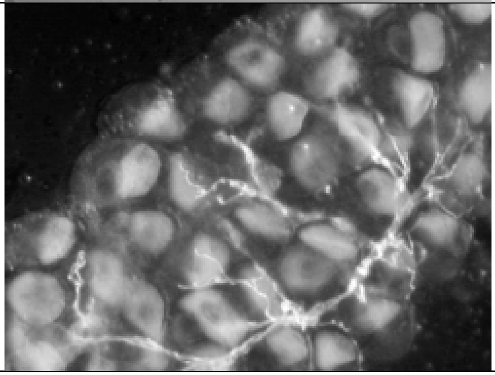	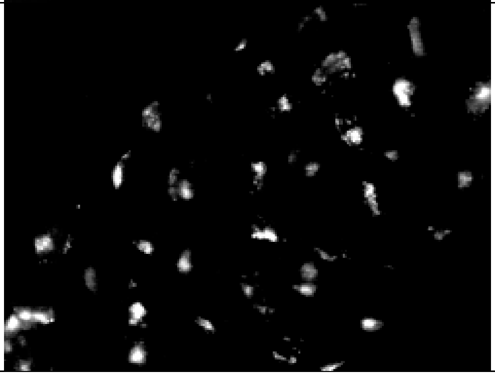	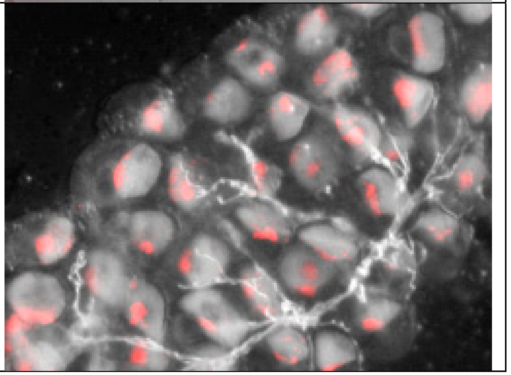

Un-injected negative controls for autofluorescence<br><br>Head with autofluorescence in cuticle	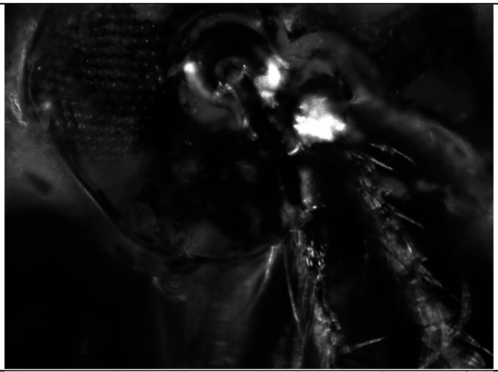	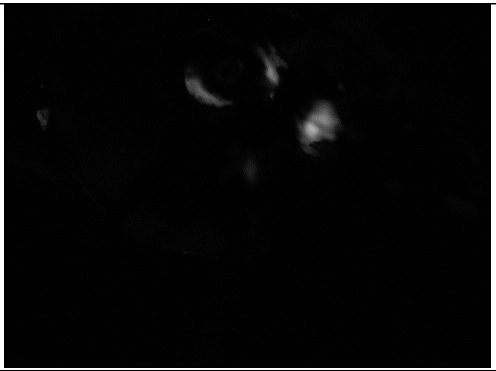	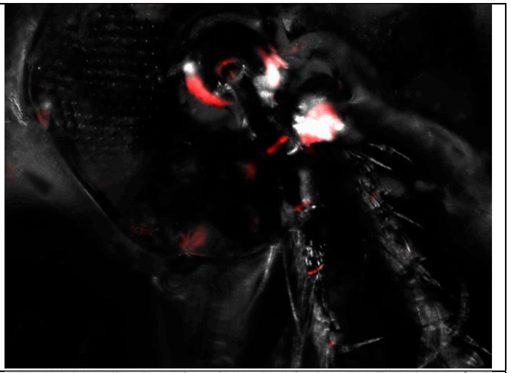
Un-injected negative controls for autofluorescence<br><br>Abdomen with autofluorescence in soft cuticle	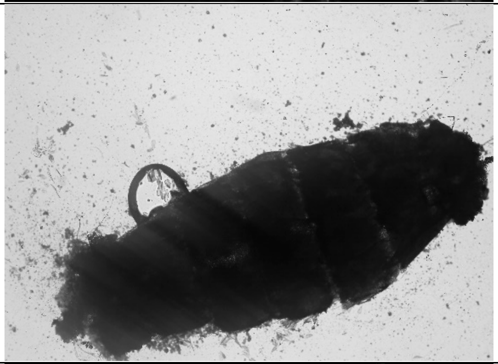	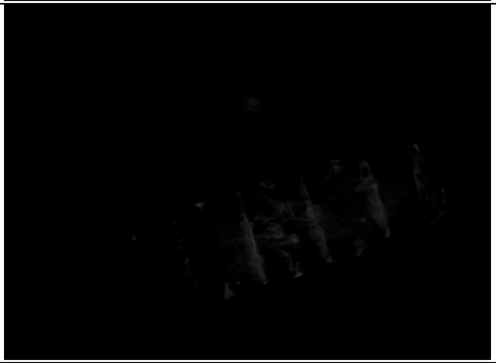	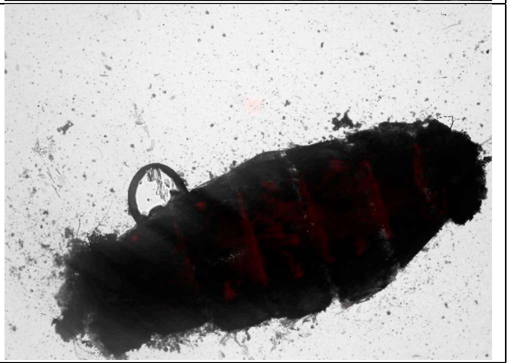
Un-injected negative controls for autofluorescence<br><br>Malphigian tubules	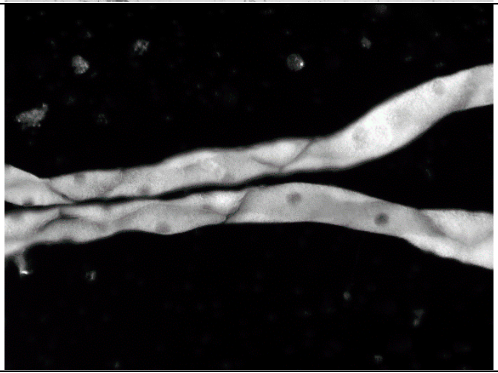	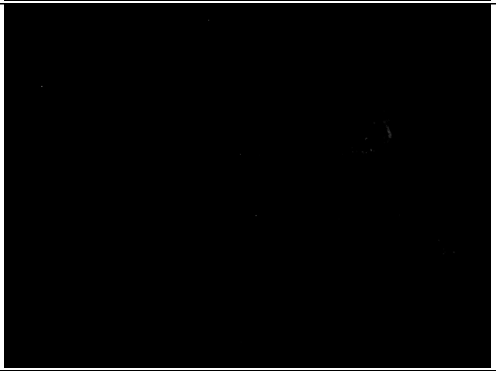	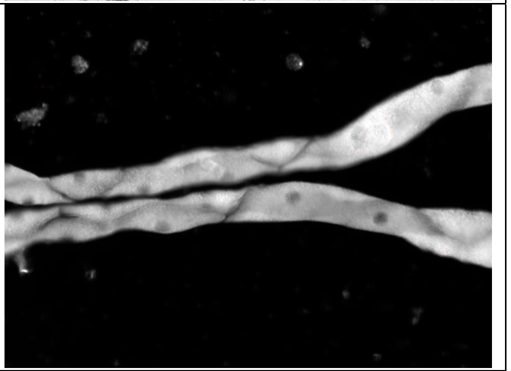

Un-injected negative controls for autofluorescence<br><br>Midgut	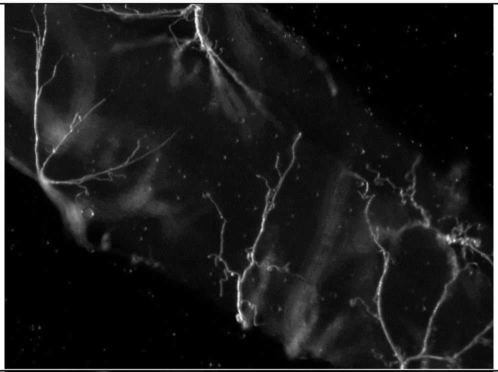	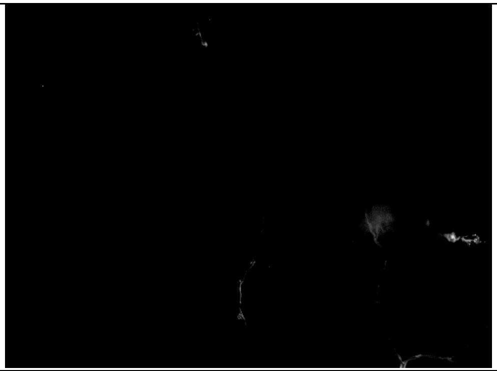	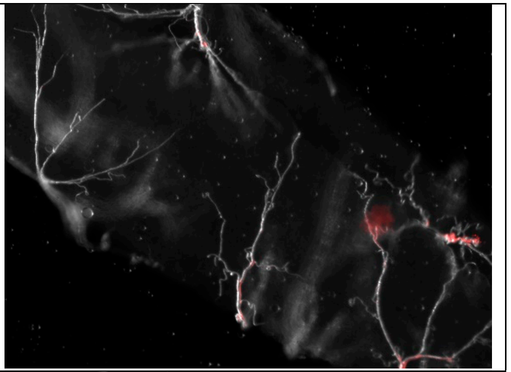
Un-injected negative controls for autofluorescence<br><br>Ovaries with tracheole autofluorescence	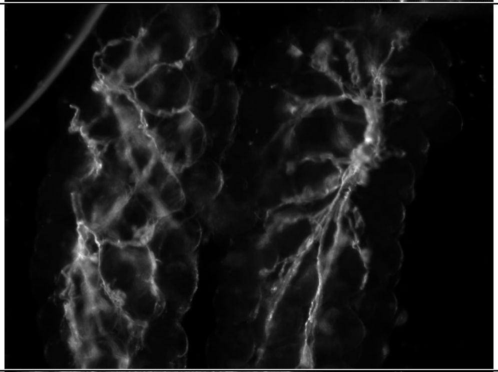	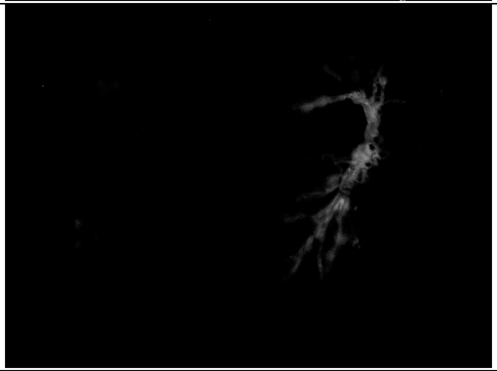	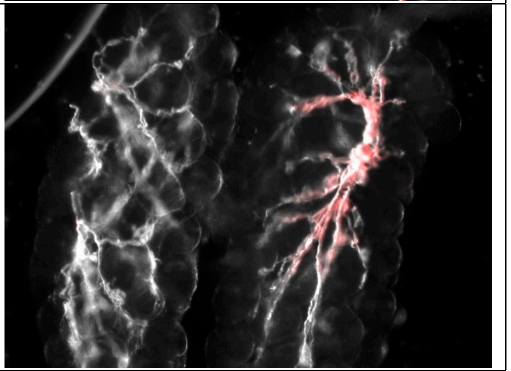
Un-injected negative controls for autofluorescence<br><br>Fat body & hemocytes with autofluorescence (possibly tracheole)	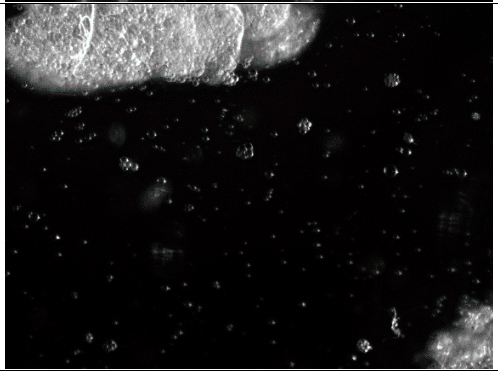	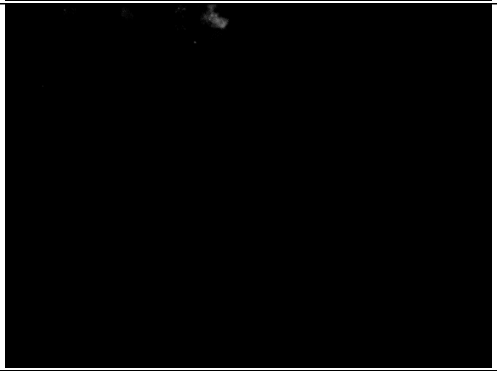	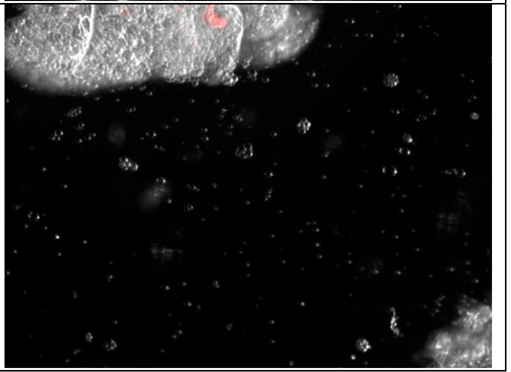

Un-injected negative controls for autofluorescence<br><br>Ovaries (post blood-feeding)	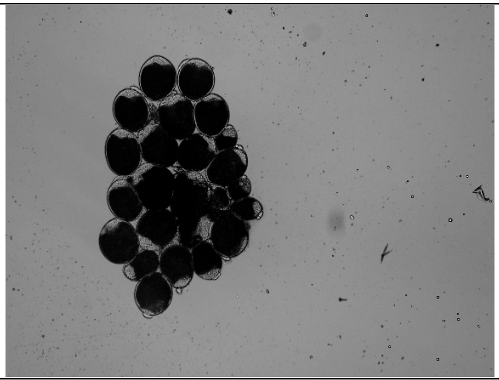	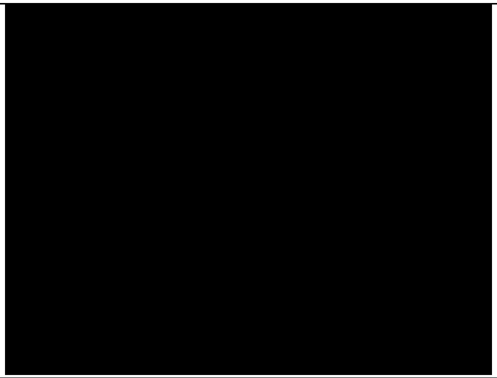	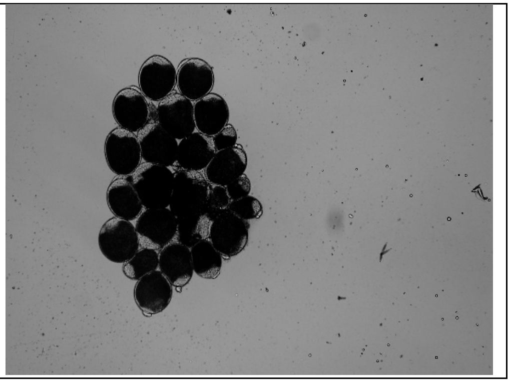

Figure S2 – Adult female *An. gambiae* - intrathoracic injection

Tissue	Bright Field	RNAi trigger	Merge
Head / injection site * / 24 HPE<br><br>* Injection site with iLacZ	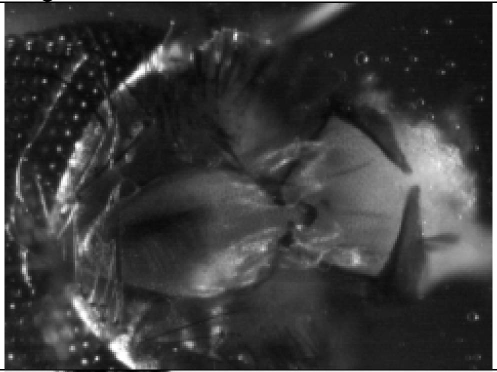	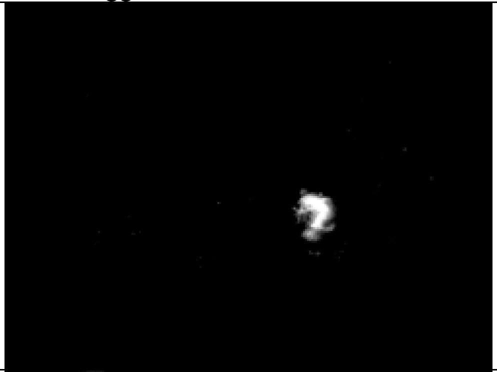	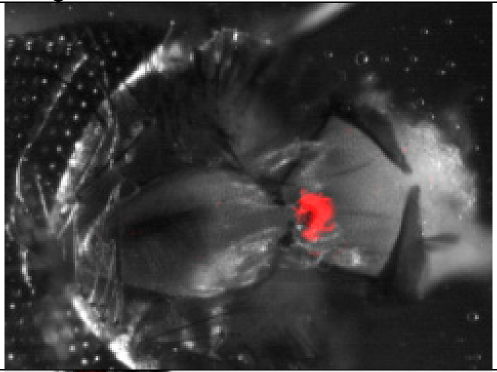
Head / Palps* / 72 HPE<br><br>* hemocytes with iLacZ and Extracellular signal	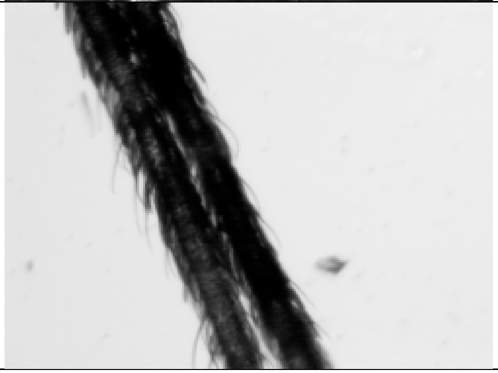	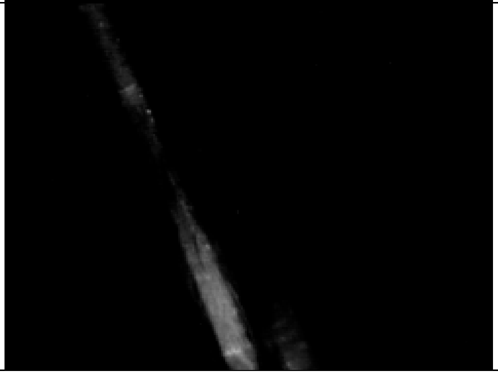	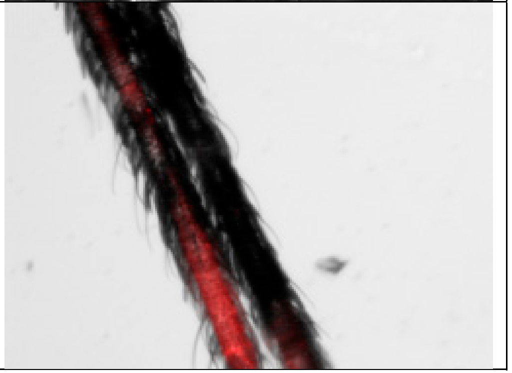

Thorax / Cuticle, muscle / 24 HPE<br><br>* auto-fluorescence in cuticle and hemocytes with iLacZ	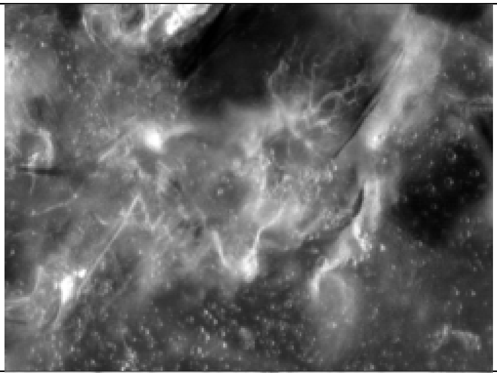	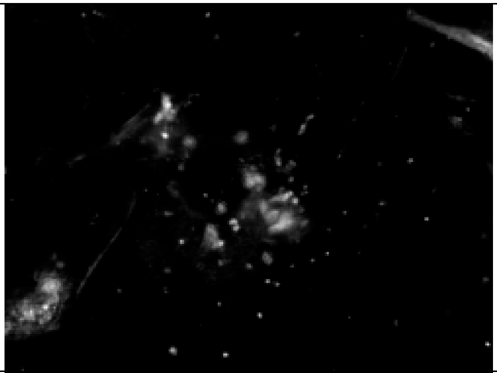	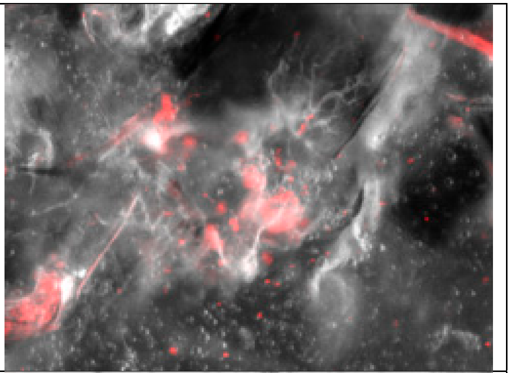
Thorax / Muscle and cuticle / 24 HPE<br><br>* Auto-fluorescence in cuticle	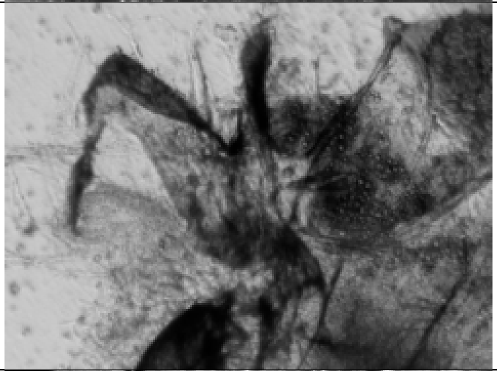	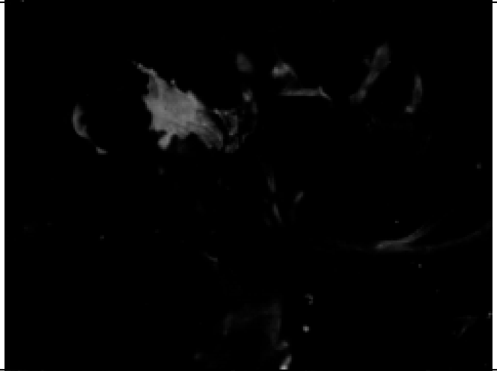	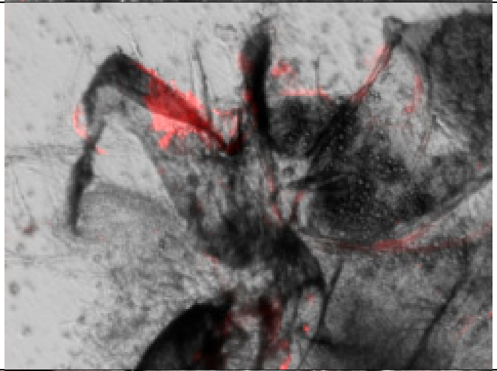
Abdomen / Cuticle * / 72 HPE<br><br>* Pericardial cells with iLacZ	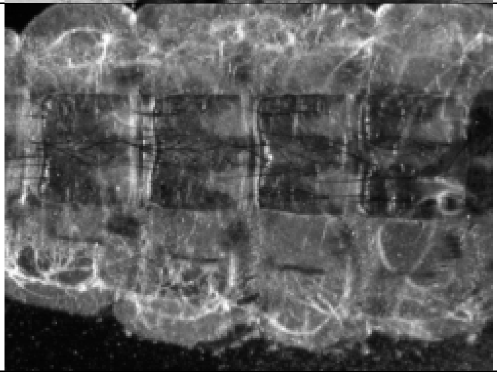	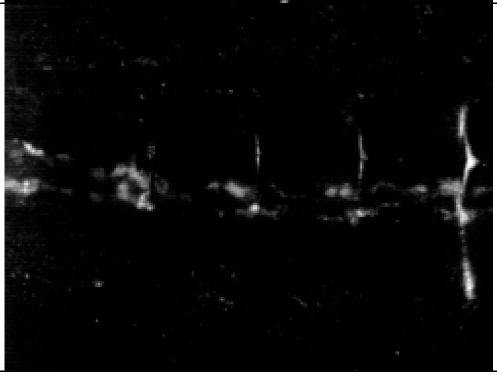	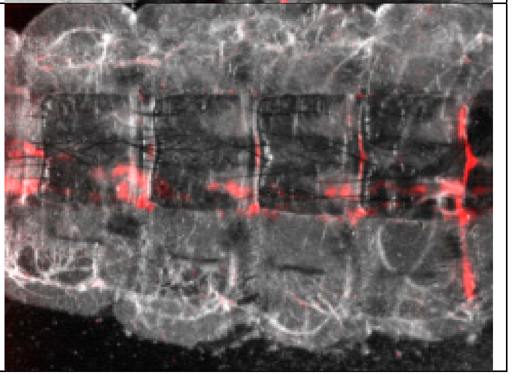

Abdomen / Cuticle, fat body* / 72 HPE<br><br>* pericardial cells with iLacZ	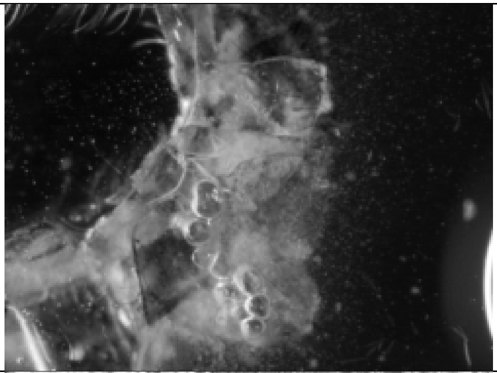	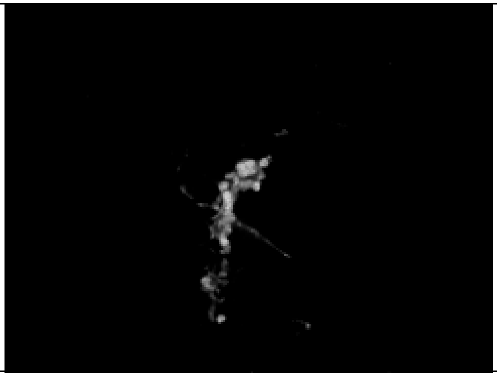	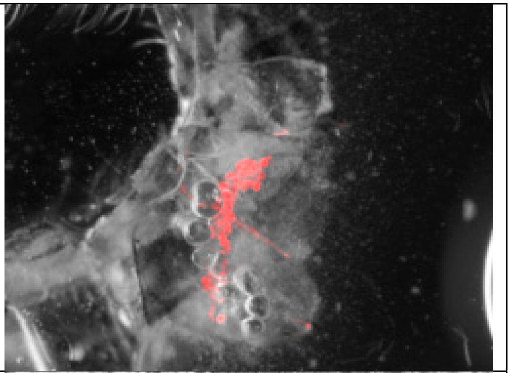
Abdomen / Fat body, hemocytes* / 24 HPE<br><br>* hemocytes and pericardial cell with iLacZ	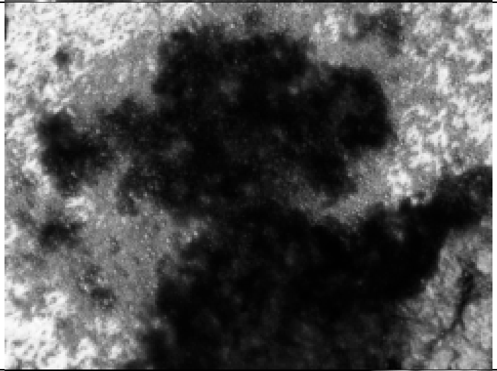	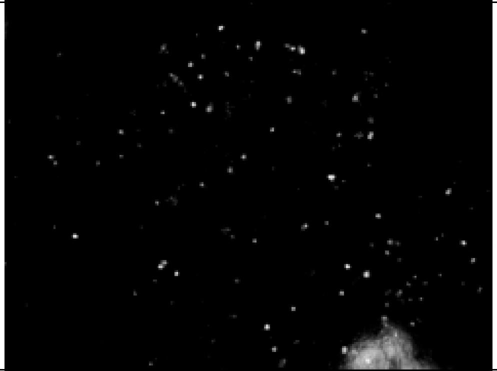	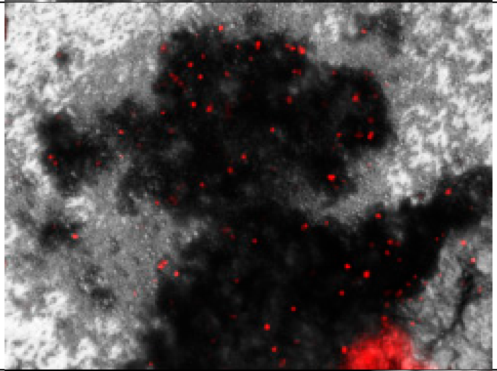
Abdomen / Malpighian tubules * / 72 HPE<br><br>* hemocytes with iLacZ	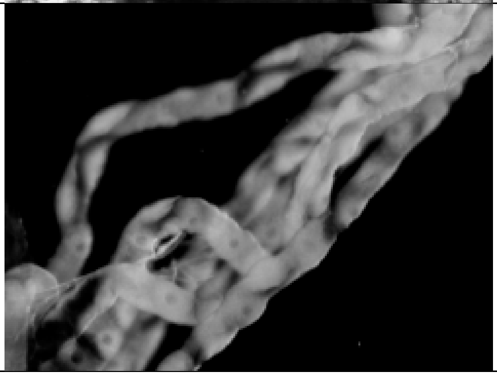	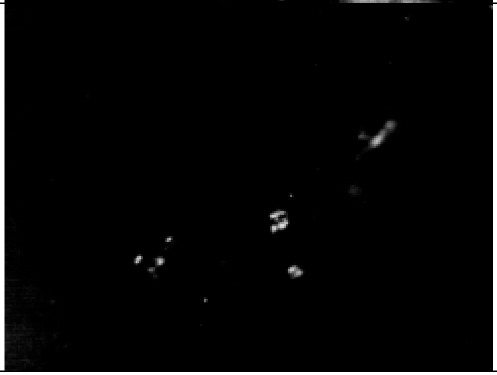	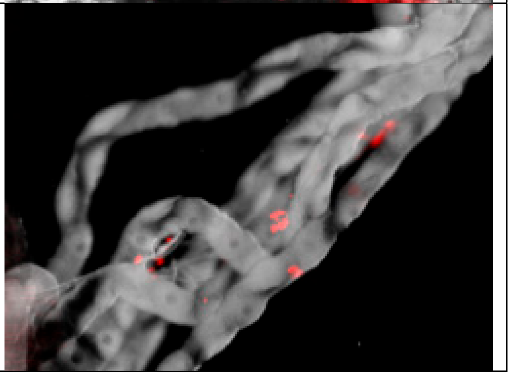

Abdomen / Diverticula * / 72 HPE<br><br>* Weak signal in lumen	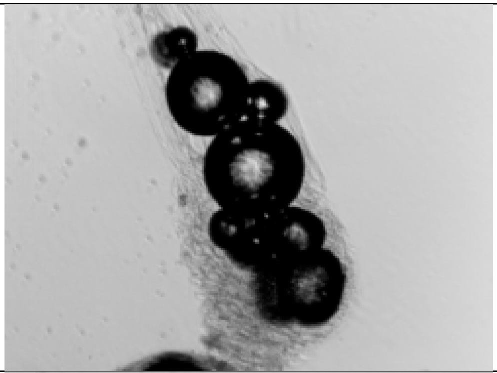	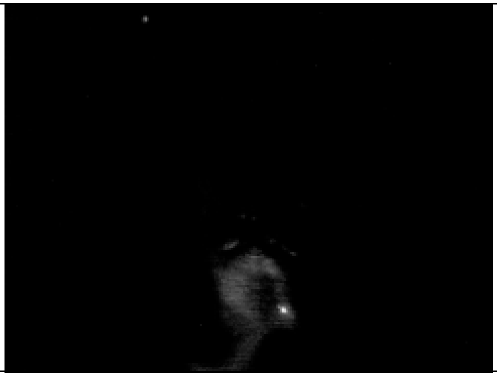	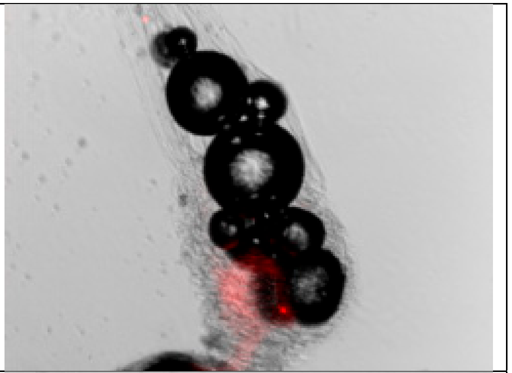
Abdomen / Diverticula * / 72 HPE<br><br>* Weak signal in lumen	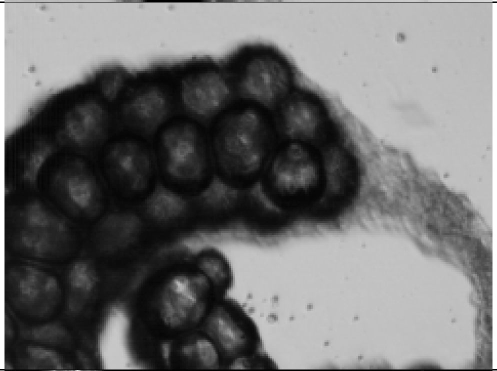	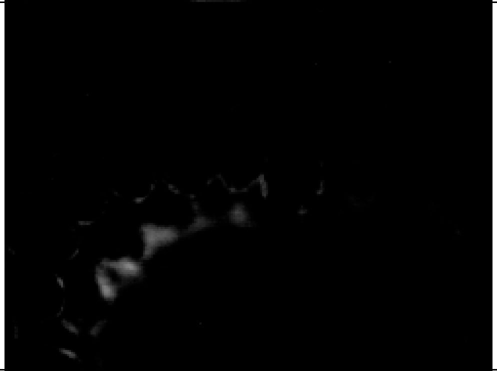	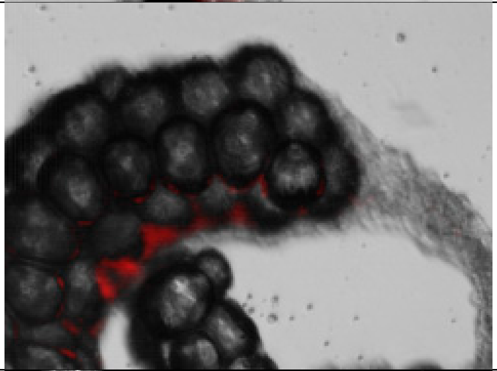
Abdomen / Central alimentary tract / 24 HPE	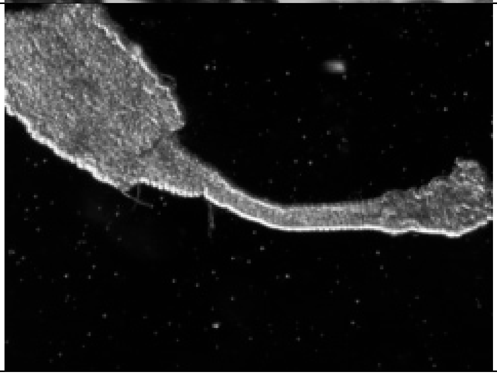	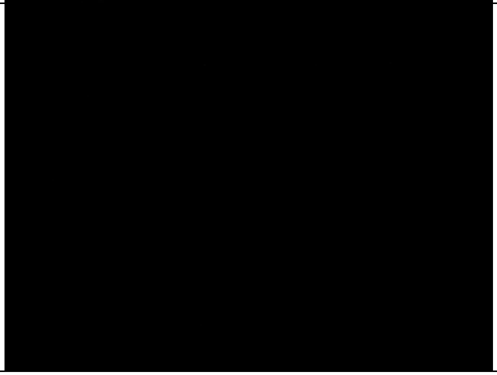	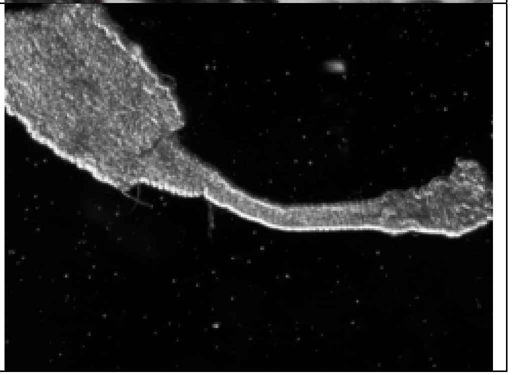

Abdomen / Central alimentary tract * / 24 HPE<br><br>* signal in lumen	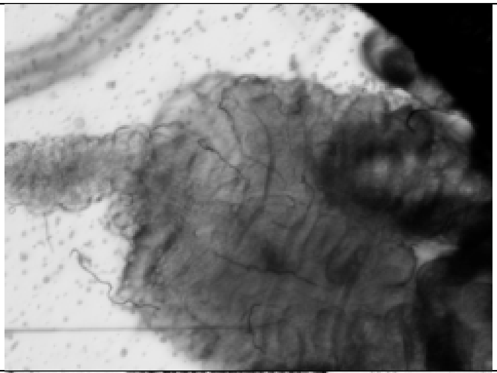	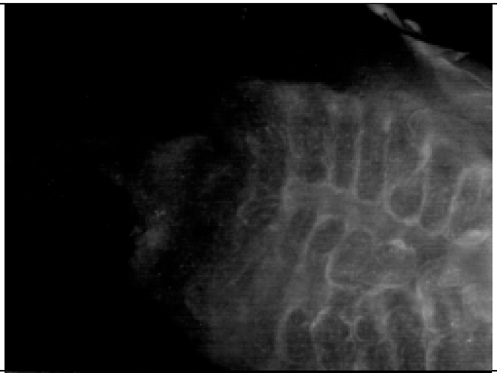	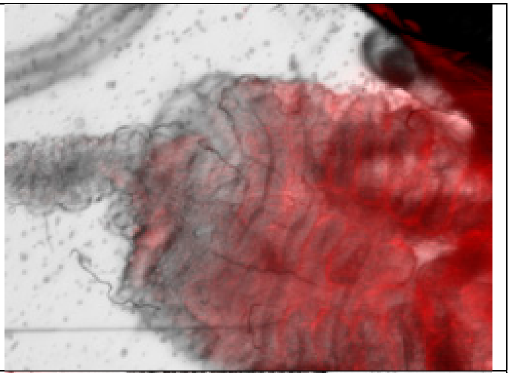
Abdomen / Alimentary tract * / 72 HPE<br><br>* hemocytes with iLacZ, Extracellular signal in hindgut lumen	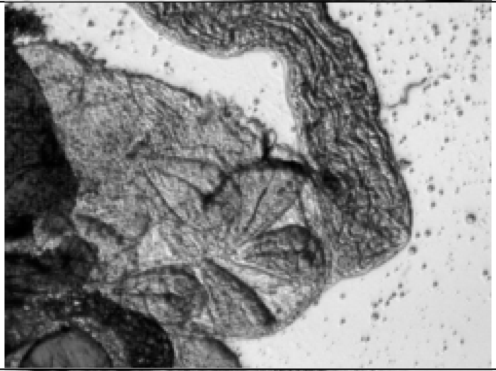	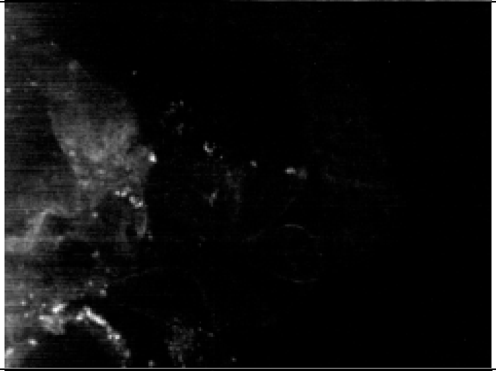	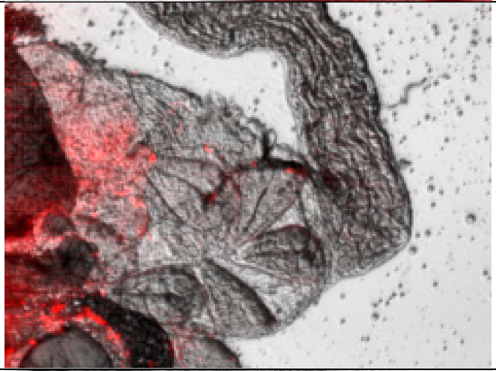
Abdomen / Alimentary tract, cuticle 72 HPE<br><br>* hemocytes with iLacZ, Extracellular signal in rectum lumen	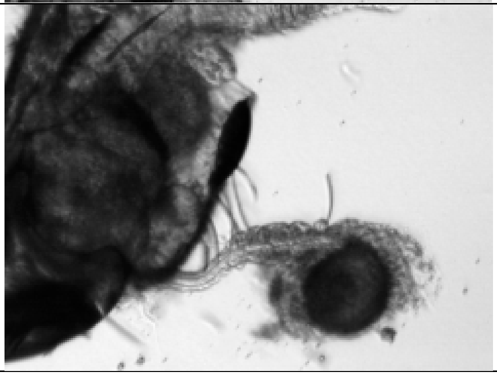	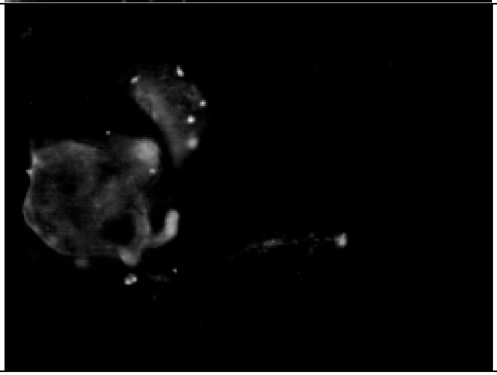	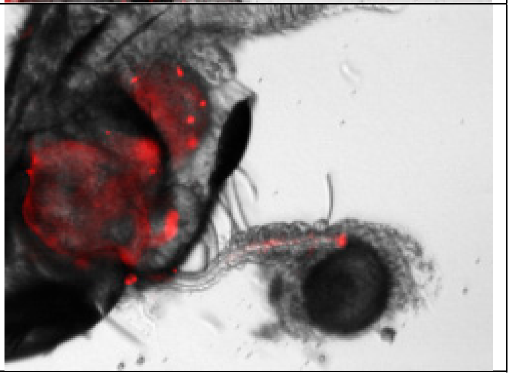

Abdomen / Ovary * / 24 HPE<br><br>* hemocytes and oocytes with iLacZ	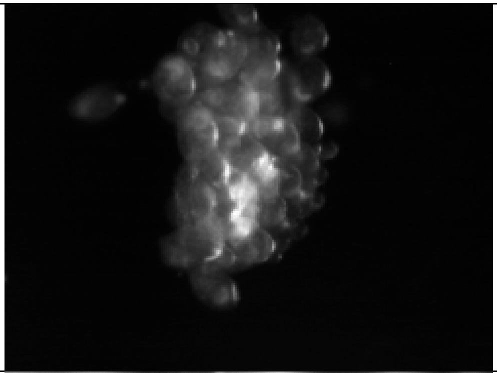	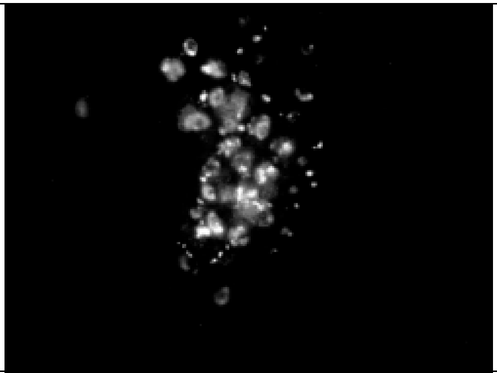	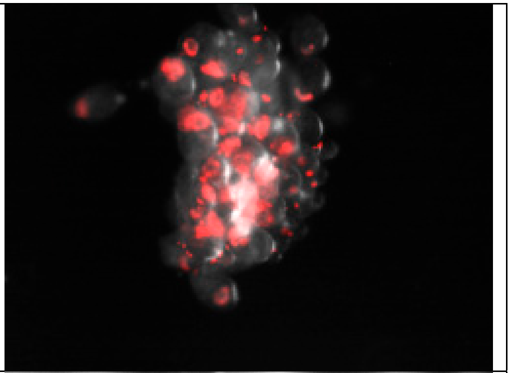
Un-injected negative controls for autofluorescence<br><br>Abdomen with no autofluorescence	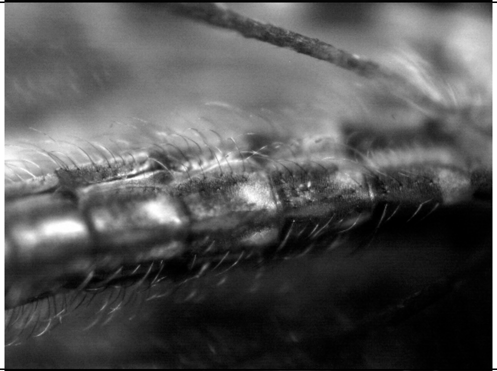	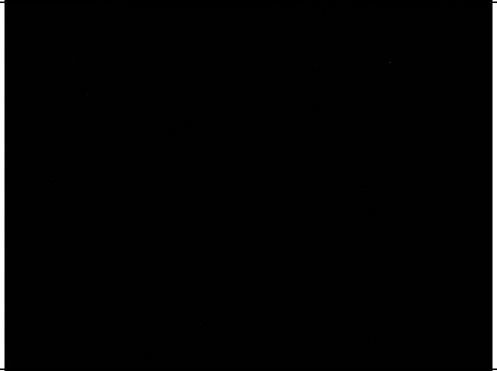	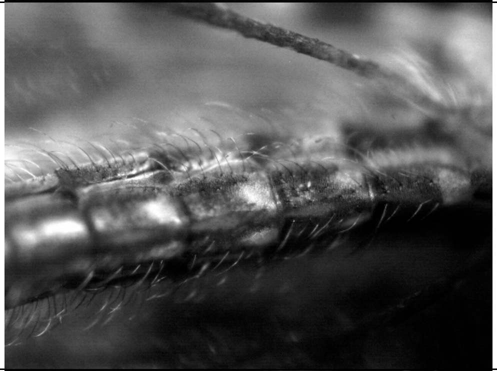
Un-injected negative controls for autofluorescence<br><br>Head with autofluoscence	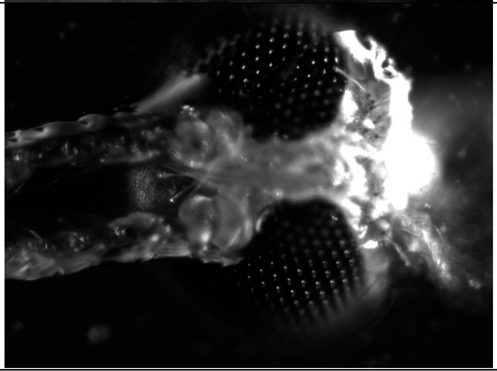	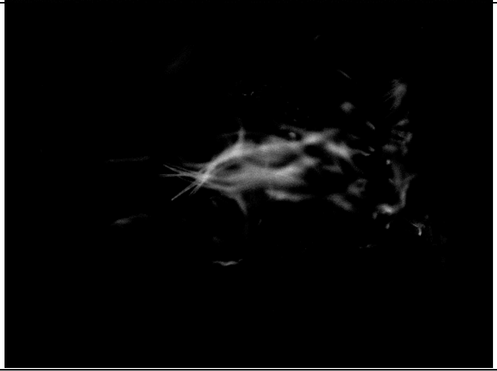	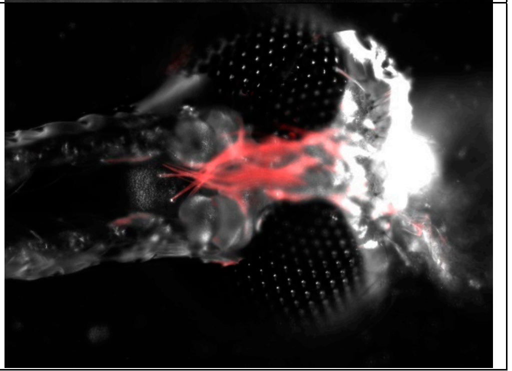

Un-injected negative controls for autofluorescence<br><br>Leg with cuticle autofluorescence	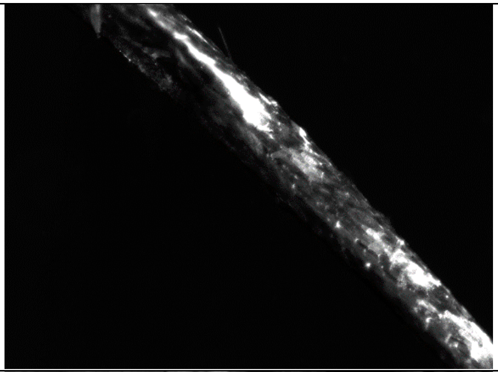	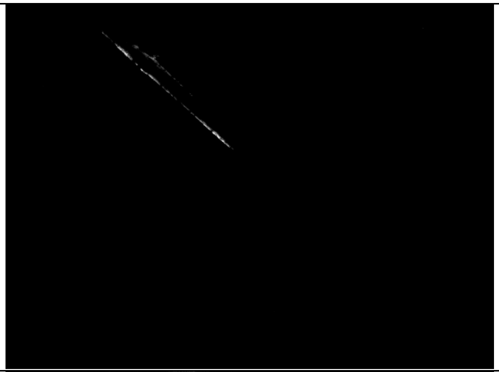	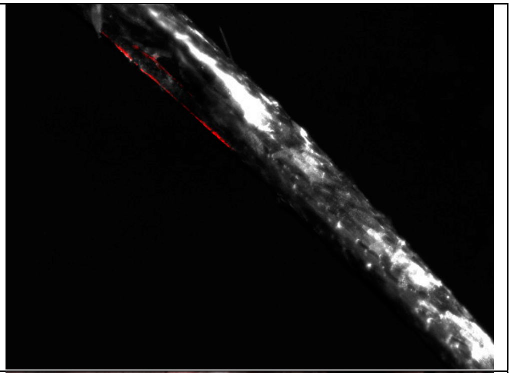
Un-injected negative controls for autofluorescence<br><br>Thorax with cuticle autofluorescence	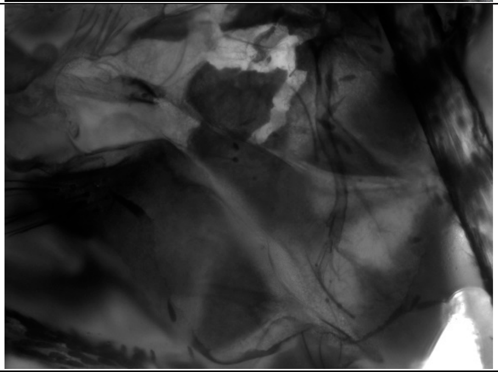	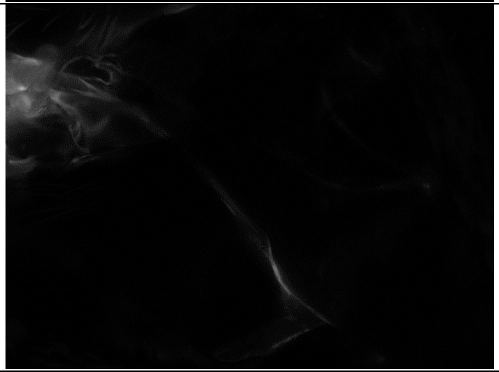	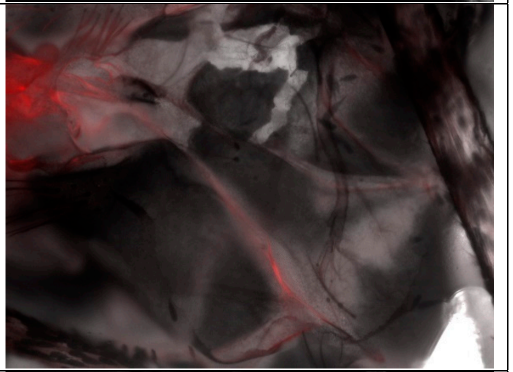
Un-injected negative controls for autofluorescence<br><br>Ovaries and Malphigian tubules with autofluorescence and background	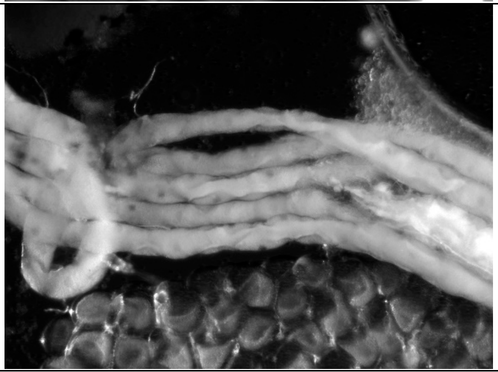	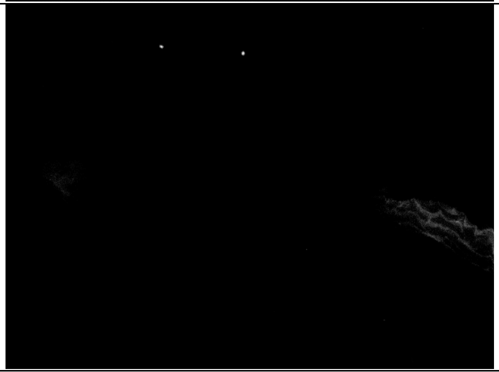	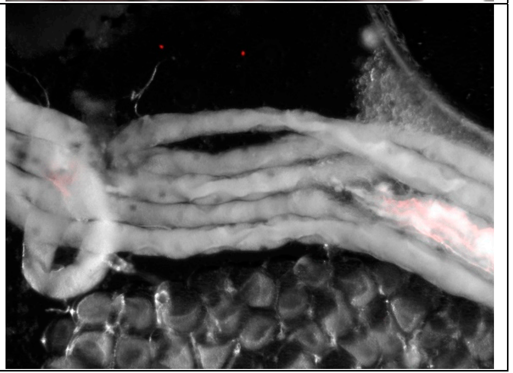

Un-injected negative controls for autofluorescence<br><br>Fat body	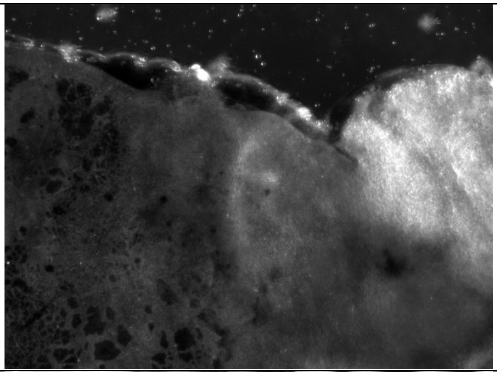	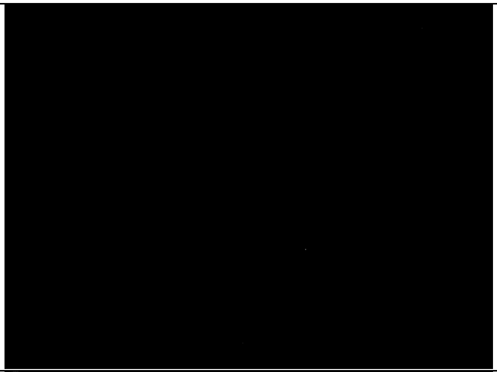	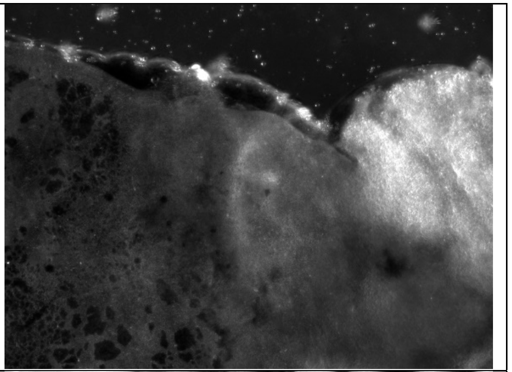
Un-injected negative controls for autofluorescence<br><br>Ovaries (post bloodfeeding)	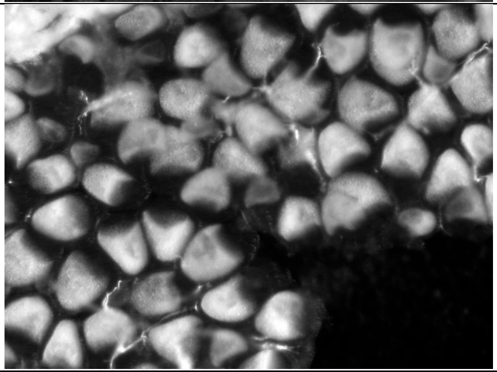	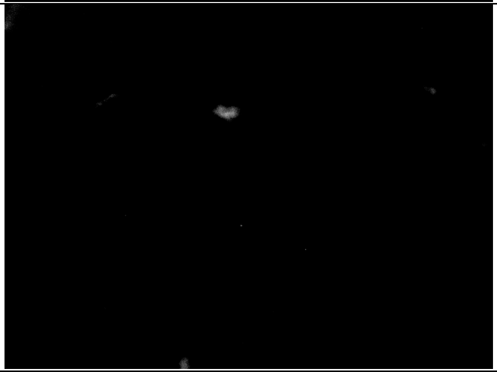	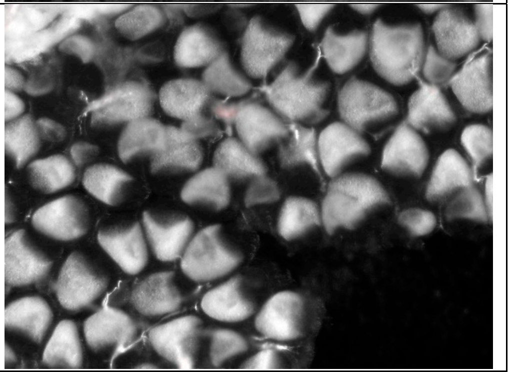

Figure S3 – Adult female *Cx. Pipiens* - intrathoracic injection

Tissue	Bright Field	RNAi trigger	Merge
Head / signal at injection site / 24 HPE	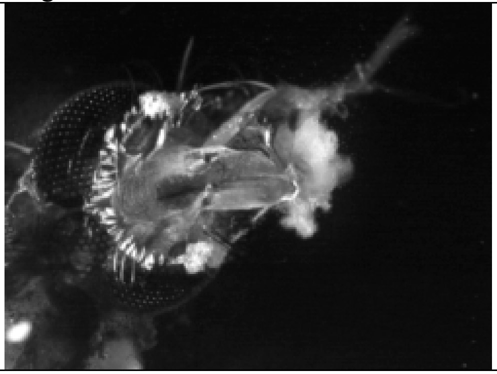	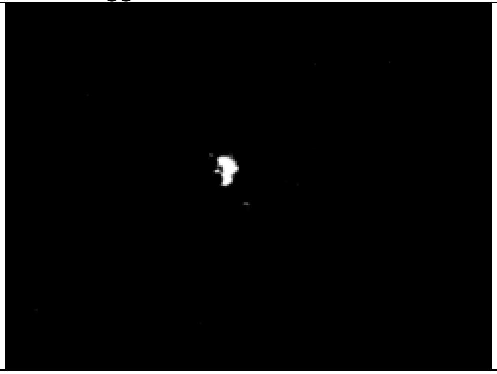	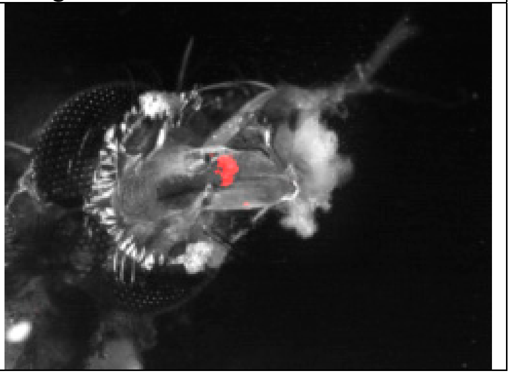

Thorax / auto-fluorescence in cuticle, hemocytes associated with tracheole / 24 HPE	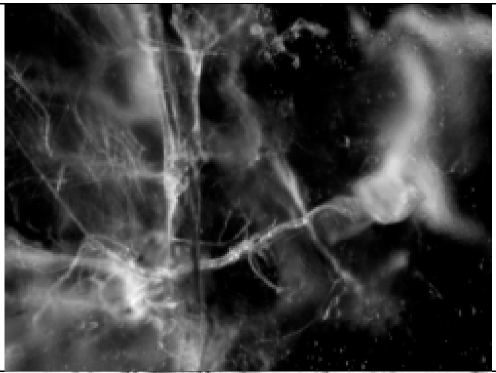	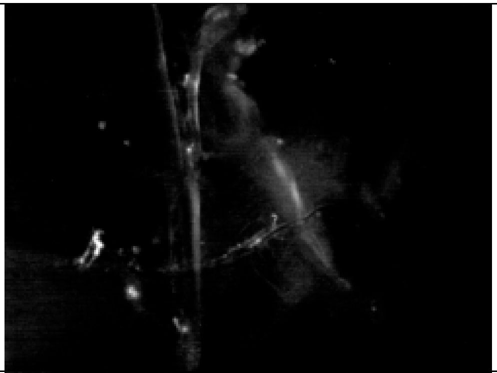	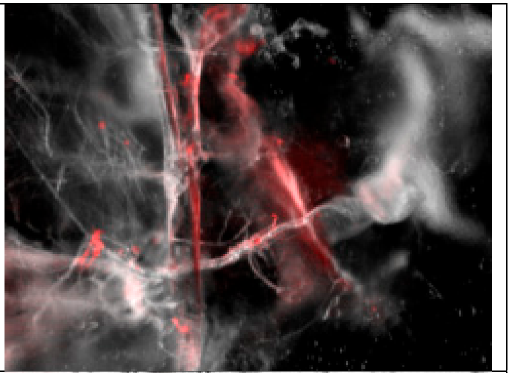
Thorax / fat body, hemocytes, and pericardial cell / 24 HPE	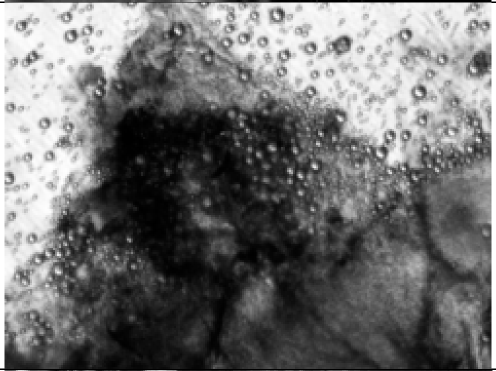	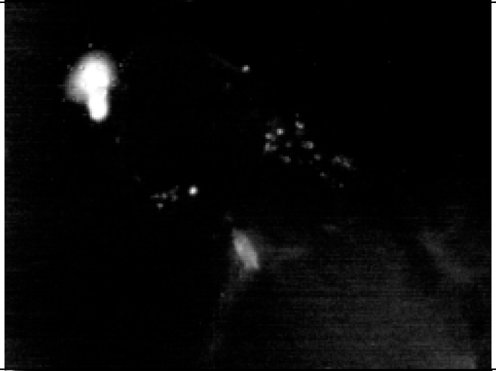	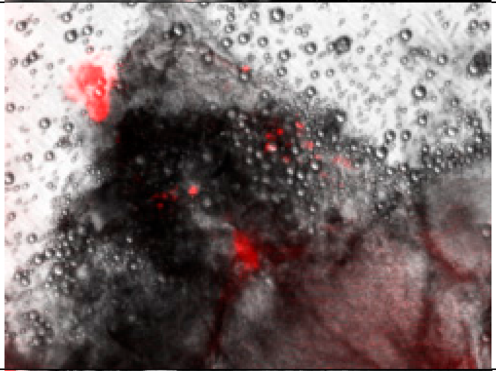
Abdomen / pericardial cells / 72 HPE	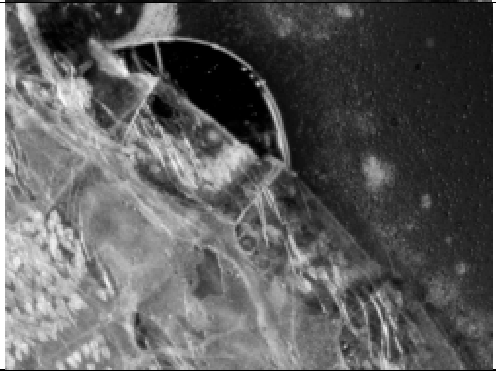	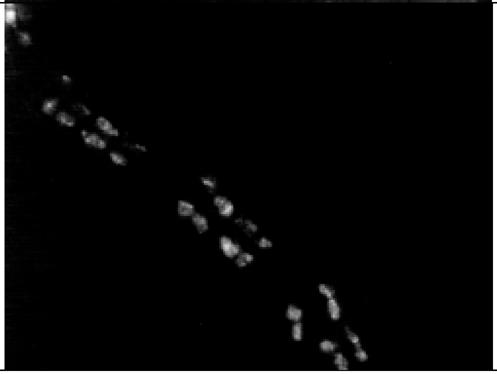	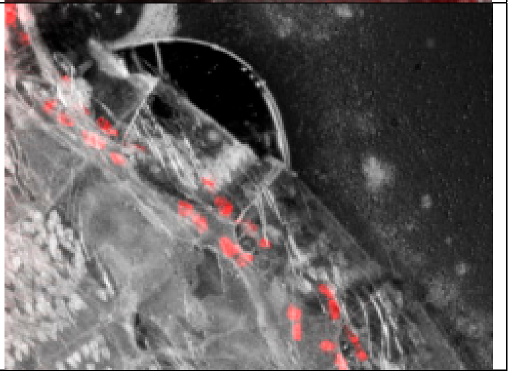

Fat body / pericardial cells / 120 HPE	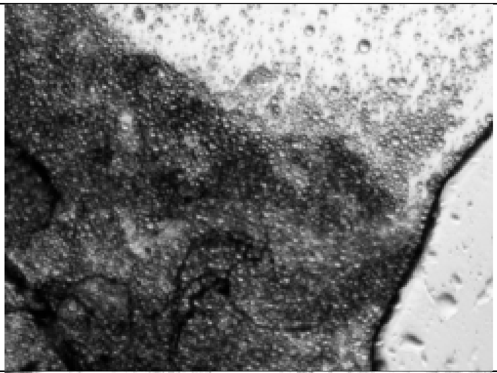	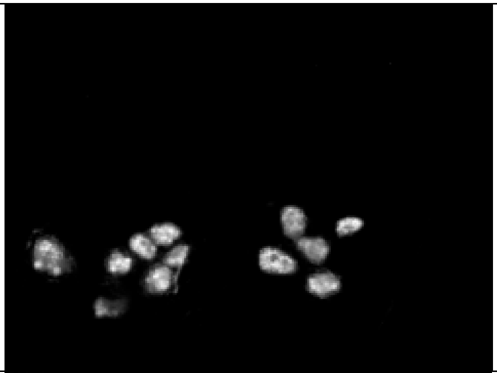	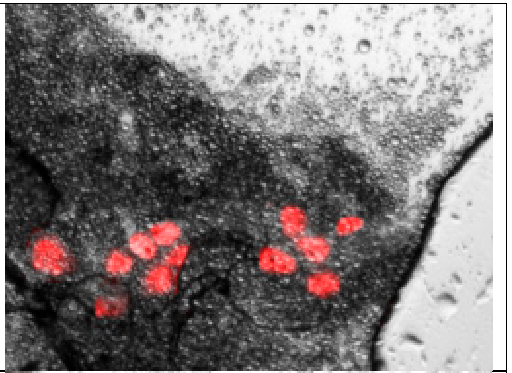
Fat body / hemocytes / 24 HPE	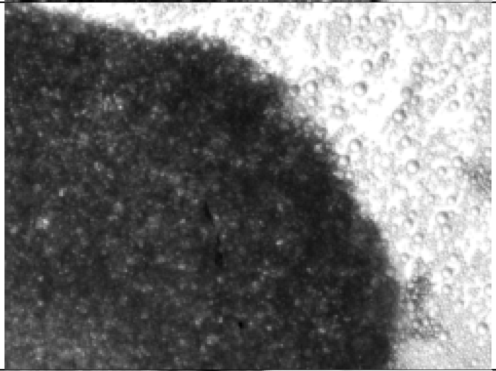	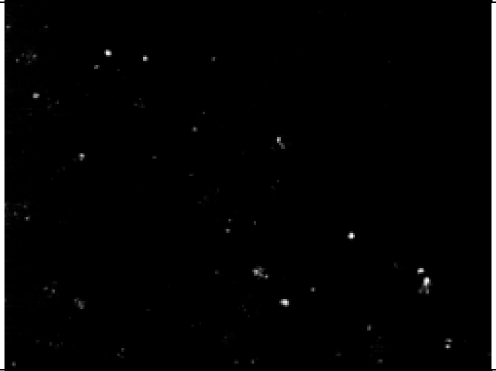	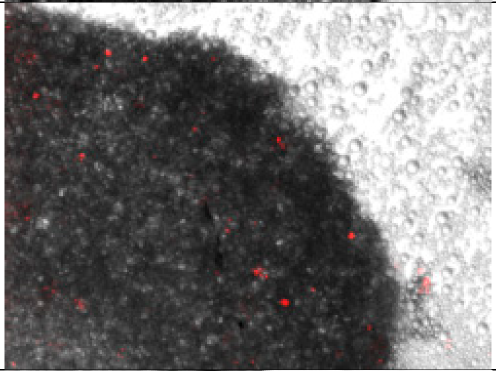
Malpighian tubules / hemocytes bound to tracheoles / 72 HPE	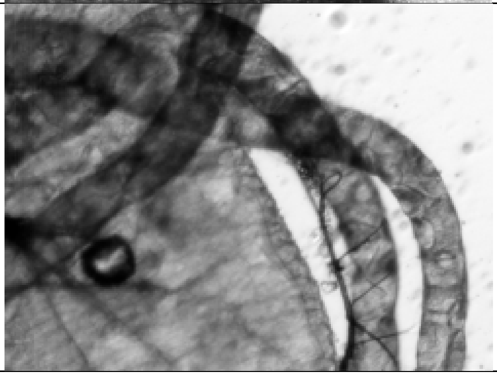	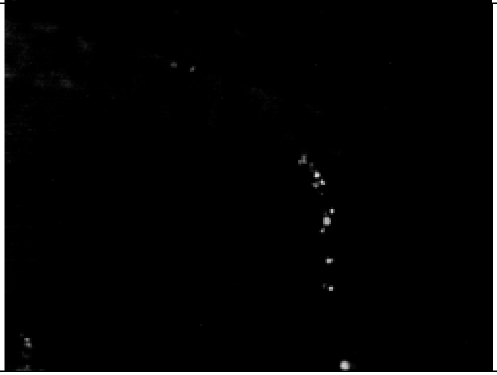	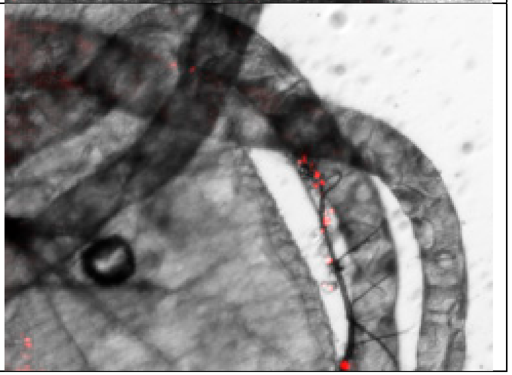

Diverticula with auto-fluorescent signal / 24 HPE	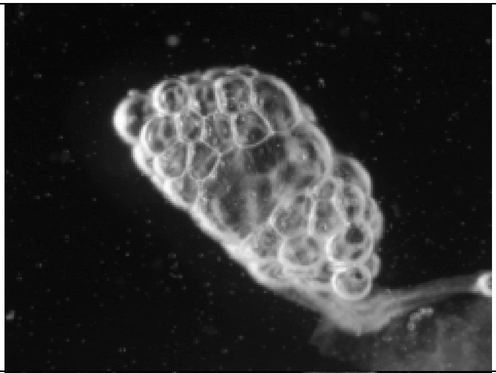	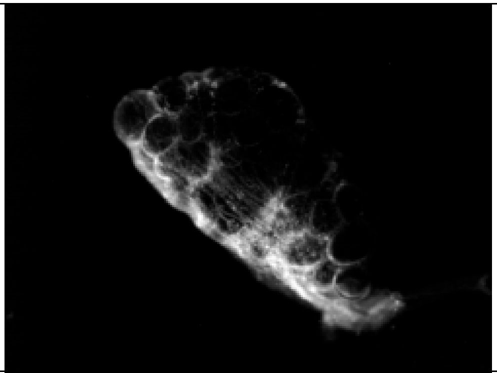	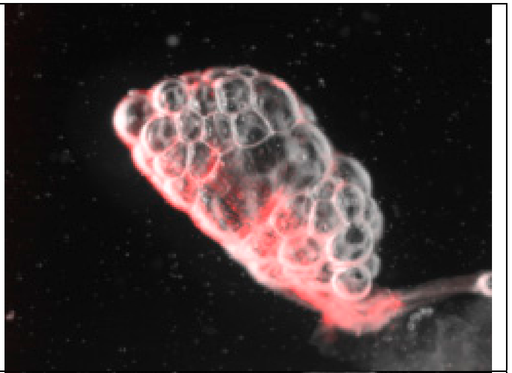
Alimentary tract with no signal, extracellular Malpighian tubules / 72 HPE	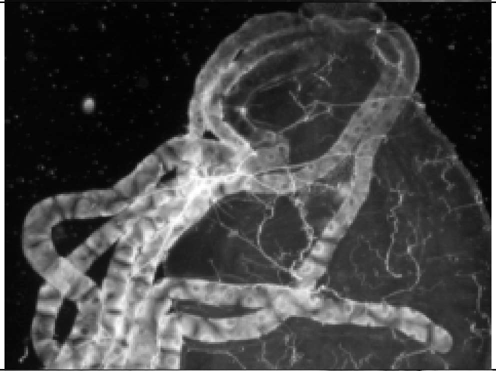	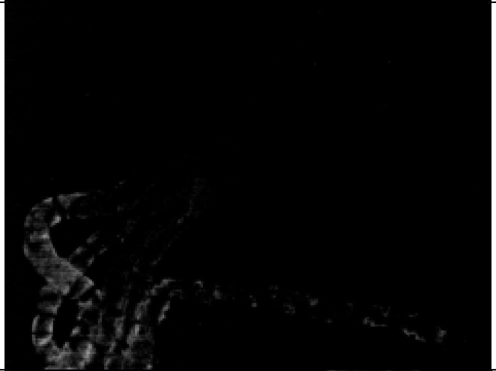	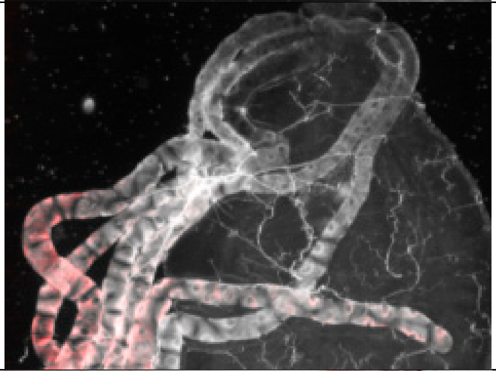
Extracellular signal / foregut and midgut lumen / 24 HPE	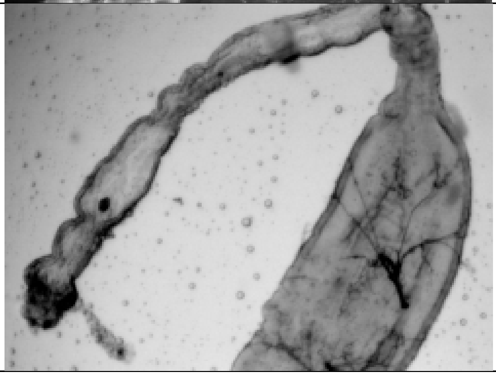	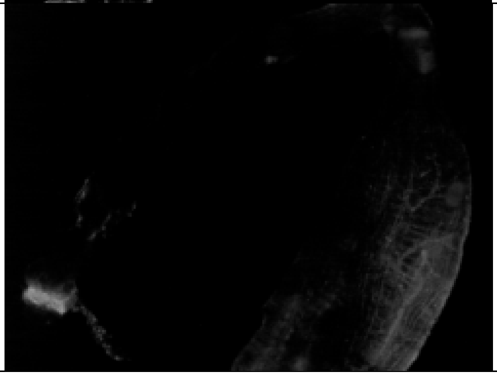	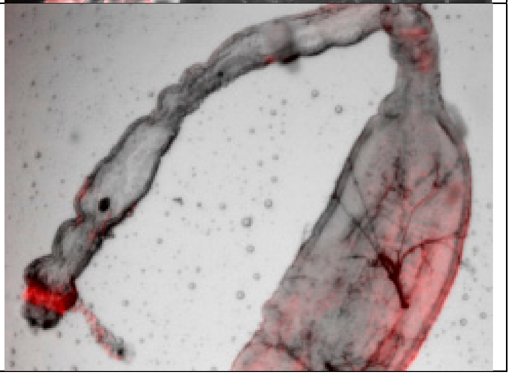

Extracellular signal / hindgut lumen / 24 HPE	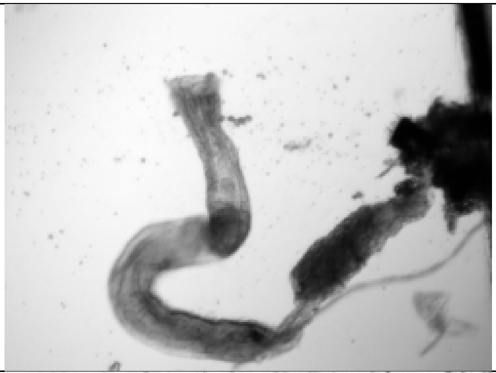	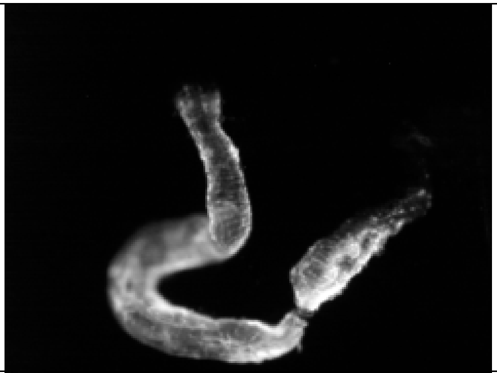	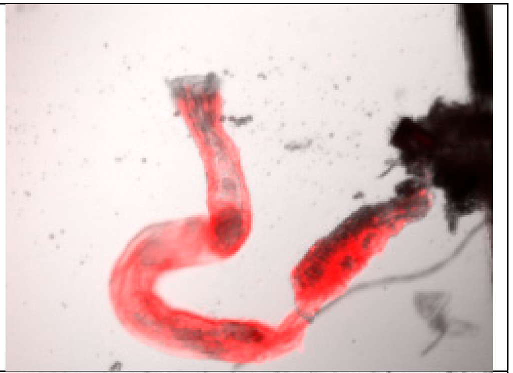
Extracellular signal / rectum lumen and hemocytes / 72 HPE	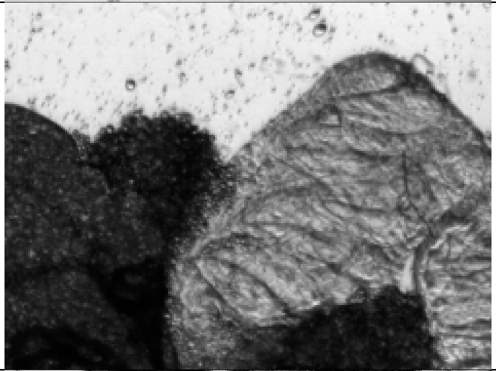	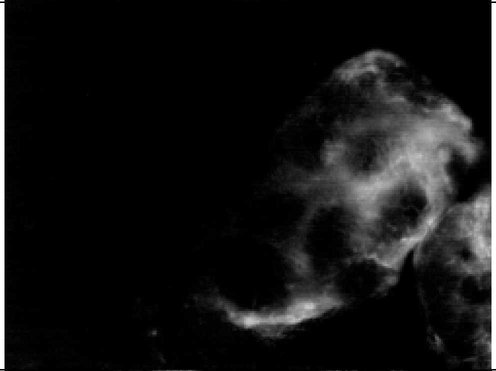	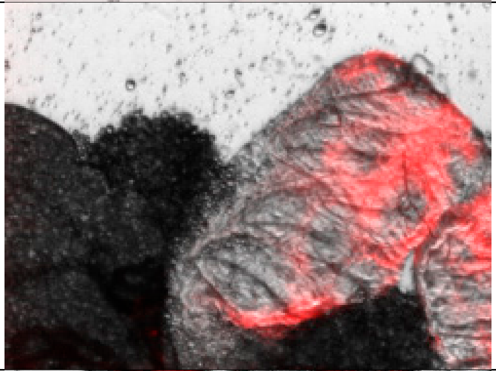
Ovary / fluorescence in hemocytes and oocytes / 24 HPE	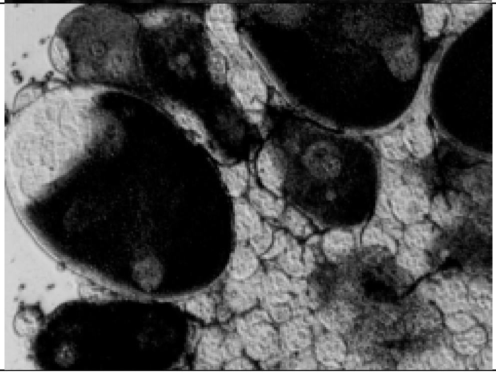	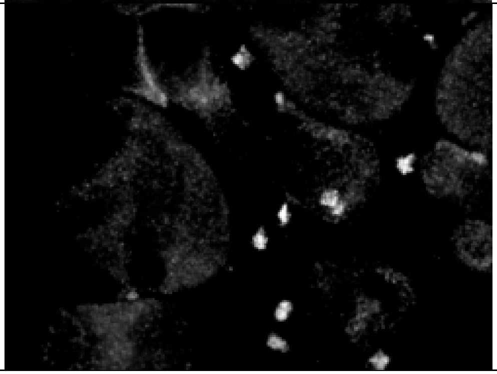	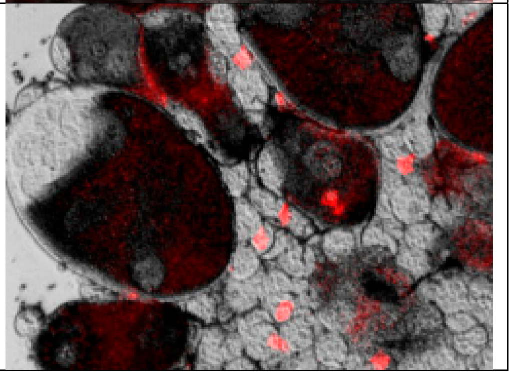

Ovary / fluorescence in hemocytes / 72 HPE	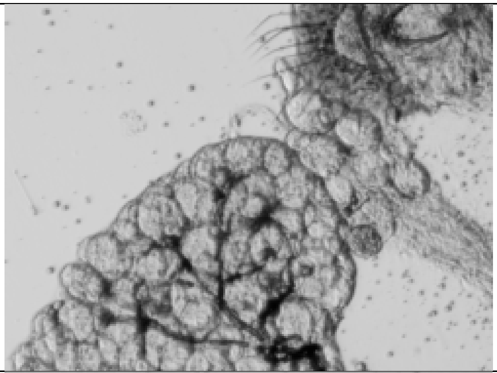	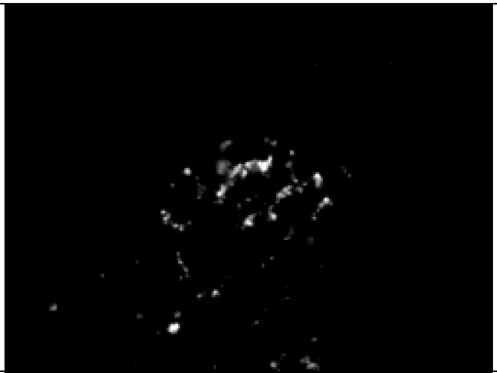	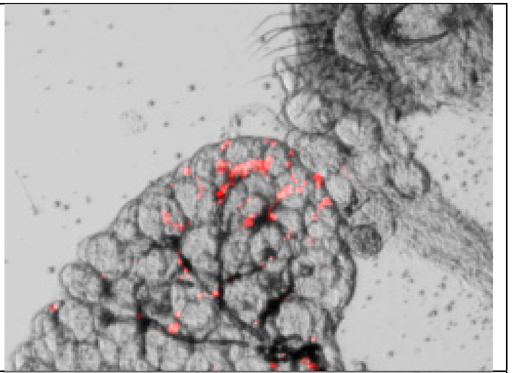
Ovary / fluorescence in hemocytes / 24 HPE	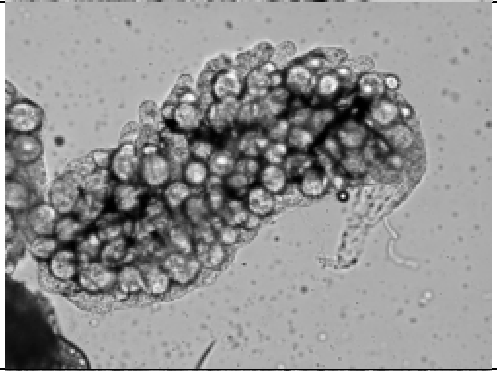	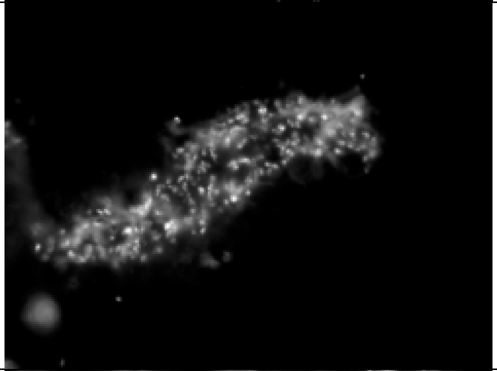	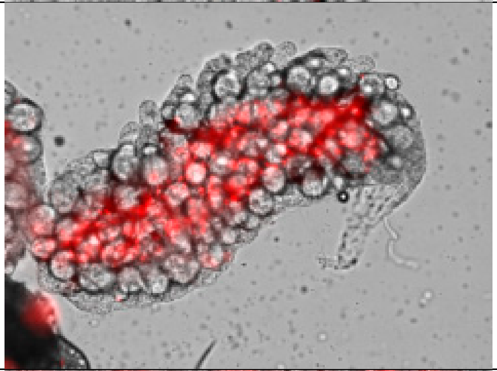
Ovary / fluorescence in oocytes / 24 HPE	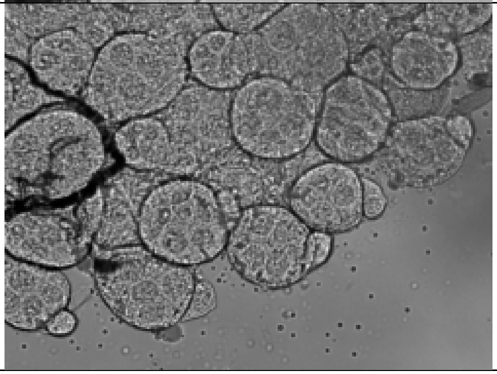	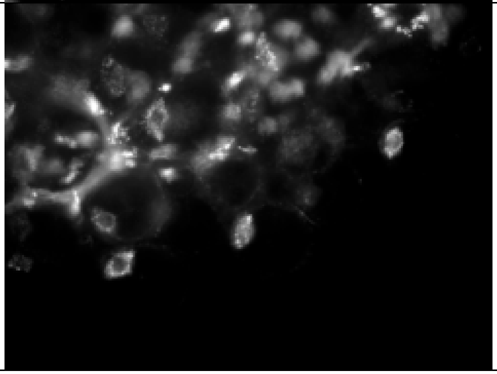	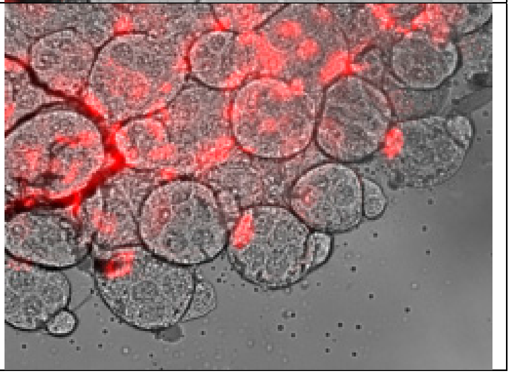

Un-injected negative controls for autofluorescence<br><br>Diverticula with autofluorescence noted	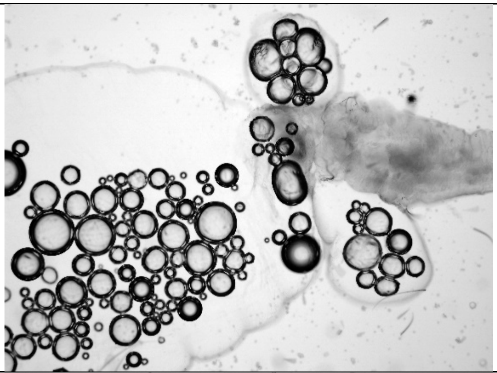	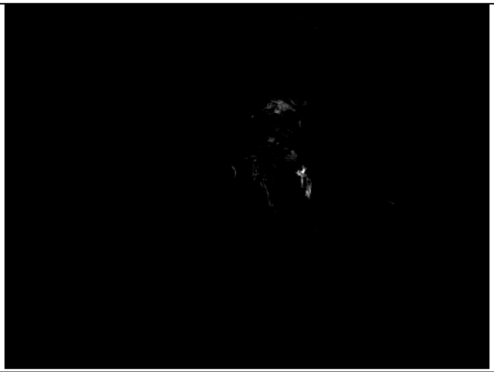	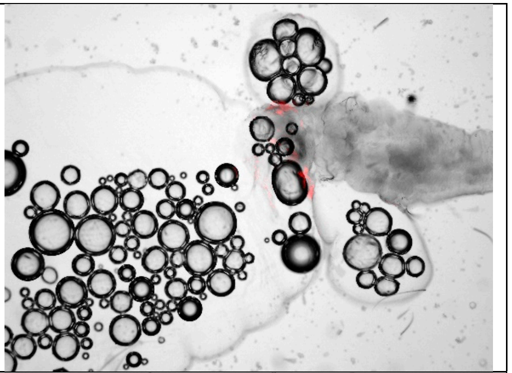
Un-injected negative controls for autofluorescence<br><br>Midgut	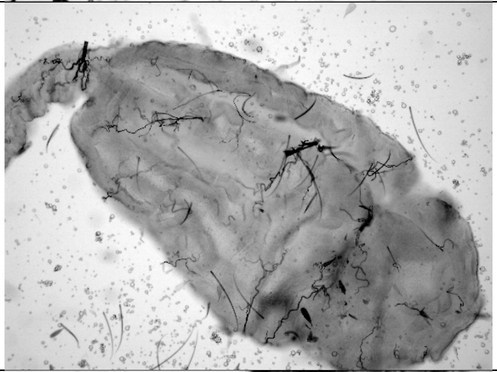	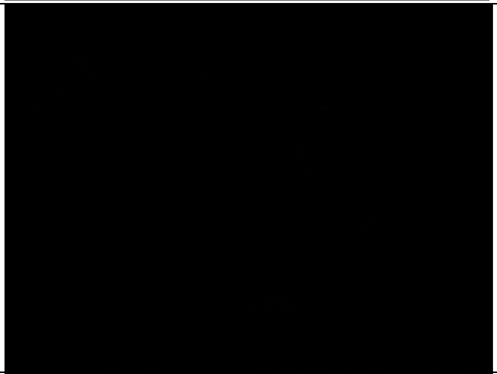	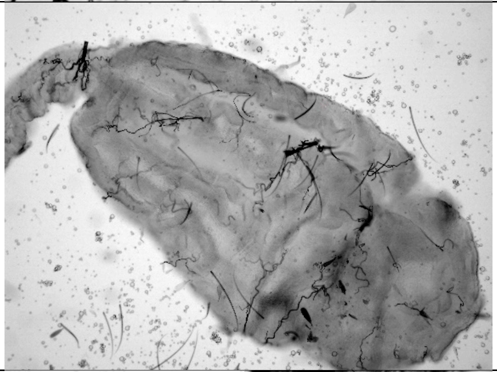
Un-injected negative controls for autofluorescence<br><br>Abdomen with autofluorescence from flexible membrane noted	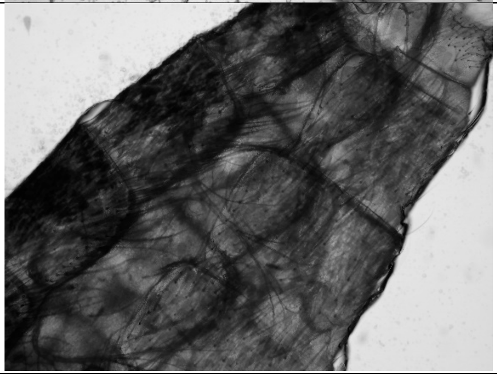	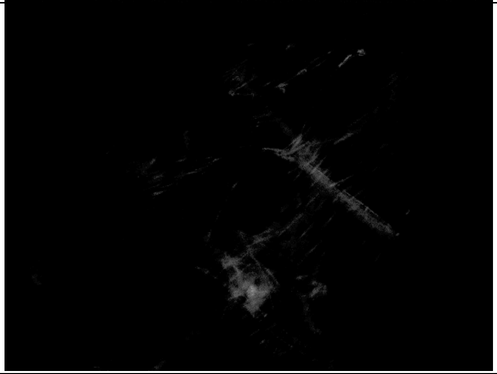	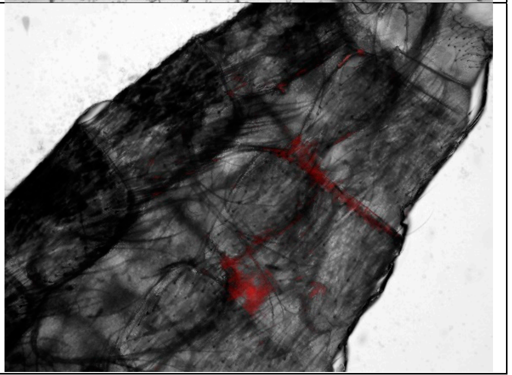

Un-injected negative controls for autofluorescence<br><br>Leg with cuticle autofluorescence	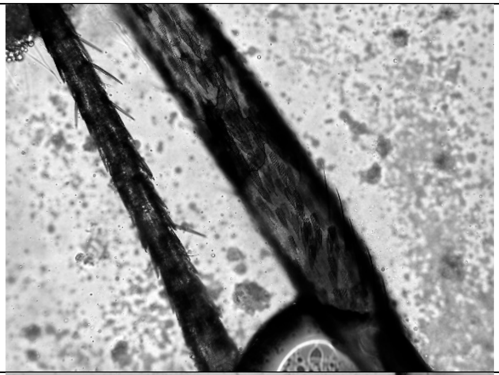	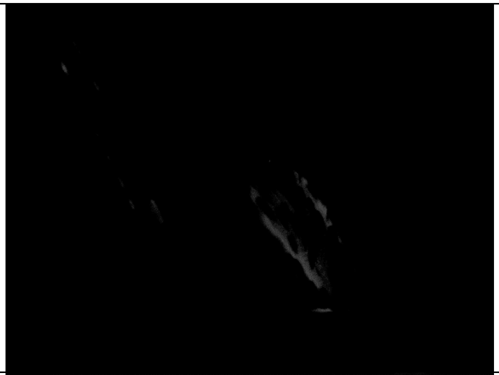	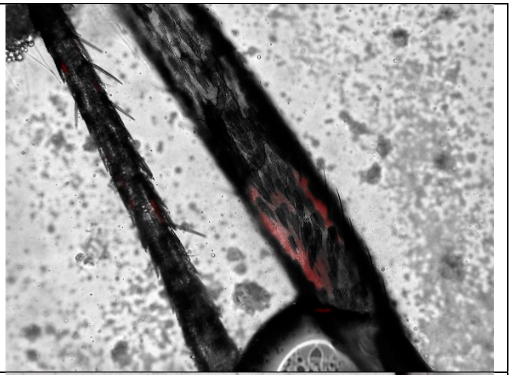
Un-injected negative controls for autofluorescence<br><br>Ovaries, Malphigian tubules, and abdominal cuticle with autofluorescence in cuticle (weak with general background showing)	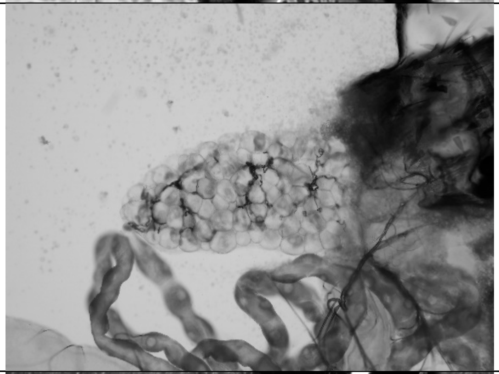	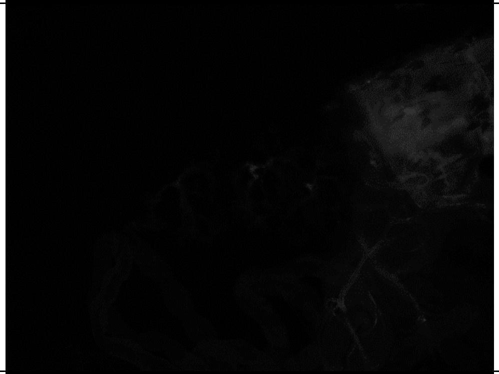	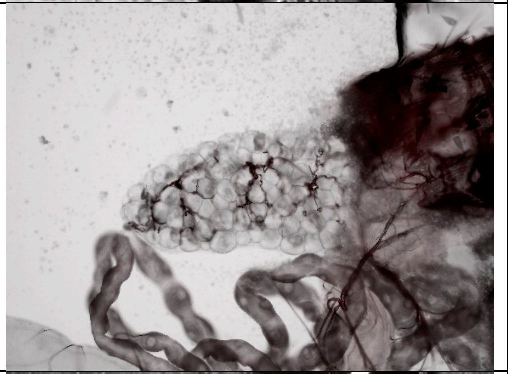
Un-injected negative controls for autofluorescence<br><br>Fat body	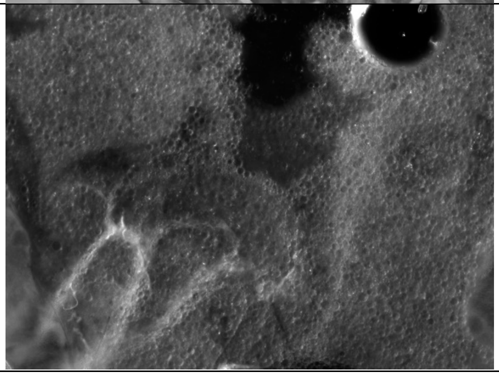	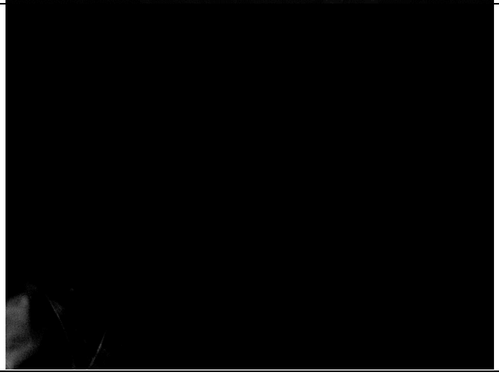	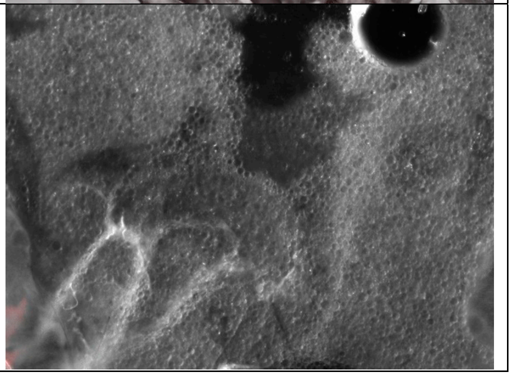

Un-injected negative controls for autofluorescence<br><br>Ovaries (post blood-feeding)	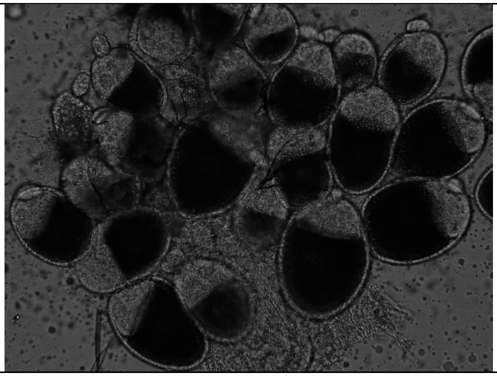	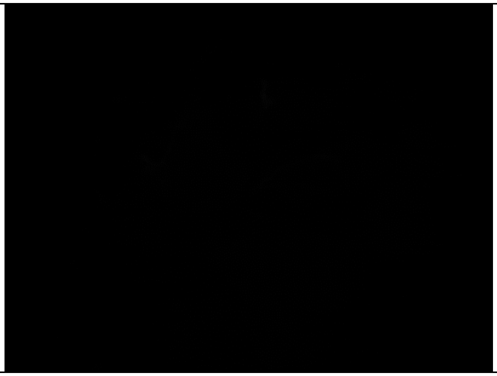	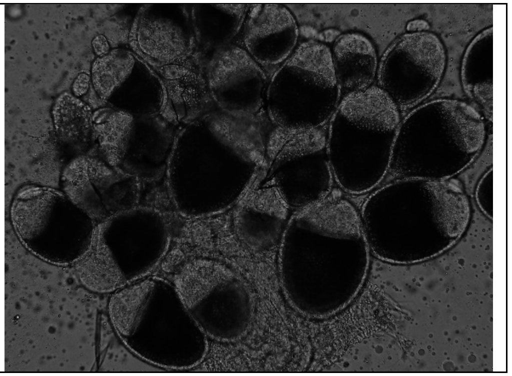

Figure S4 – Adult female *Ae. aegypti* 25-48 hours post blood/meal - intrathoracic injection

Tissue	Bright Field	RNAi trigger	Merge
Thorax / pericardial cells and auto-fluorescence in cuticle / 24 HPE	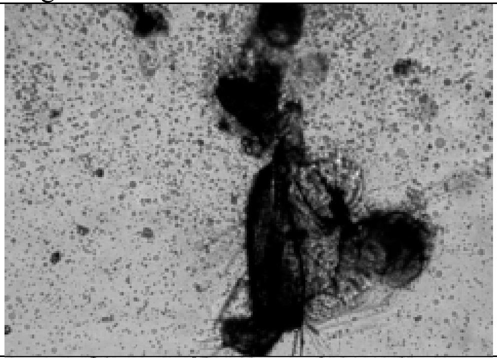	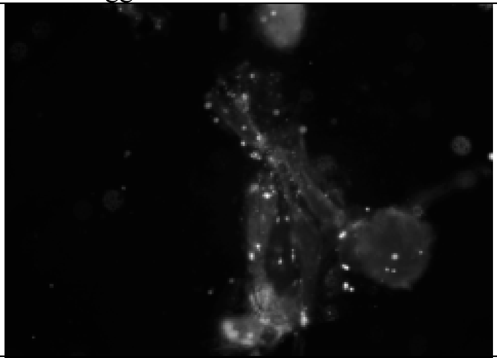	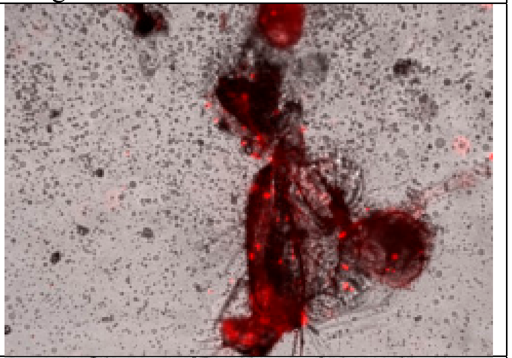
Thorax / pericardial cells and auto-fluorescence / 1 HPE	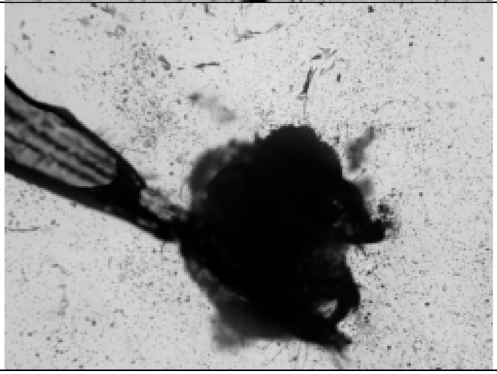	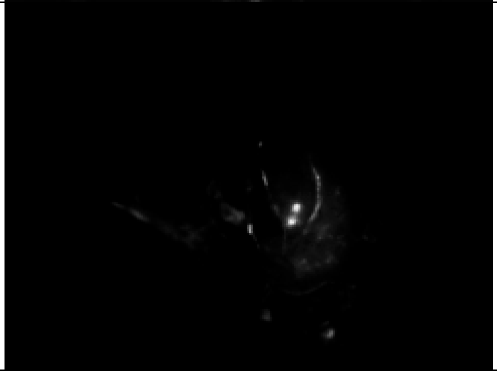	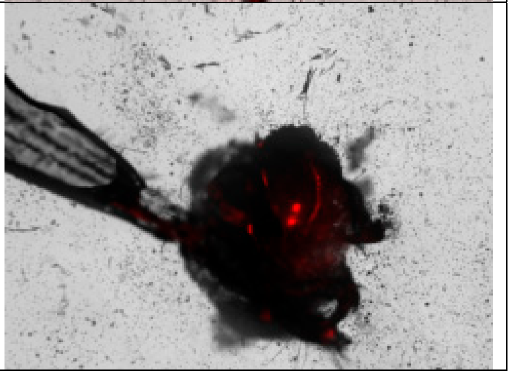

Abdomen / pericardial cells / 1 HPE	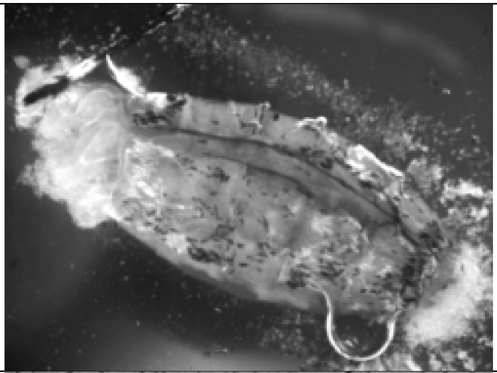	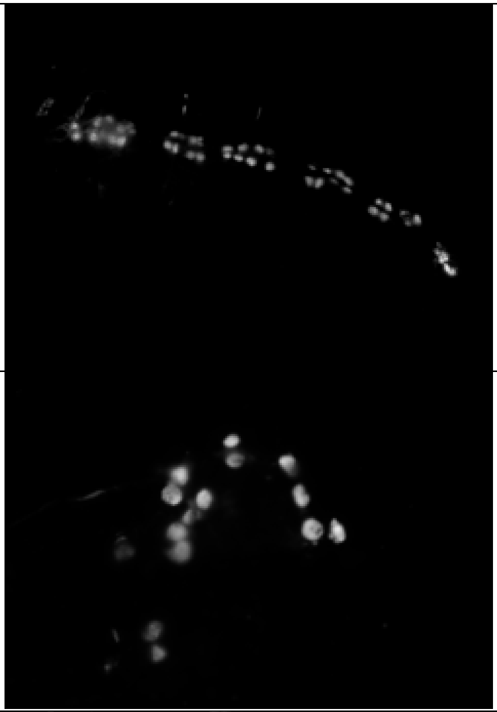	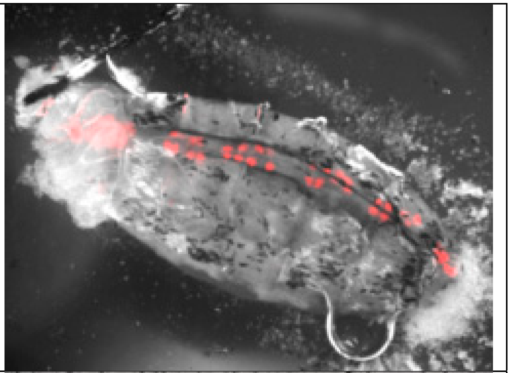
Abdomen / pericardial cells / 24 HPE	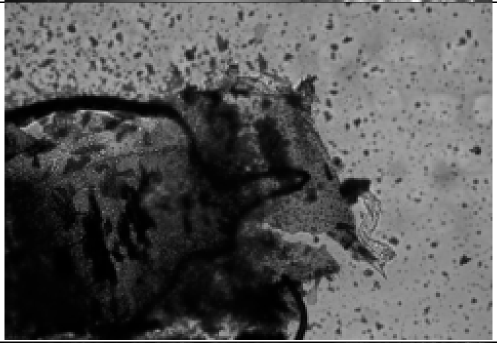		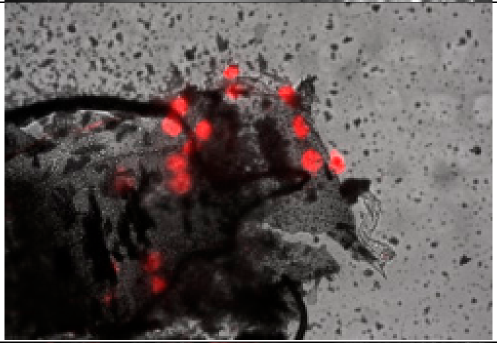
Fat body / hemocytes and ovarian follicles (bottom right) / 1 HPE	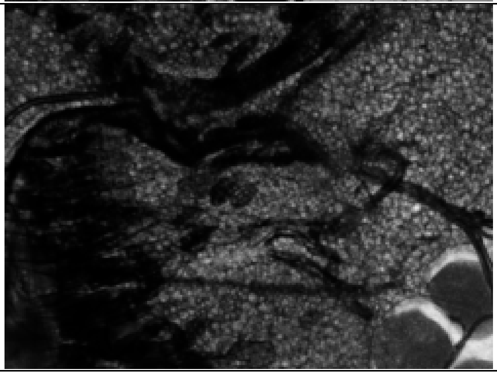	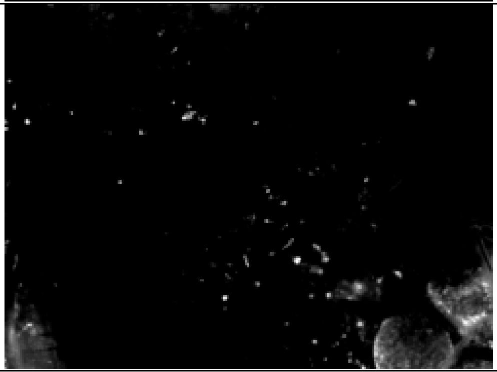	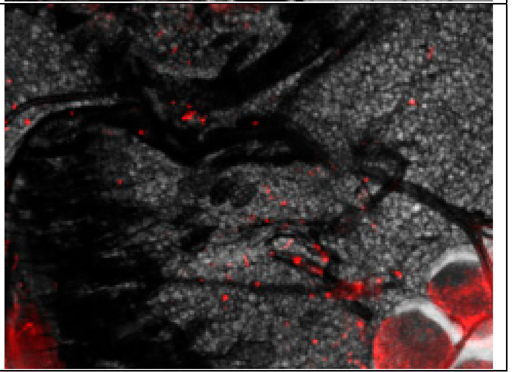

Alimentary tract with no signal and hemocytes / 1 HPE	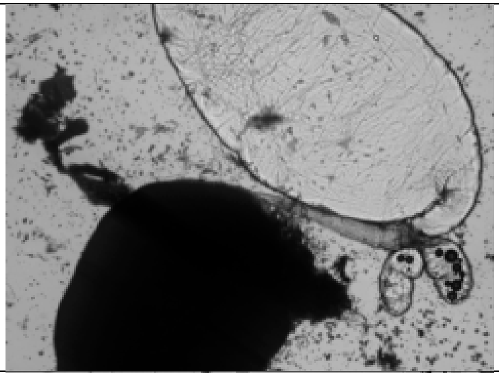	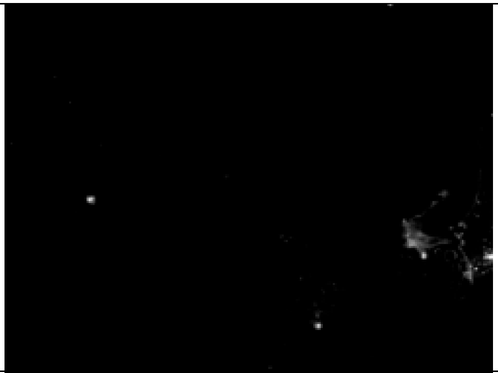	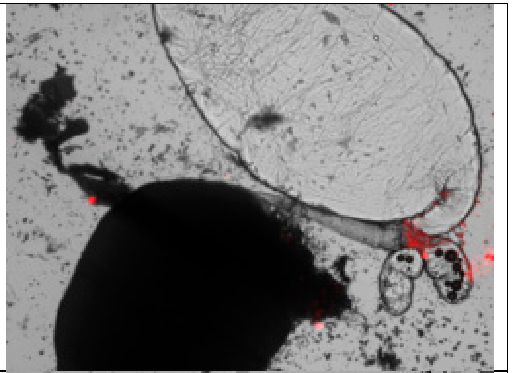
Hindgut and rectum with auto-fluorescent signal / 1 HPE	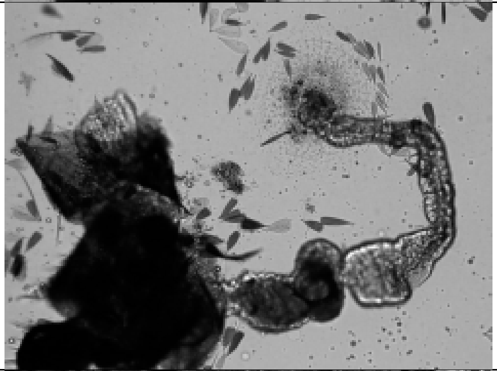	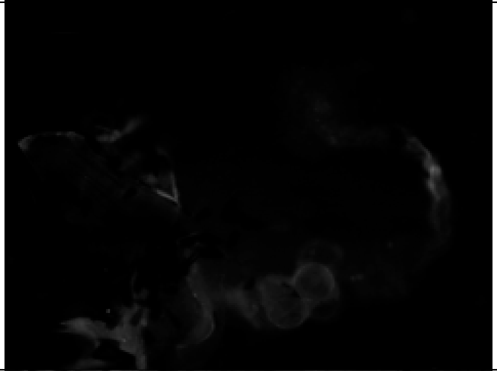	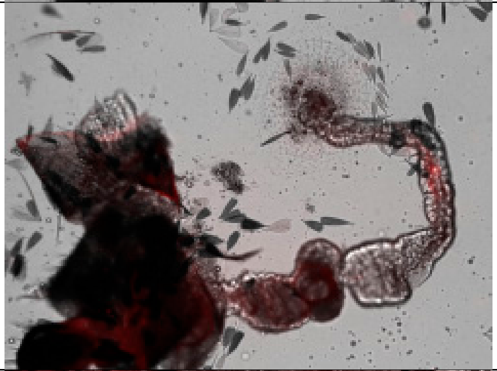
Ovary and fat body / hemocytes and oocytes with bright small atretic follicles / 1 HPE	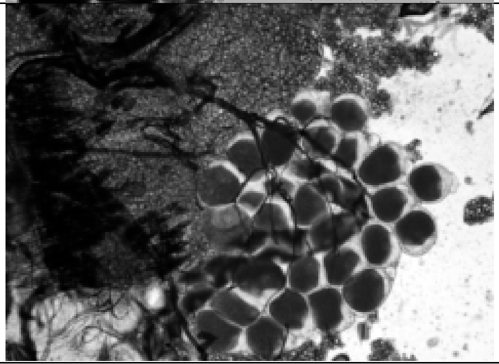	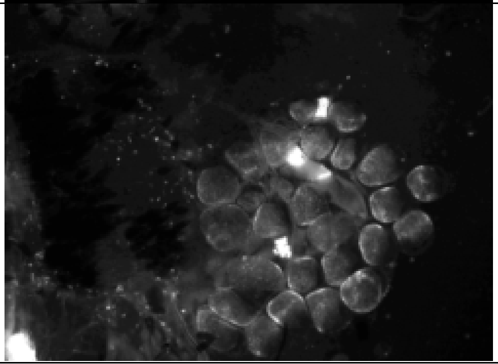	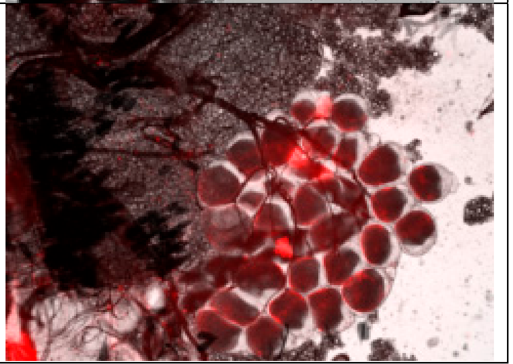

Ovary / small atretic and large healthy follicles / 24 HPE	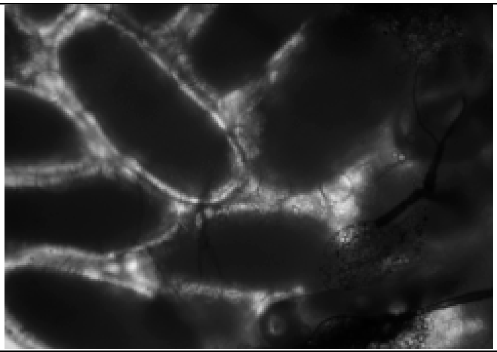	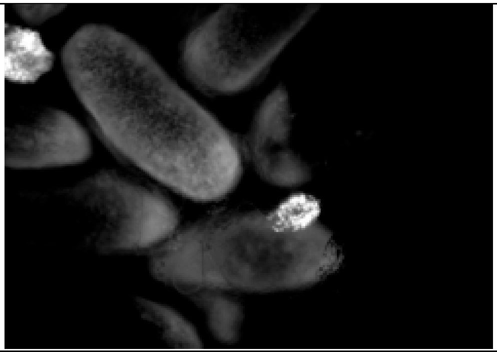	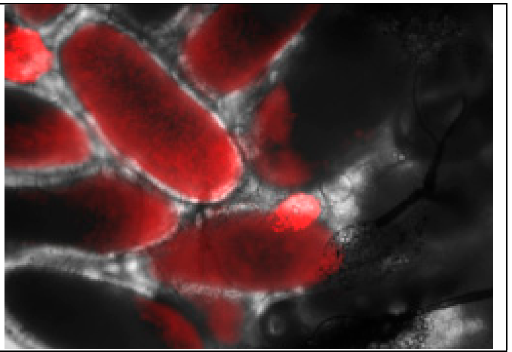
Ovary / fluorescence in oocytes / 24 HPE	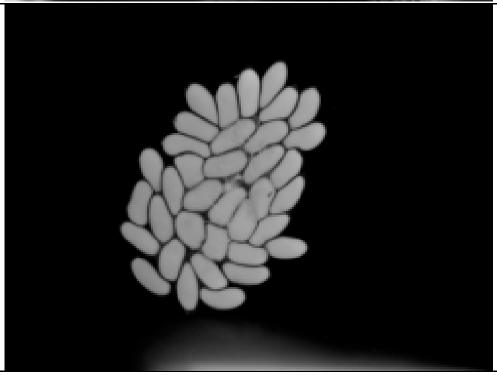	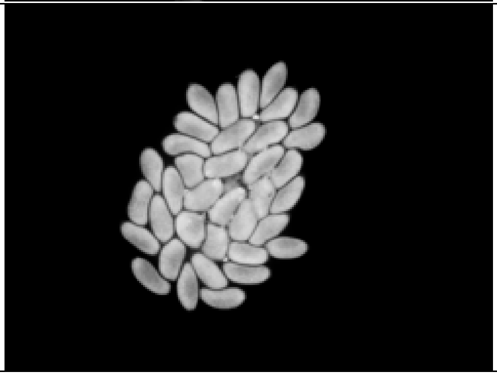	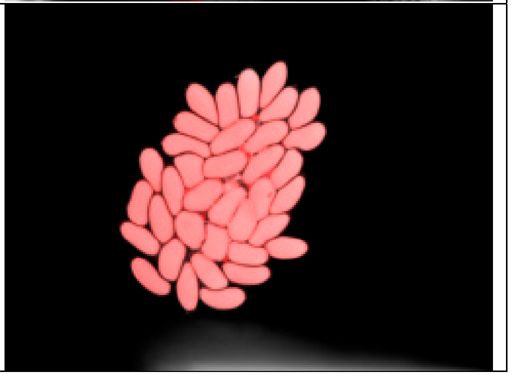

Figure S5 – Adult female *An. gambiae* 25-48 hours post blood/meal - intrathoracic injection

Tissue	Bright Field	RNAi trigger	Merge
Head / injection size and hemocytes / 24 HPE	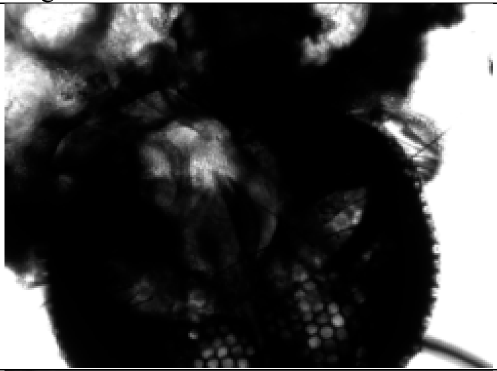	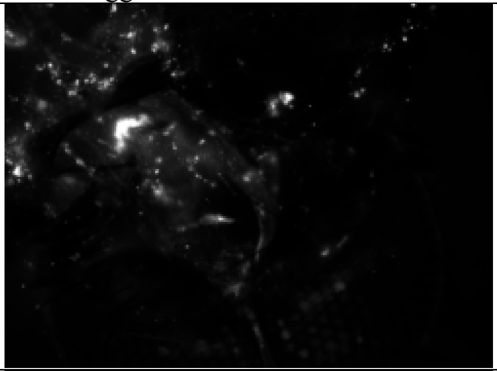	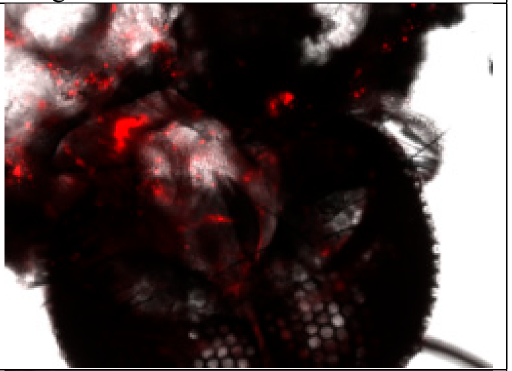
Head / hemocytes in palps / 24 HPE	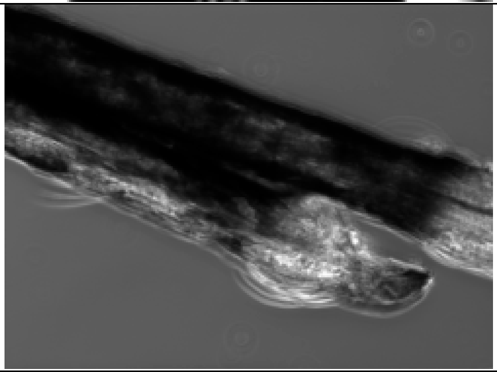	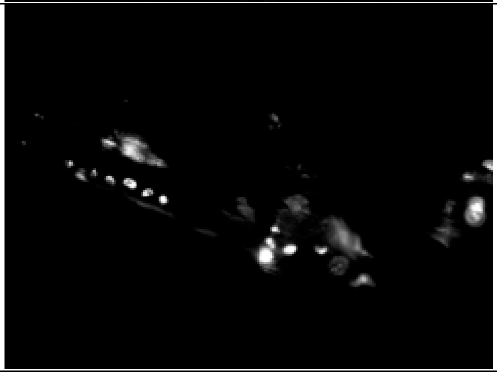	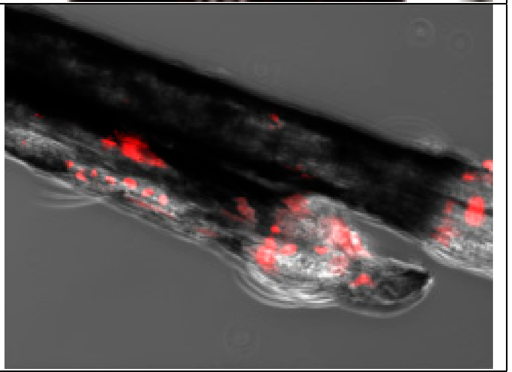
Abdomen / pericardial cells / 24 HPE	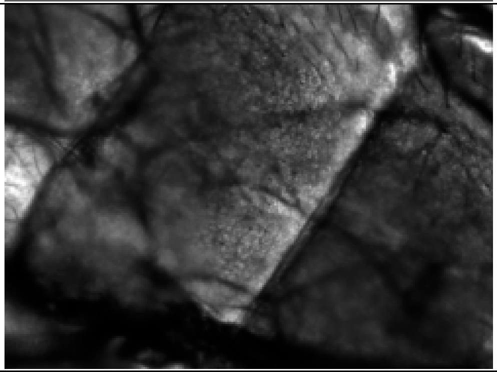	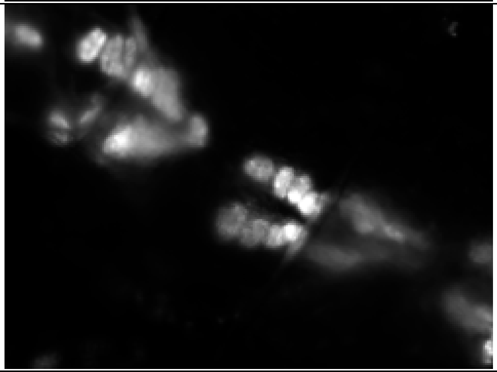	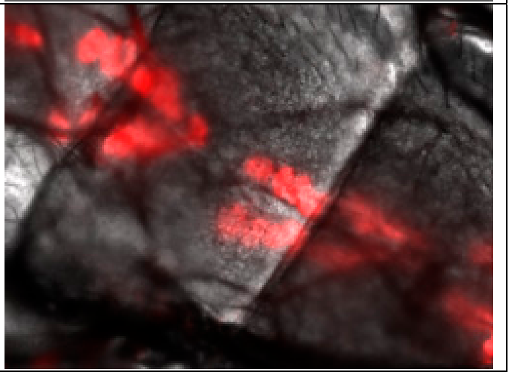

Leg / hemocytes / 1 HPE	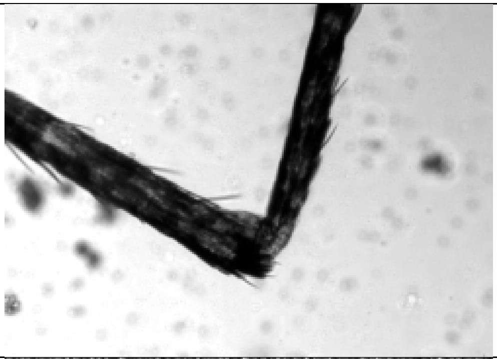	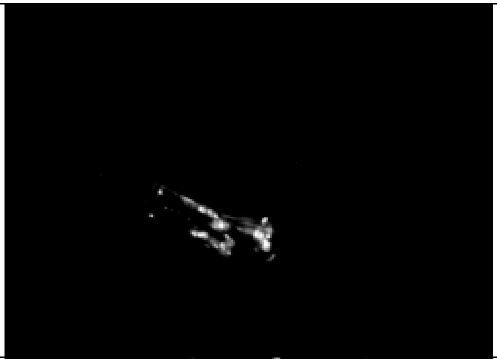	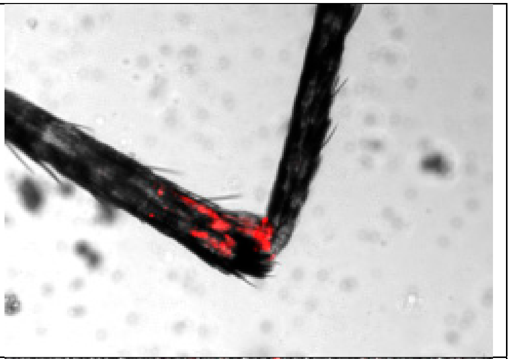
Fat body / hemocytes and / 1 HPE	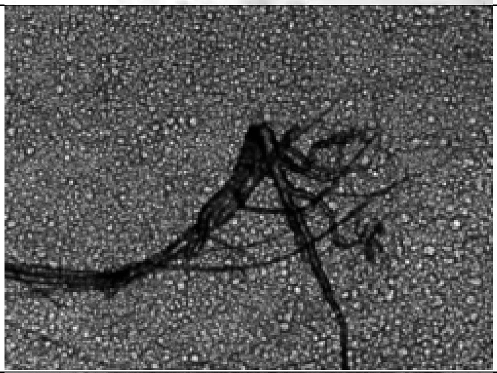	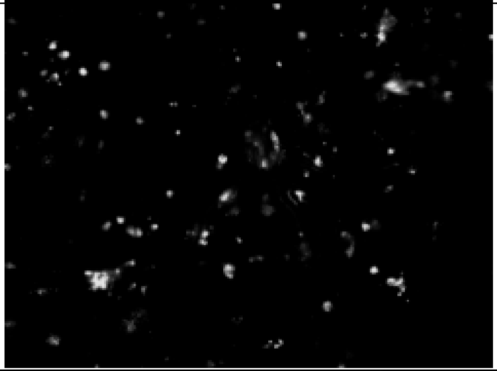	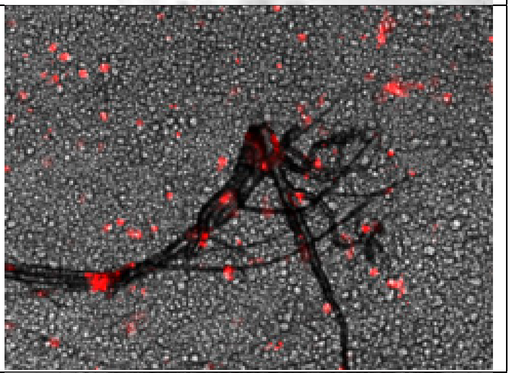
Fat body / hemocytes and / 1 HPE	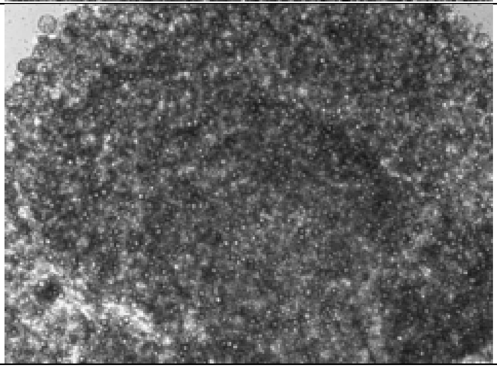	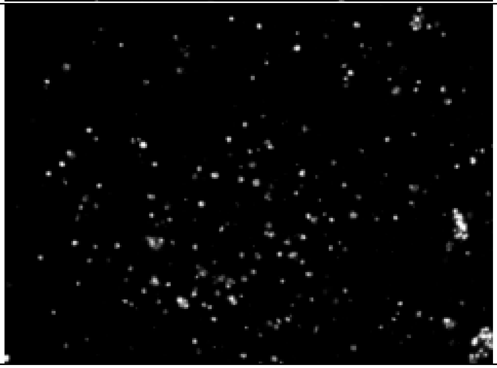	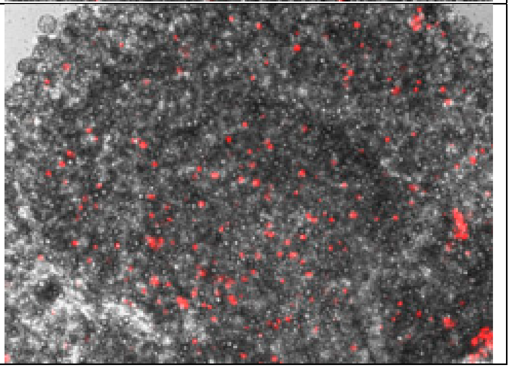

Malpighian tubules /lack of hemocytes / 1 HPE	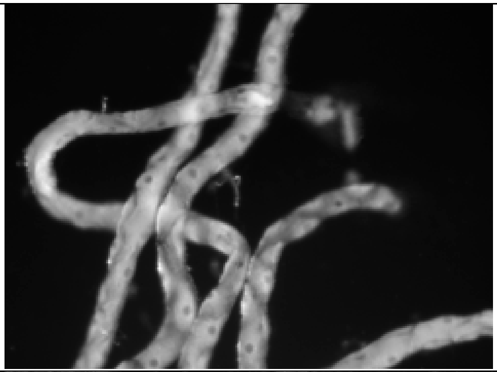	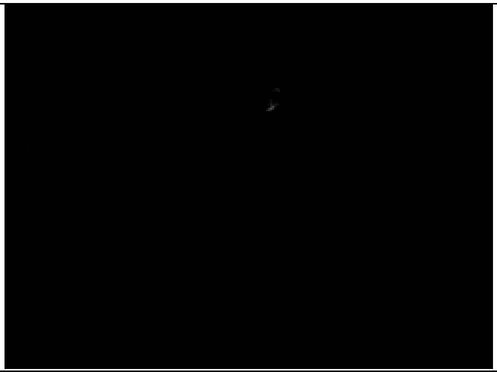	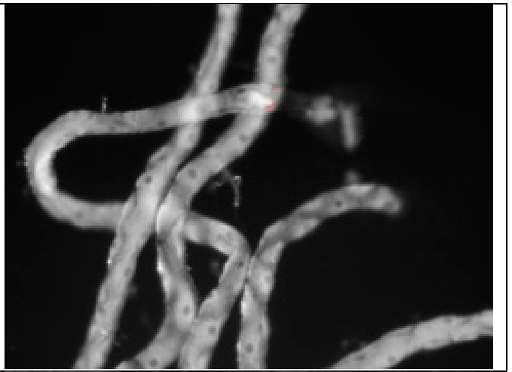
Alimentary tract with no signal and hemocytes / 1 HPE	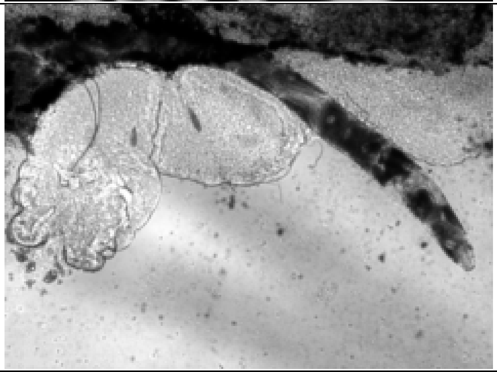	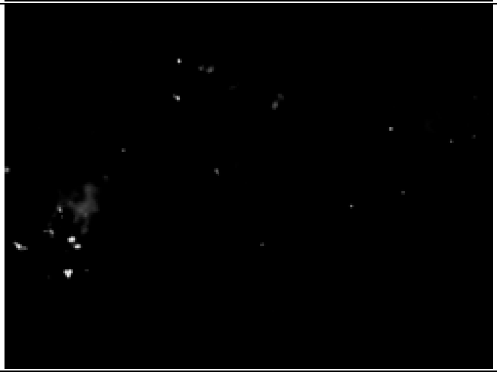	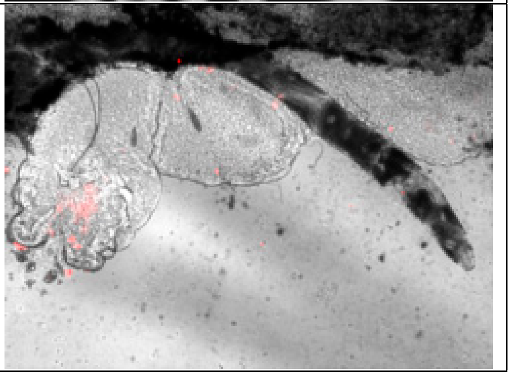
Midgut with no signal and coagulated blood/meal / 24 HPE	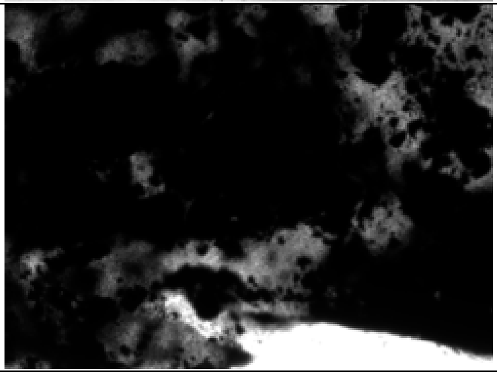	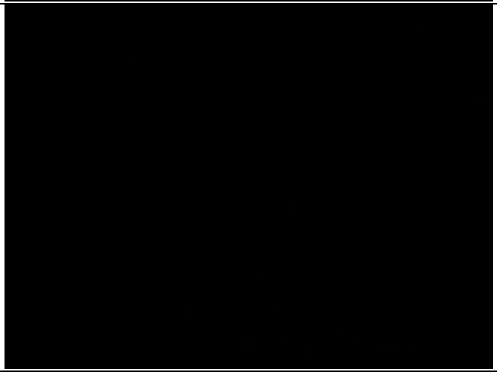	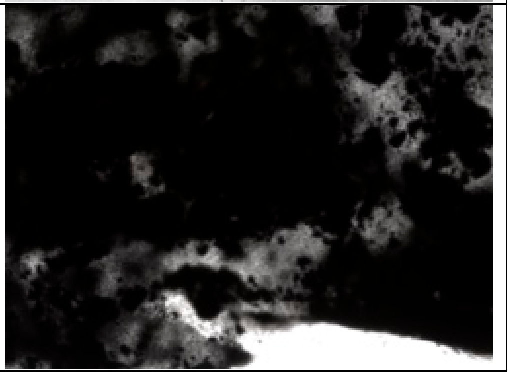

Hindgut / no signal and pericardial cells in abdomen / 24 HPE	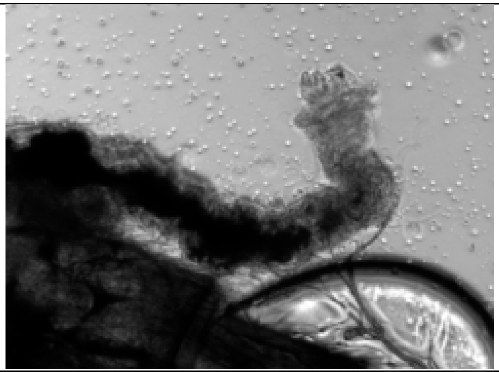	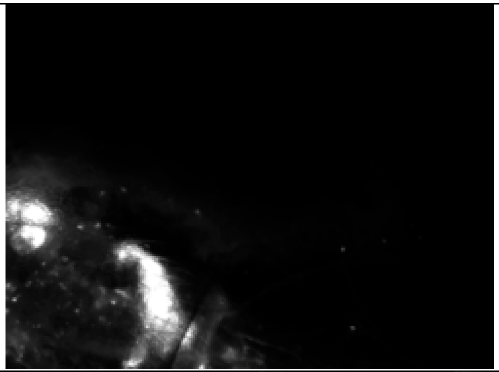	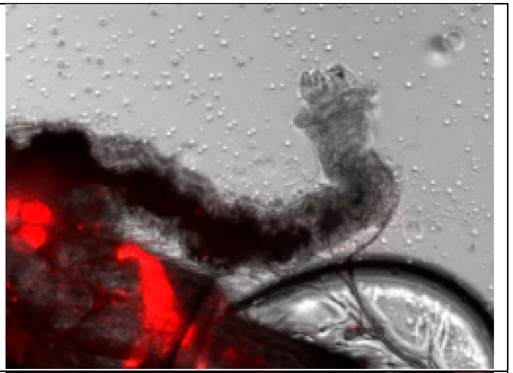
Ovary / fluorescence in oocytes / 1 HPE	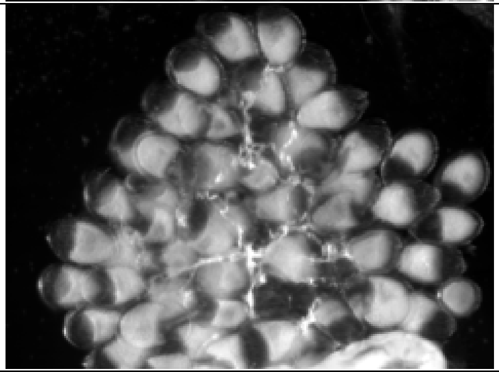	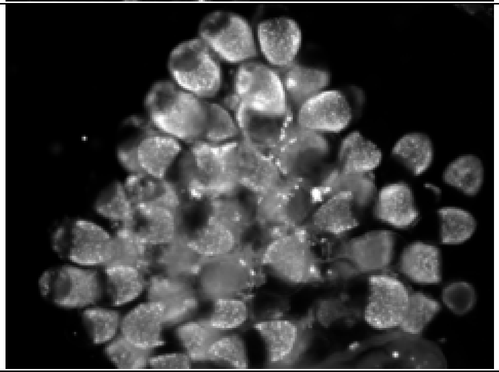	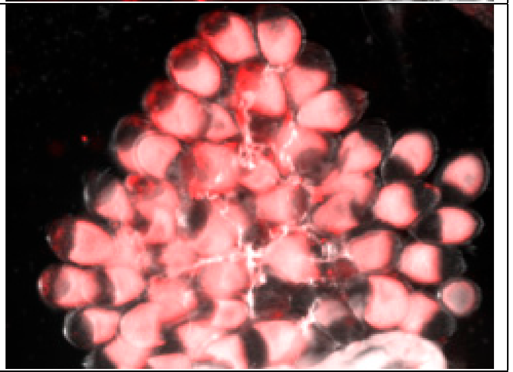
Ovary / fluorescence in atretic and healthy follicles / 24 HPE	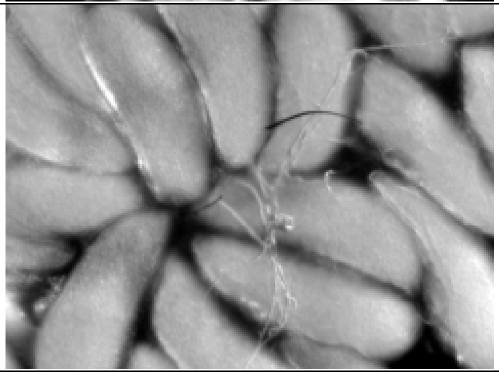	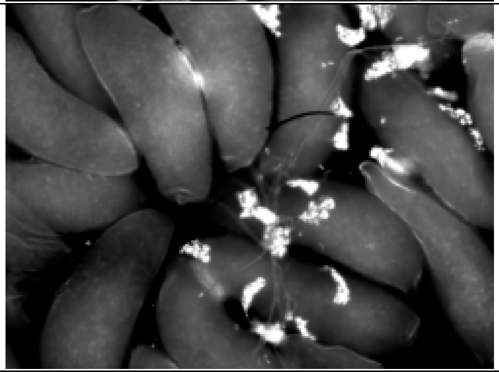	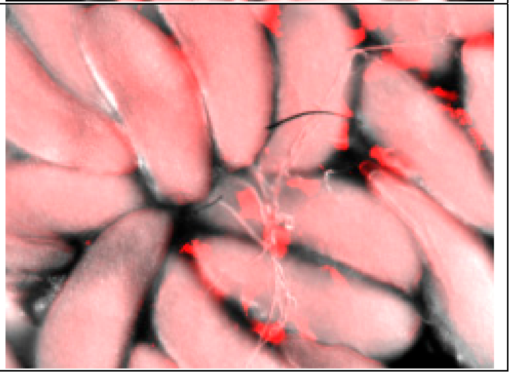

Figure S6 – Adult female *Cx. pipiens* 25-48 hours post blood/meal - intrathoracic injection

Tissue	Bright Field	RNAi trigger	Merge
Head / injection size and hemocytes / 24 HPE	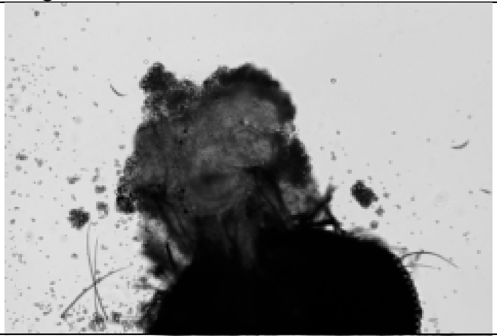	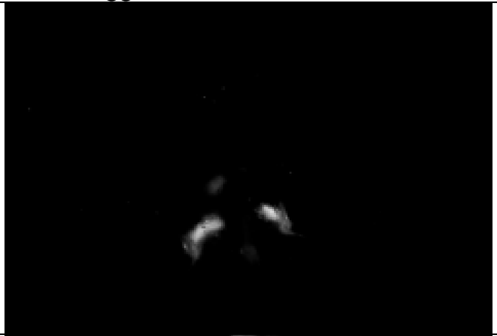	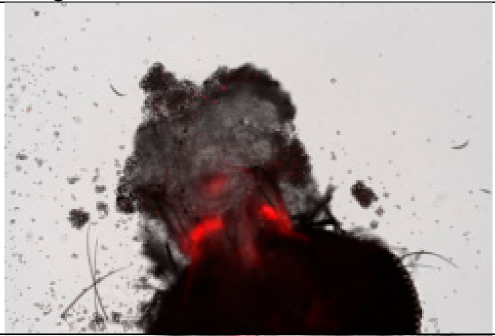
Abdomen / pericardial cells / 24 HPE	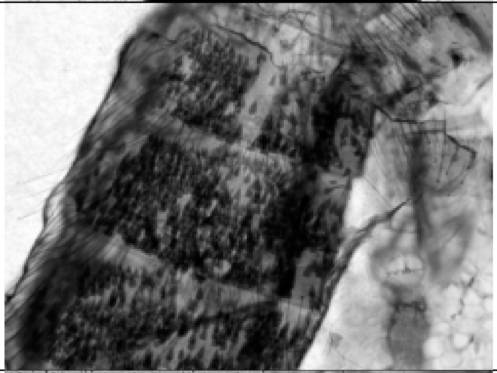	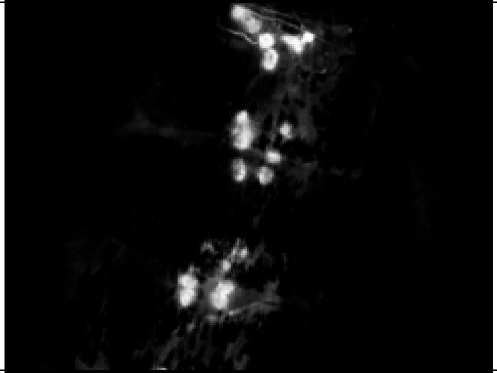	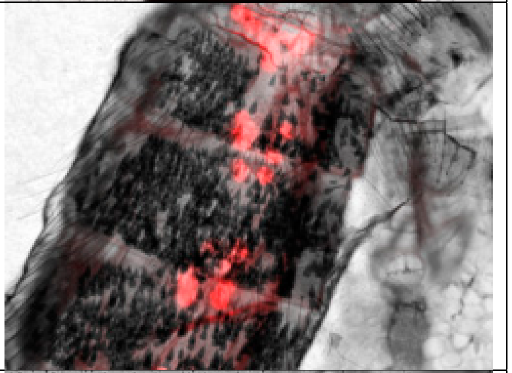
Leg / hemocytes / 1 HPE	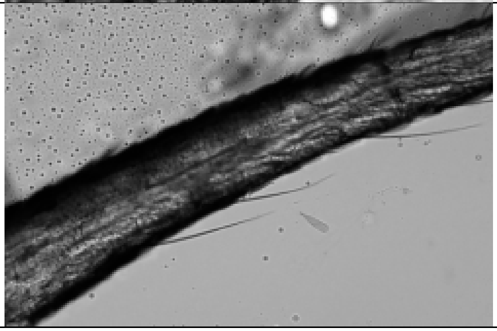	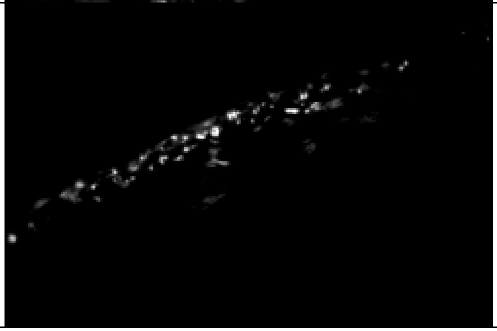	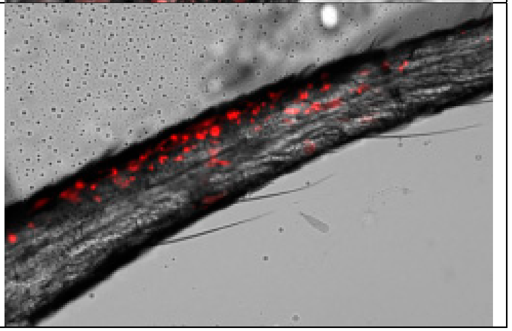

Fat body /fat body and hemocytes / 24 HPE	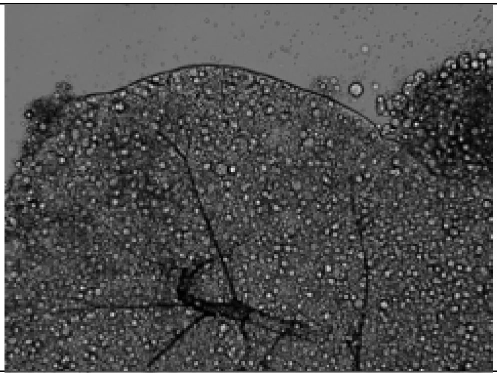	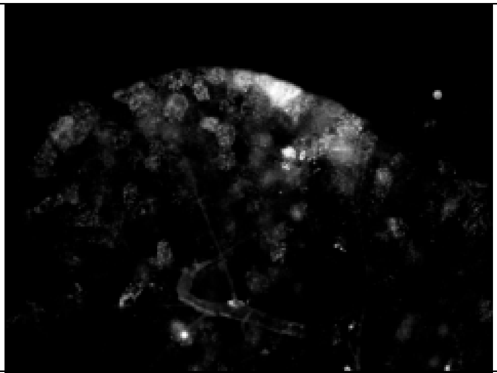	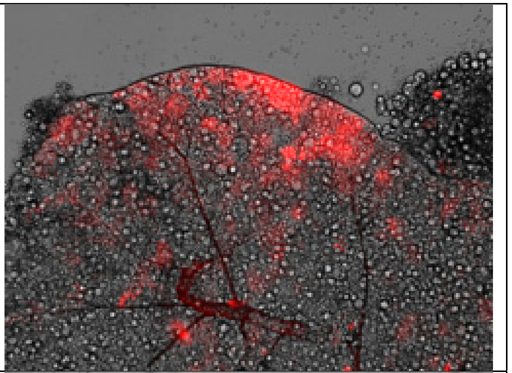
Fat body /fat body / 24 HPE	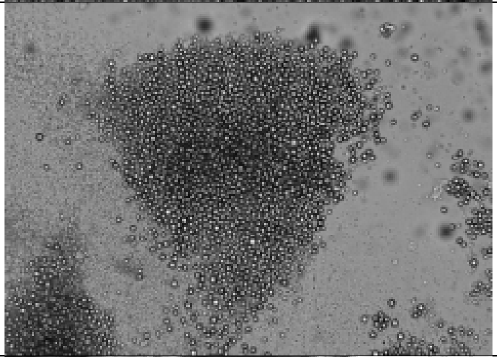	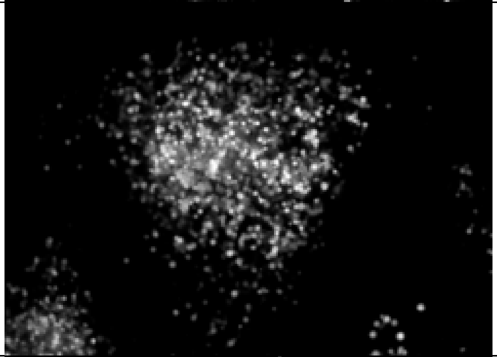	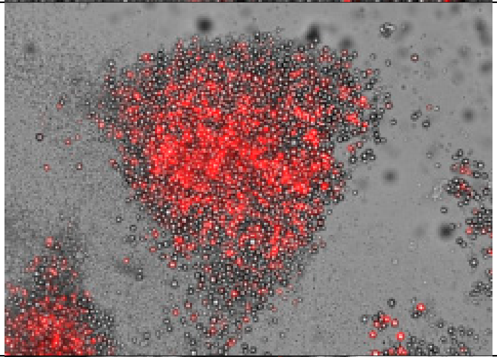
Fat body / hemocytes associated with tracheoles / 24 HPE	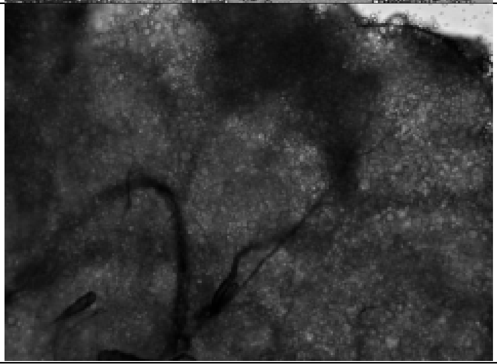	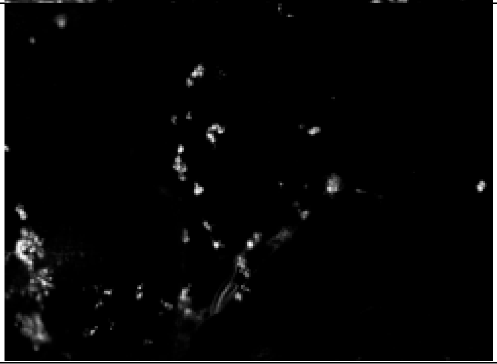	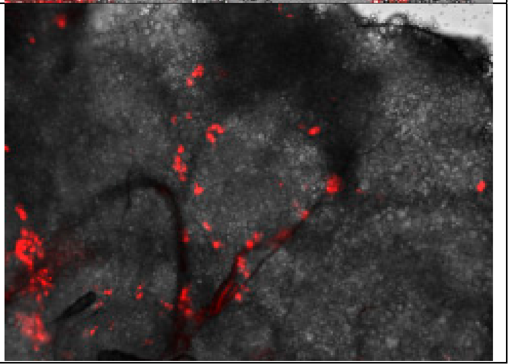

Malpighian tubules /hemocytes associated with tracheole / 1 HPE	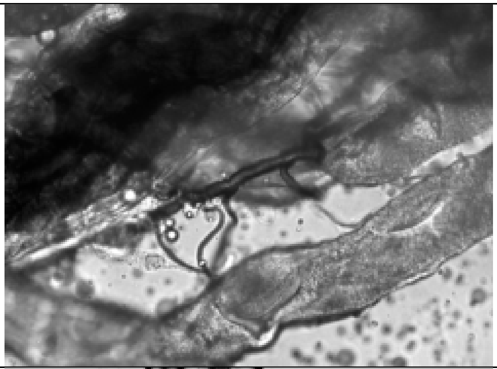	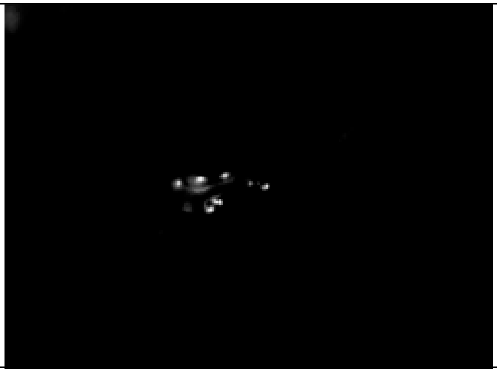	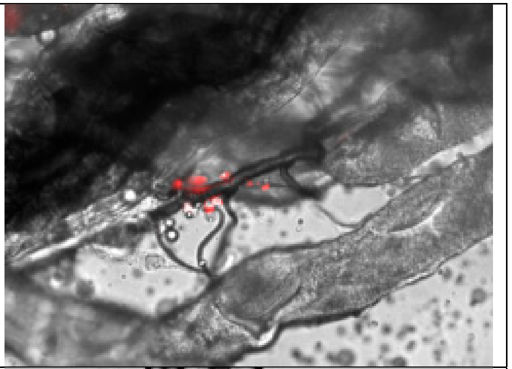
Alimentary tract with auto-fluorescent signal / 1 HPE	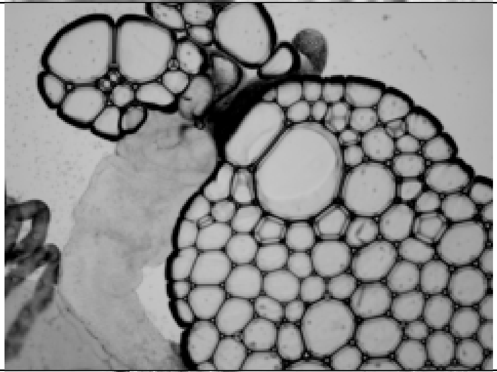	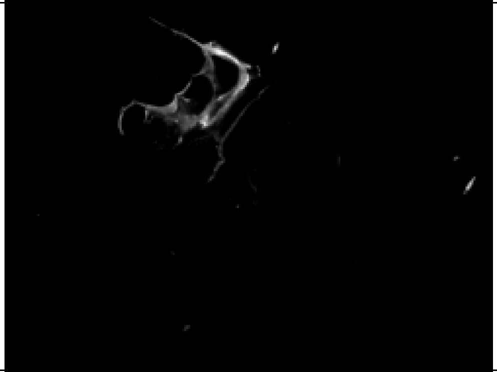	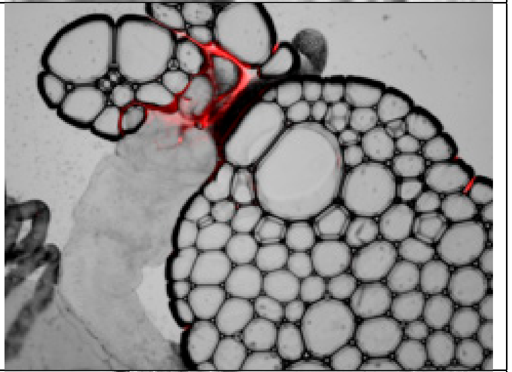
Midgut with punctate auto-fluorescent signal / 1 HPE	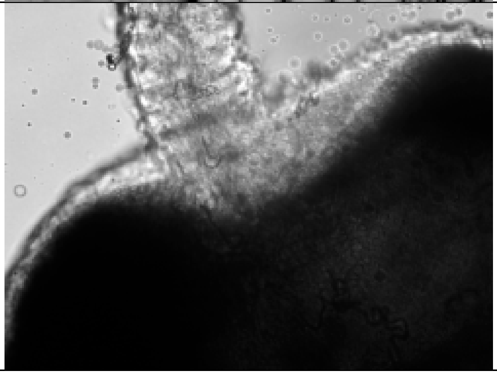	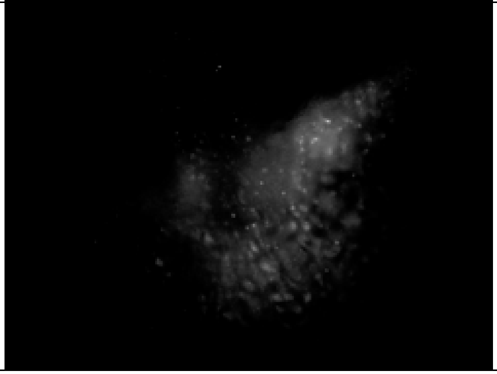	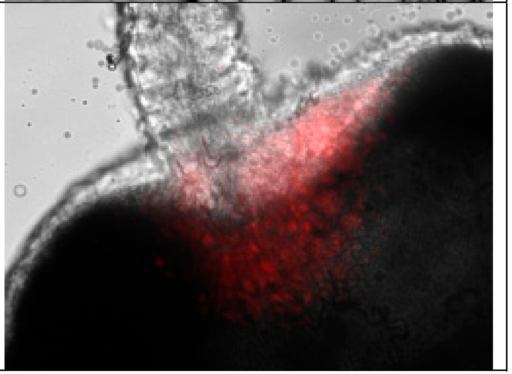

Ovary / fluorescence in oocytes / 1 HPE	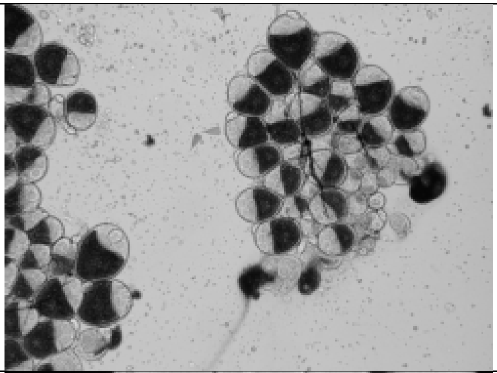	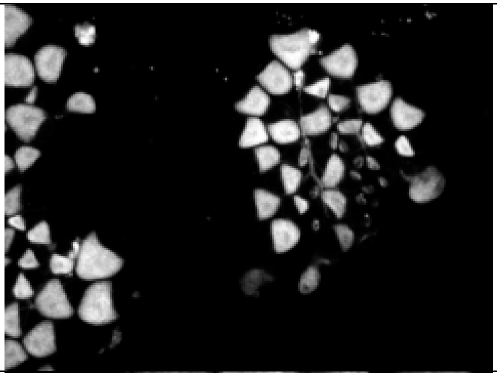	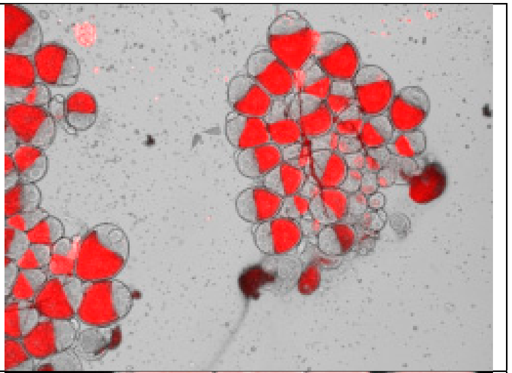
Ovary / fluorescence in oocytes / 24 HPE	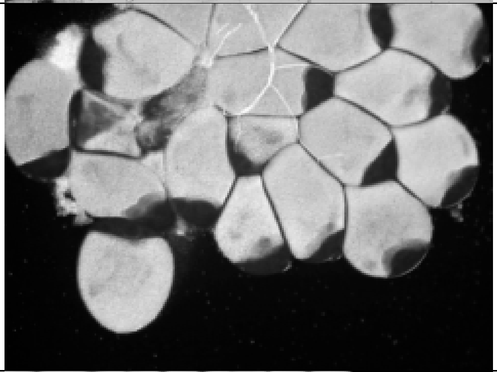	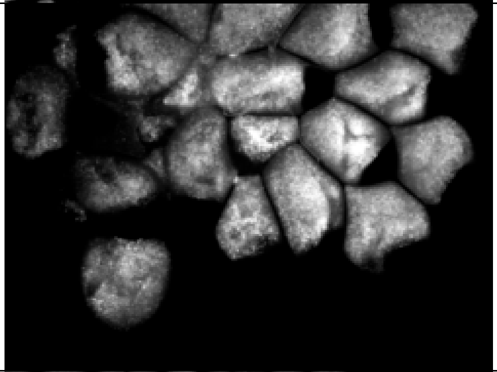	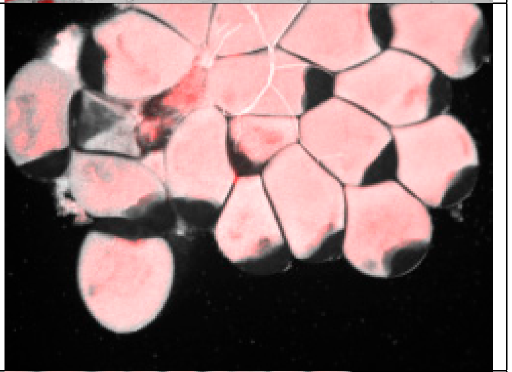
Ovary / fluorescence in healthy and atretic follicles / 24 HPE	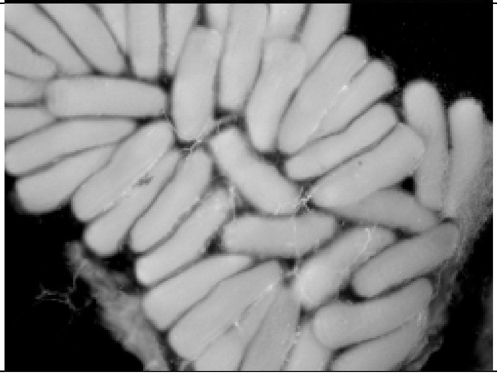	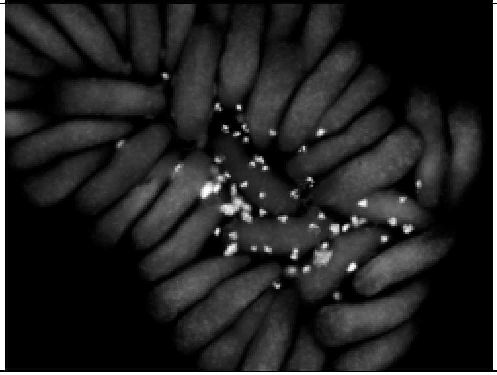	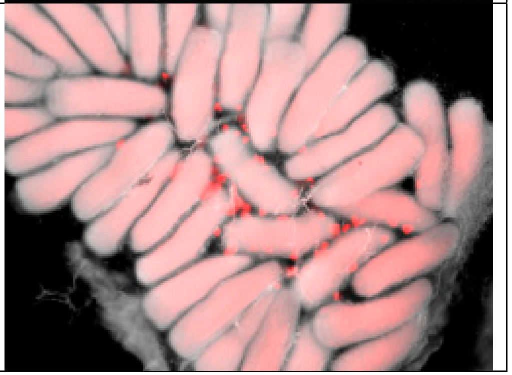

Figure S7 – 4th instar *Ae. Aegypti* - intrathoracic injection

Tissue	Bright Field	RNAi trigger	Merge
Head / 24 HPE	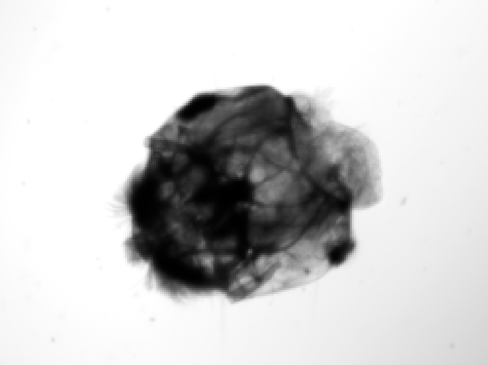	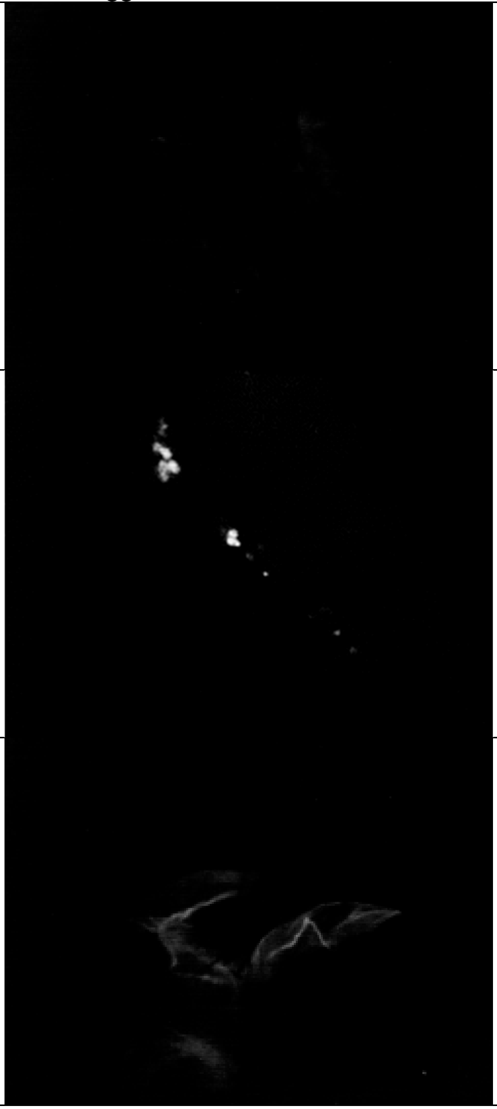	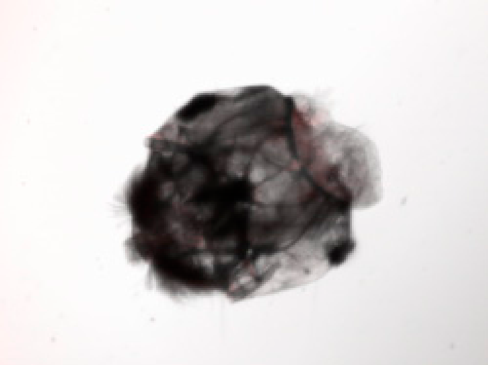
Abdomen / pericardial cells / 24 HPE	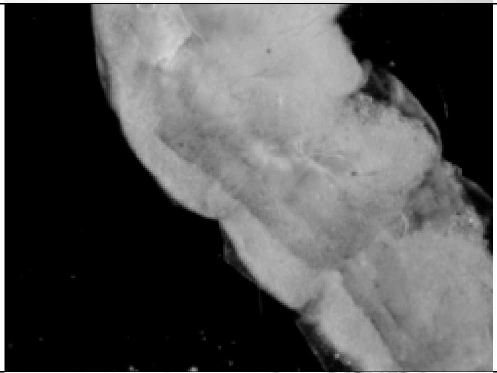		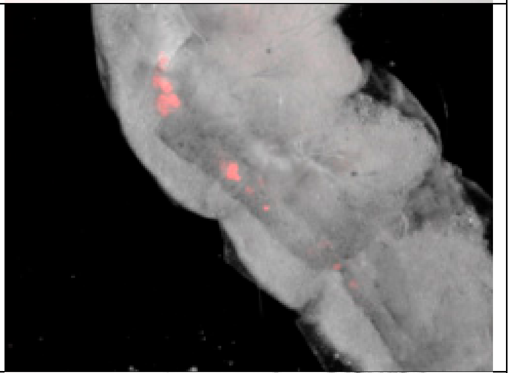
Abdomen / terminal segment / auto-fluorescence in cuticle / 24 HPE	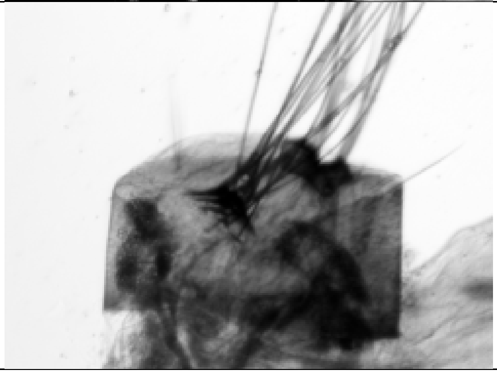		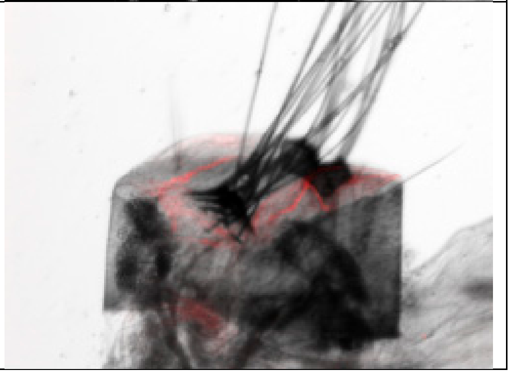

Abdomen / Terminal segment /respiratory siphon	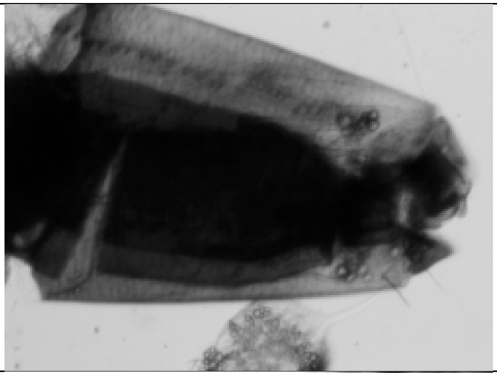	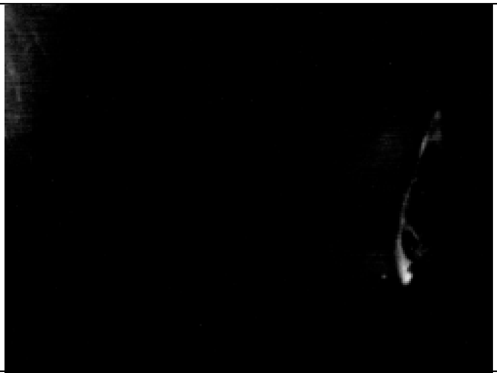	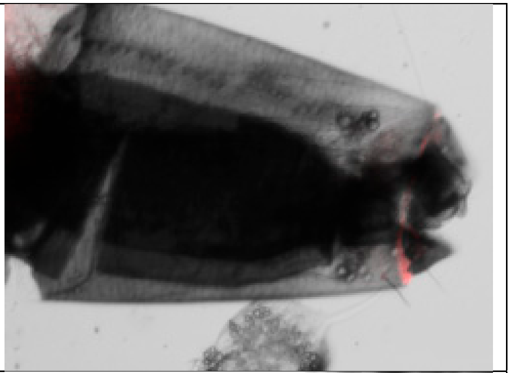
Caecum / 24 HPE	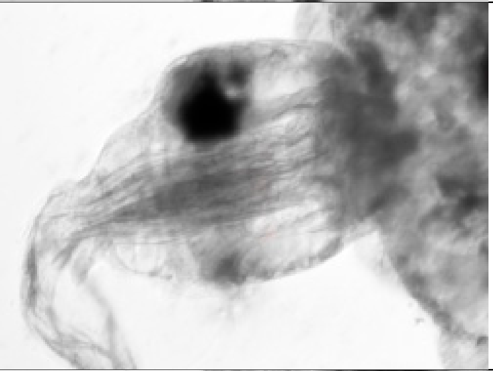	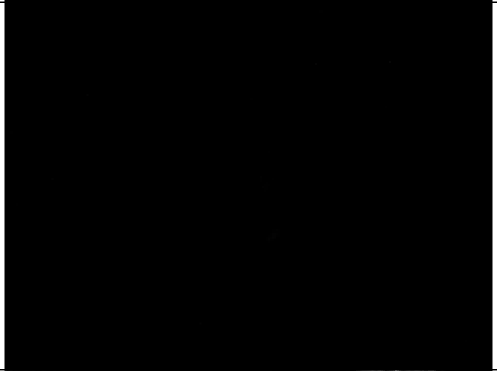	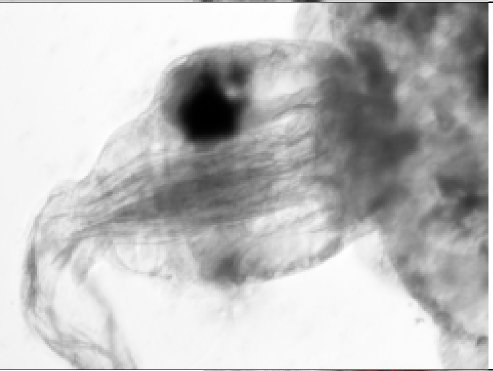
Alimentary tract (midgut) with extracellular signal / 24 HPE	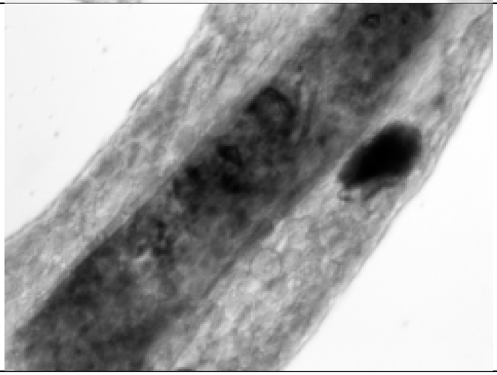	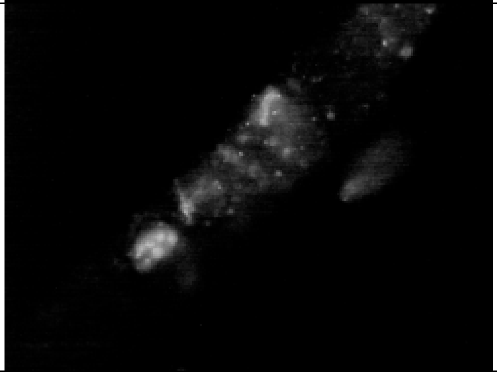	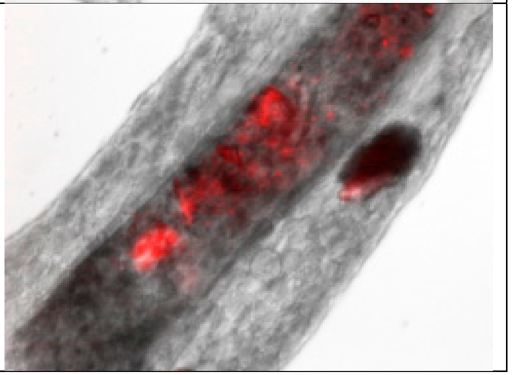

Malpighian tubules / 24 HPE	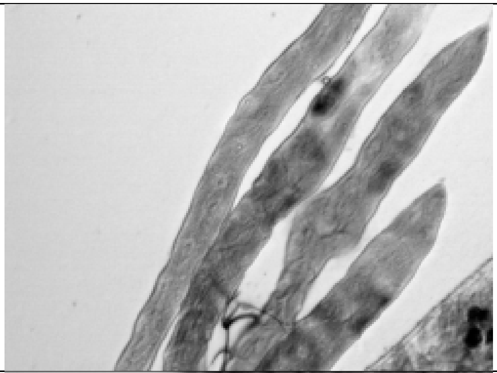	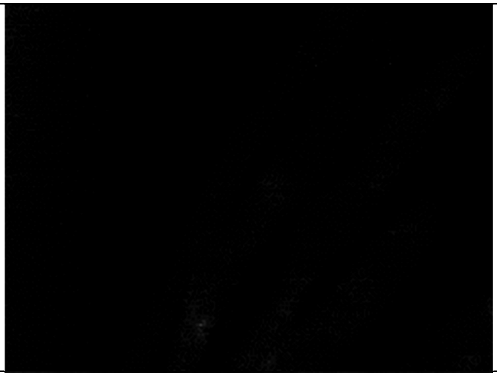	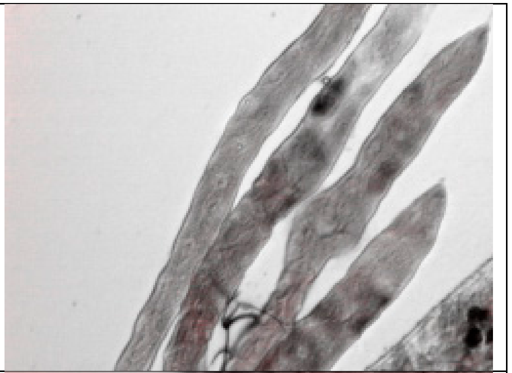
Alimentary tract (anal canal / hindgut) with no signal / 24 HPE	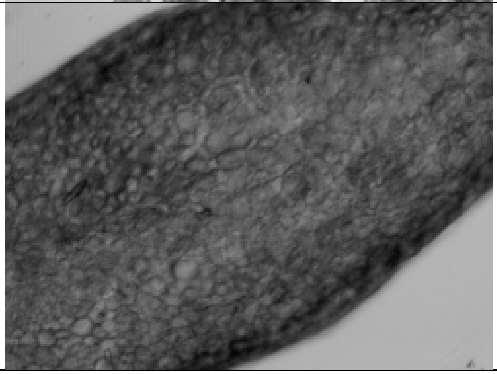	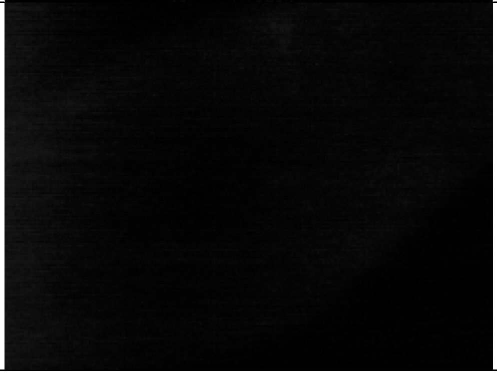	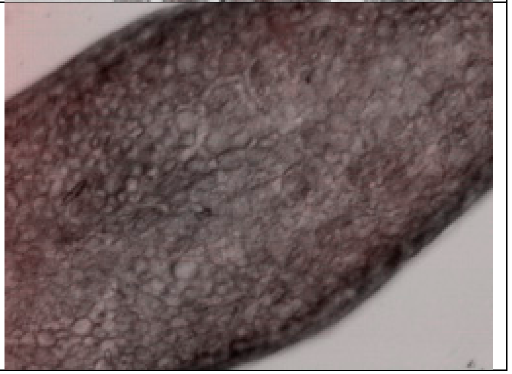

Figure S8 – 4th instar *An. gambiae* – intrathoracic injection

Tissue	Bright Field	RNAi trigger	Merge
Head / hemocytes / 24 HPE	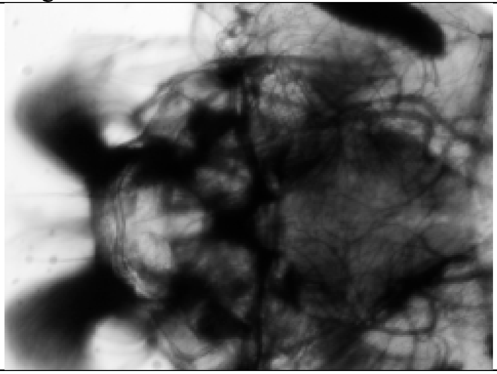	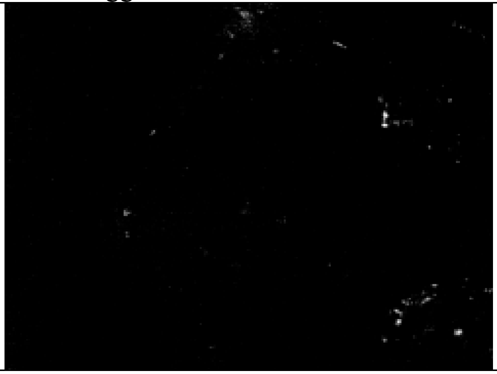	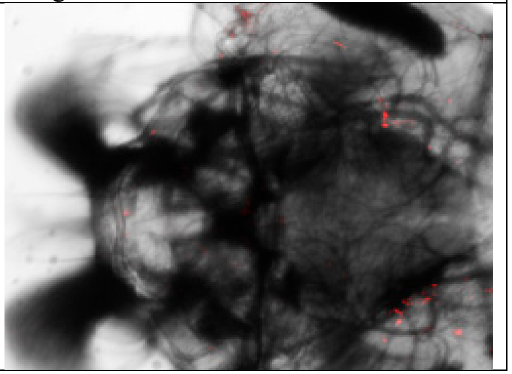

Abdomen / pericardial cells and hemocytes (see enhanced image below) / 24 HPE	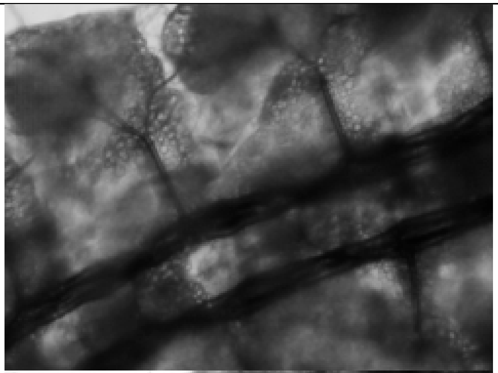	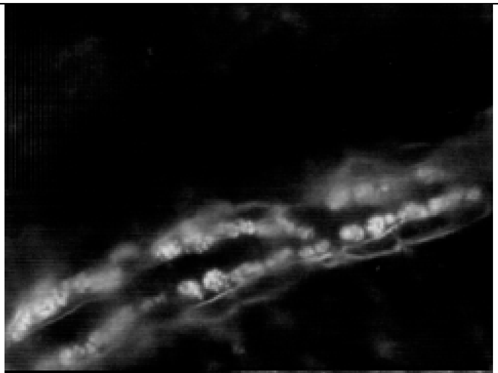	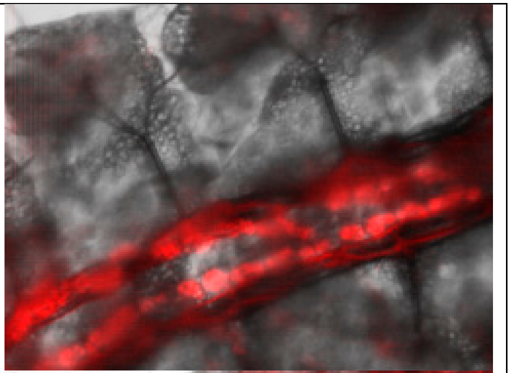
	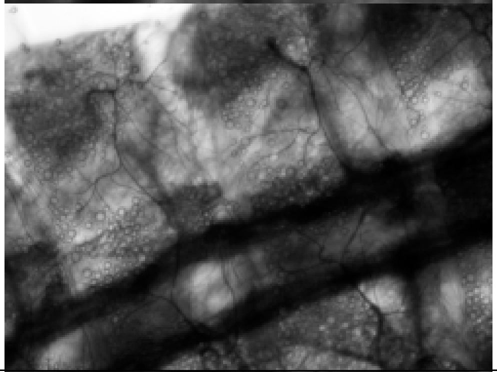	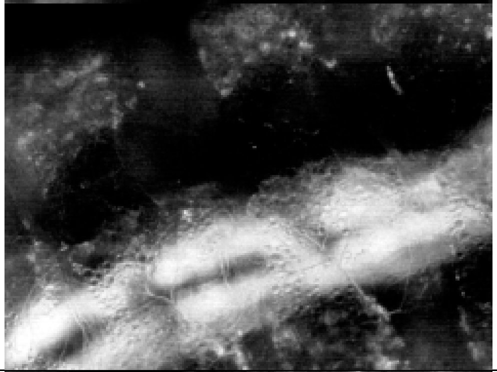	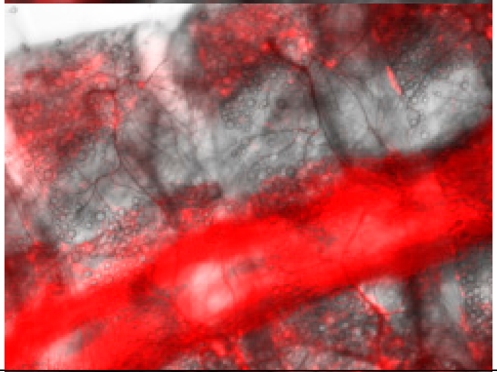
Abdomen / pericardial cells (single surviving adult at 120 HPE	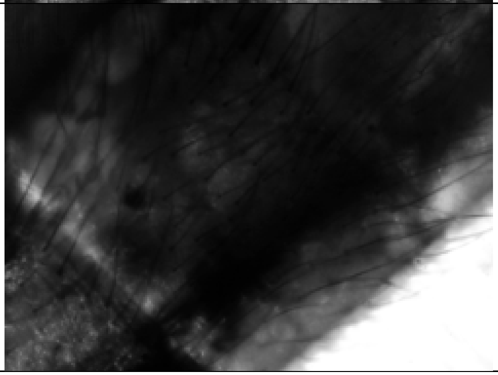	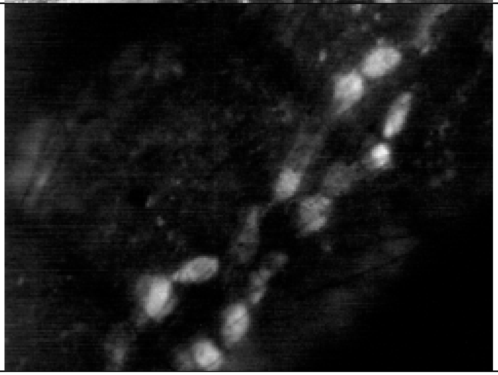	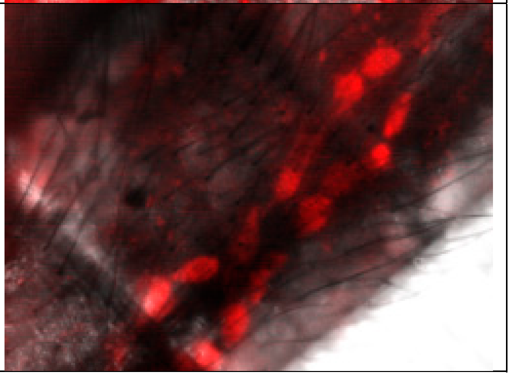

Abdomen / terminal segment / hemocytes / 24 HPE	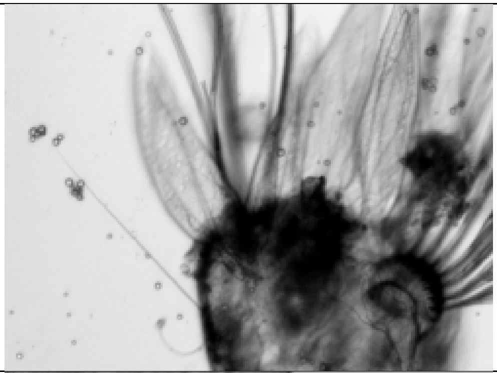	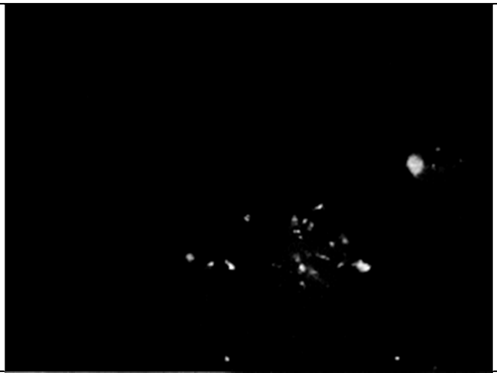	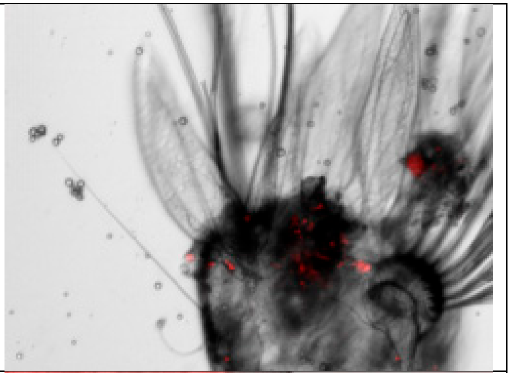
Alimentary tract (midgut and Malpighian tubule) with extracellular signal / 24 HPE	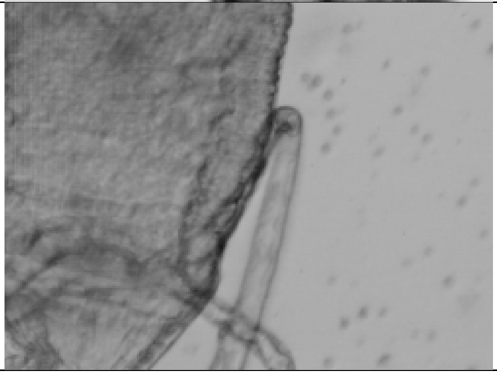	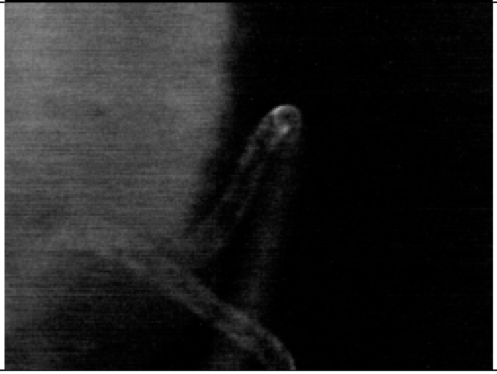	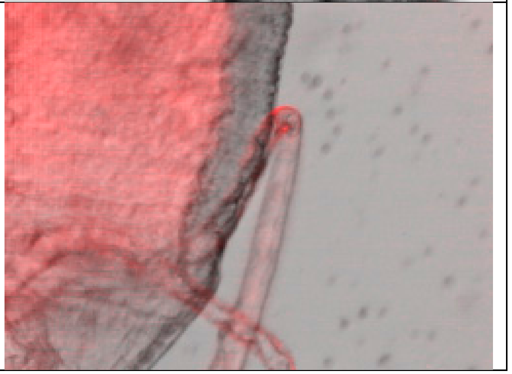

Figure S9 – 4th instar *Cx. pipiens* – intrathoracic injection

Tissue	Bright Field	RNAi trigger	Merge

Head / 24 HPE	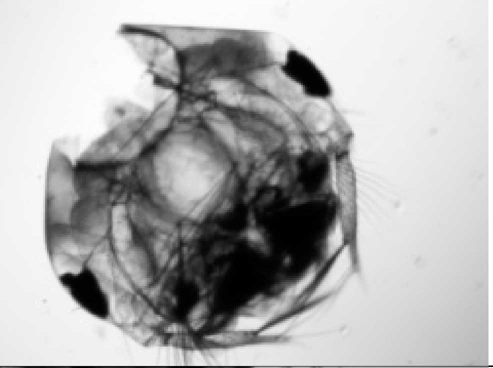	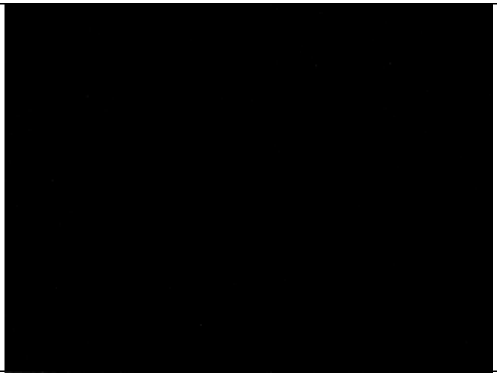	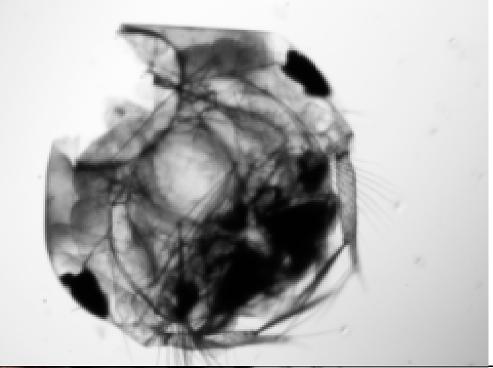
Abdomen / pericardial cells / 24 HPE	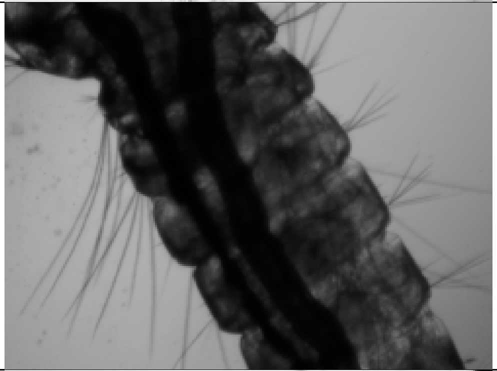	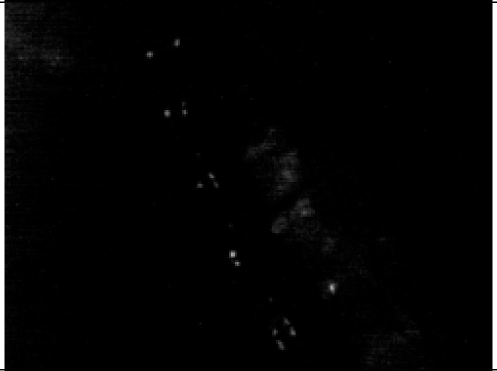	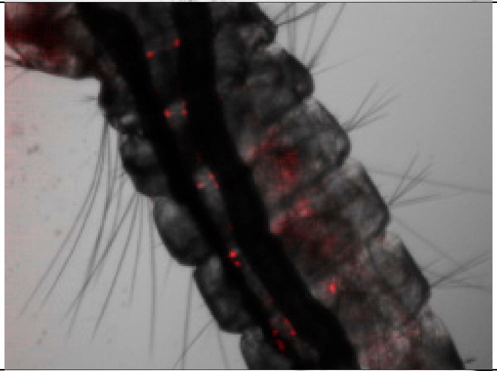
Abdomen / pericardial cells in dissected tissue / 24 HPE	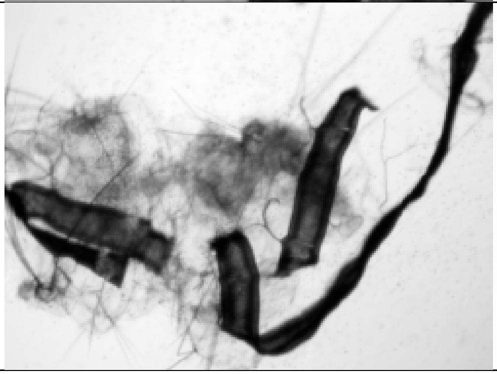	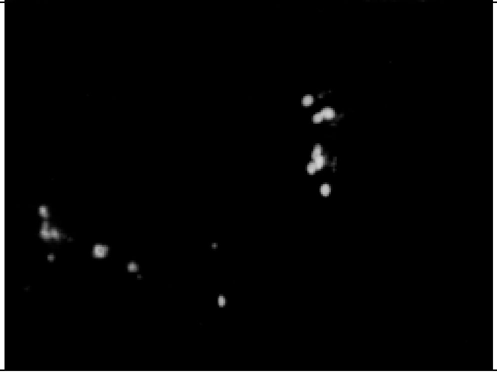	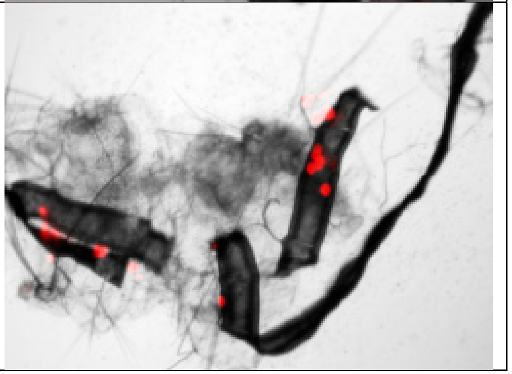

Abdomen / Terminal segment / respiratory siphon, Malpighian tubules and anal canal / 24 HPE	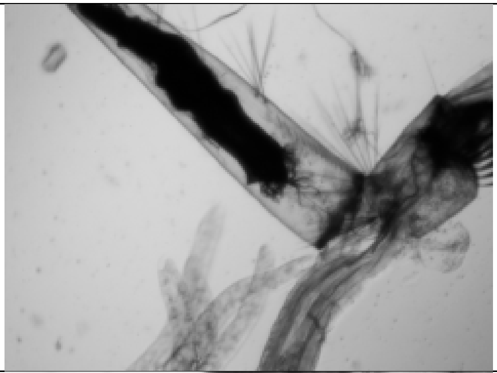	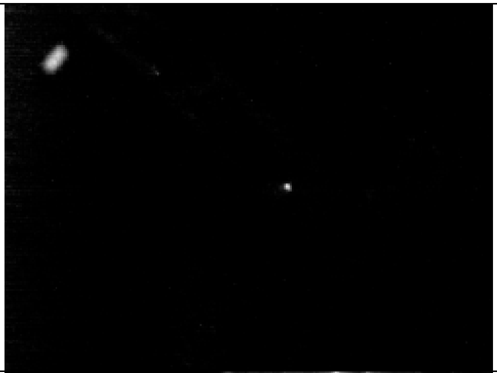	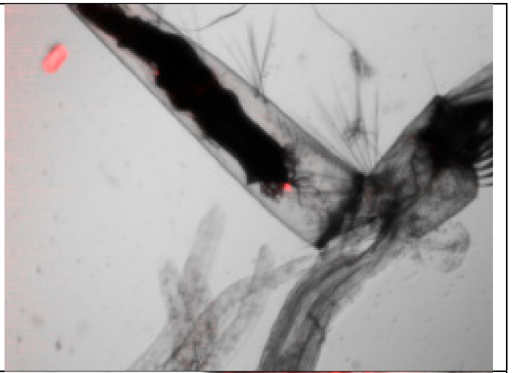
Abdomen / pericardial cells and hemocytes and auto-fluorescence / 24 HPE	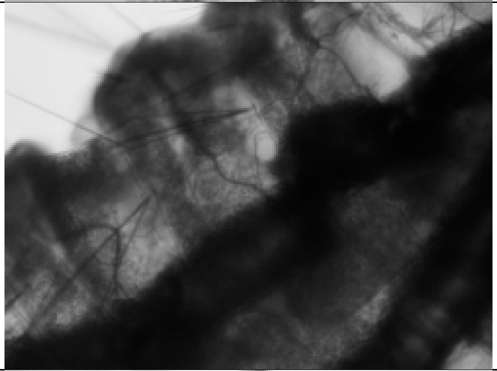	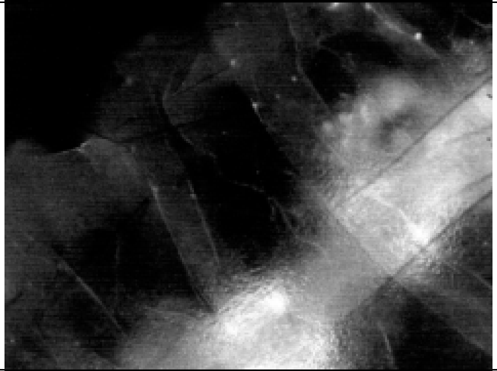	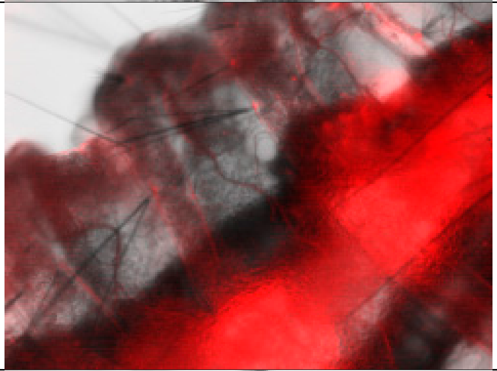
Caecum / auto-fluorescence / 24 HPE	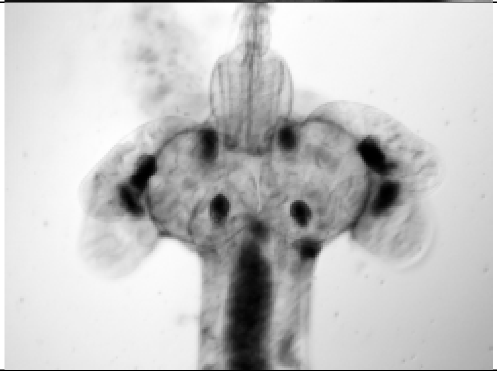	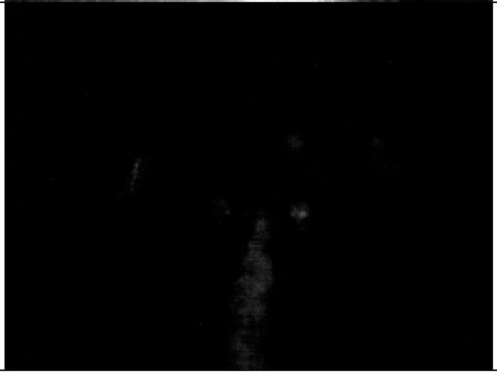	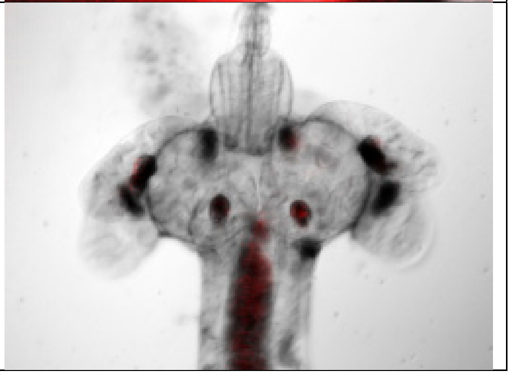

Alimentary tract (midgut) with extracellular signal / 24 HPE	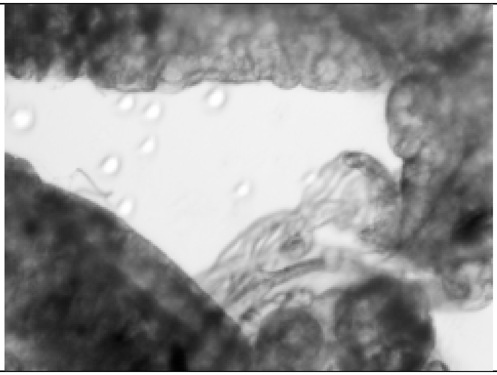	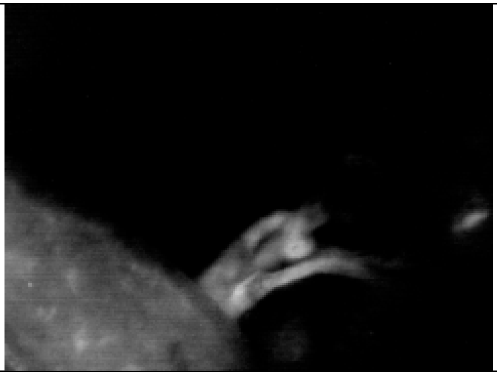	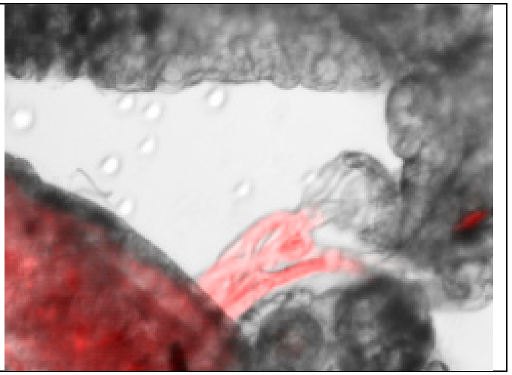

Adult exposure by Topical Application

Figure S10 – Adult female *Ae. aegypti* – topical exposure

Tissue	Bright Field	RNAi trigger	Merge
Head	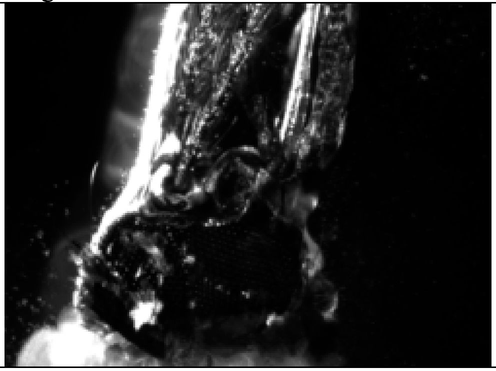	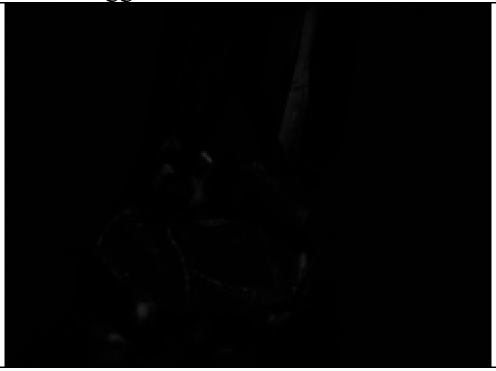	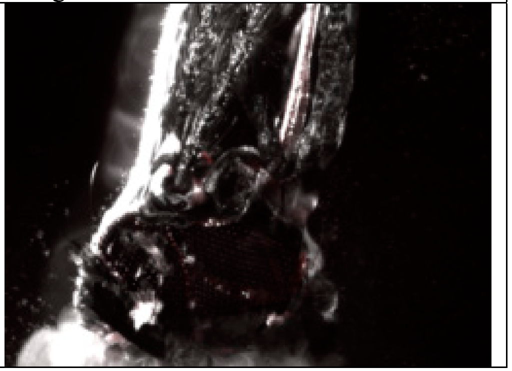

Abdomen / signal on cuticle	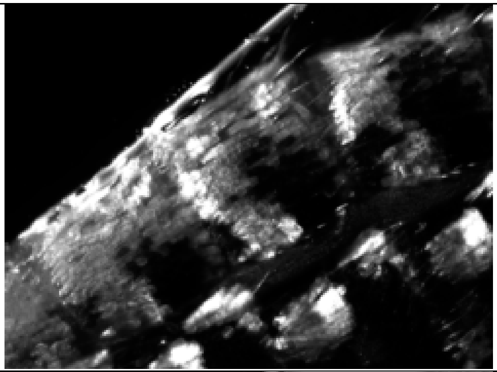	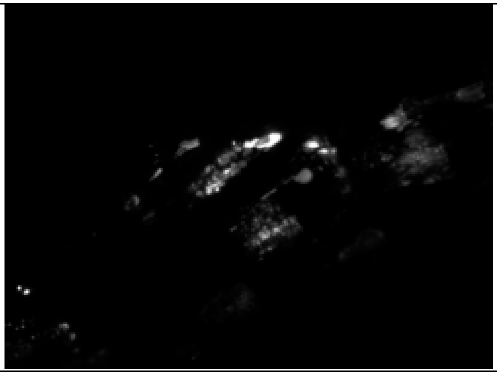	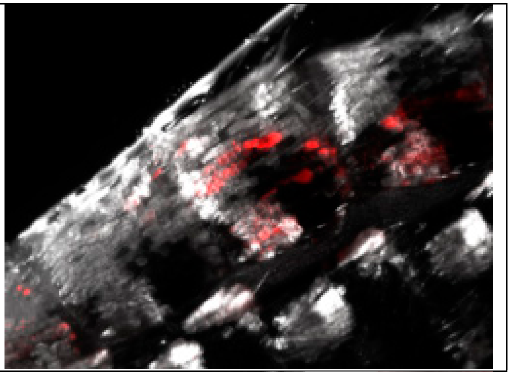
Leg / signal on cuticle	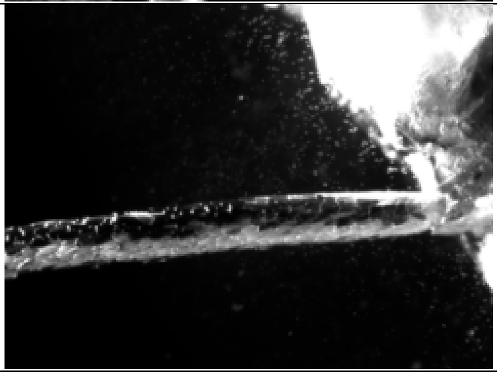	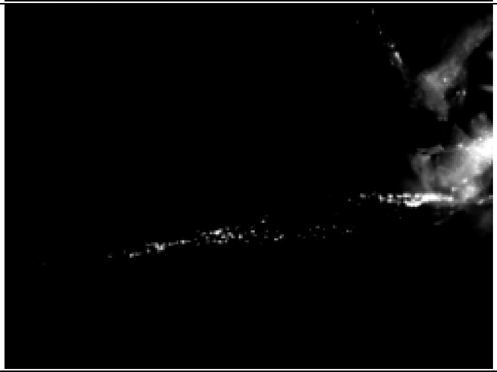	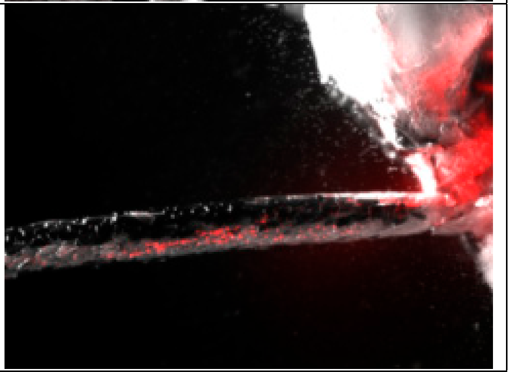
Leg / no signal	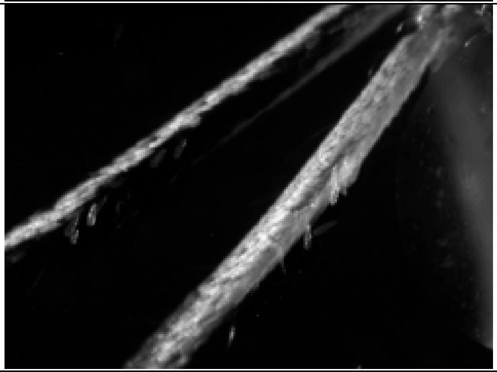	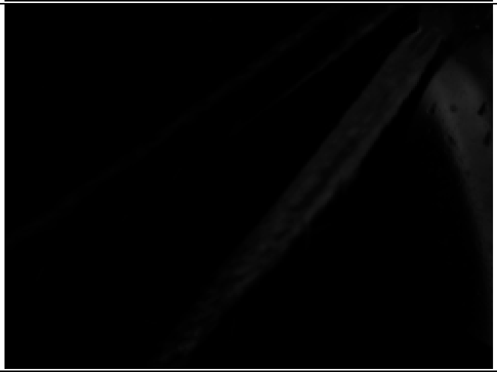	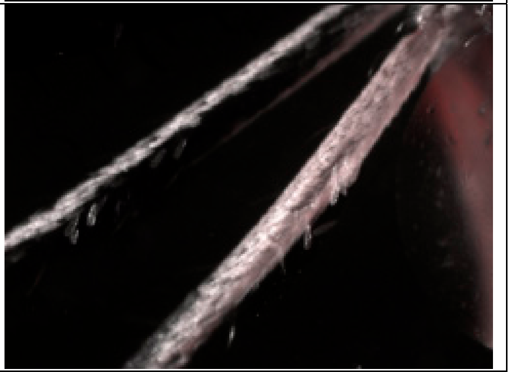

Alimentary tract (midgut and Malpighian tubules) with no signal	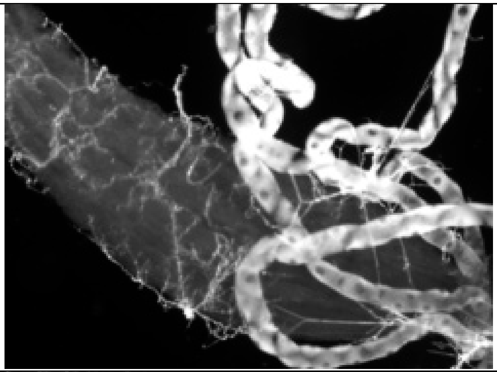	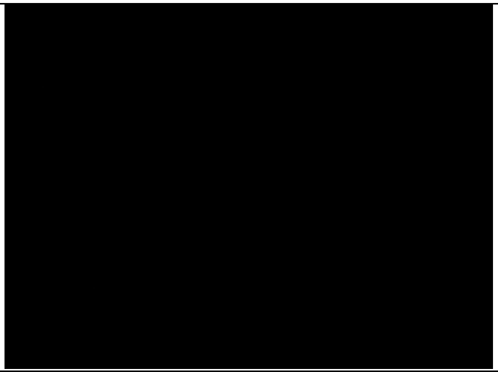	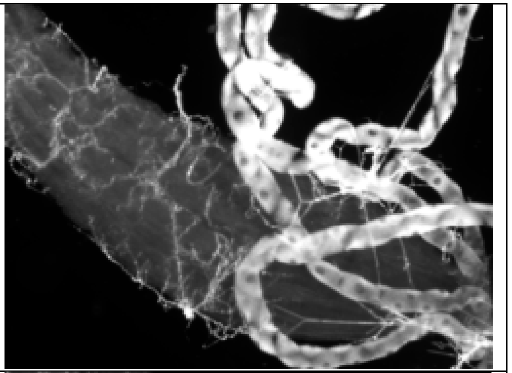
Alimentary tract (midgut and Malpighian tubules) with no signal	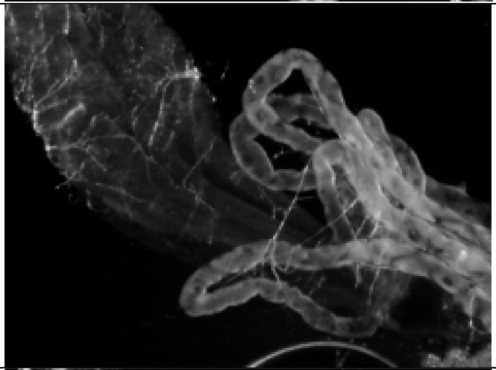	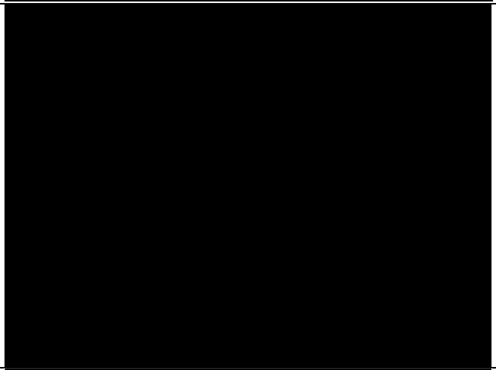	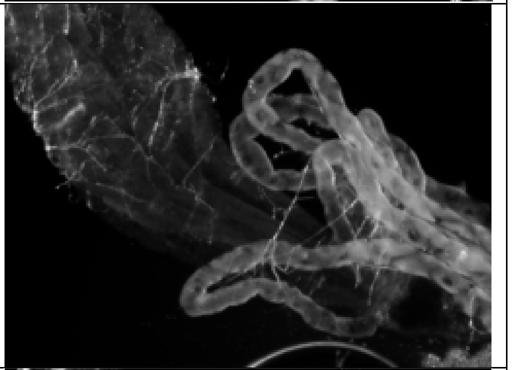
Alimentary tract (hindgut and Malpighian tubules) and ovaries with no signal	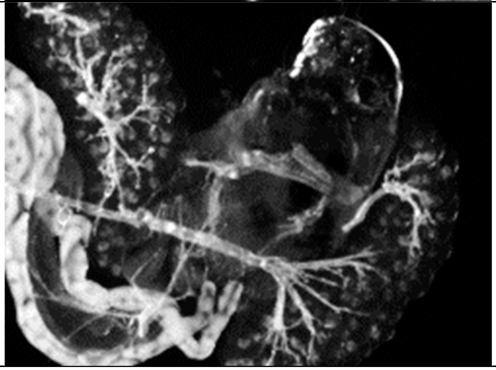	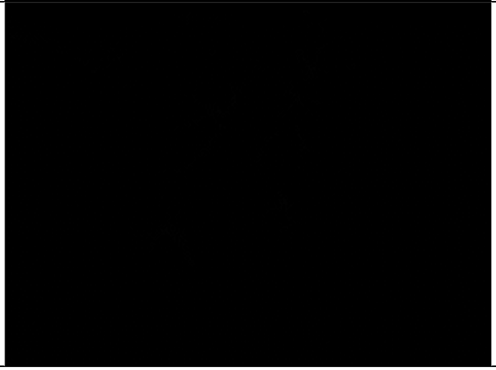	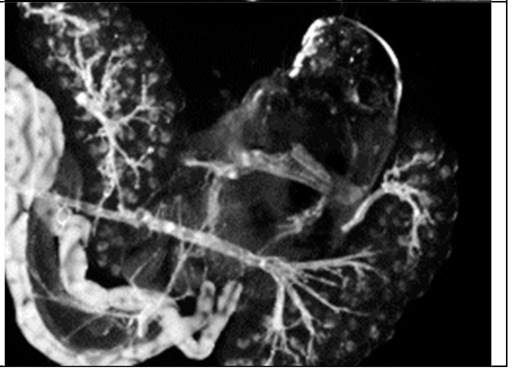

Ovary	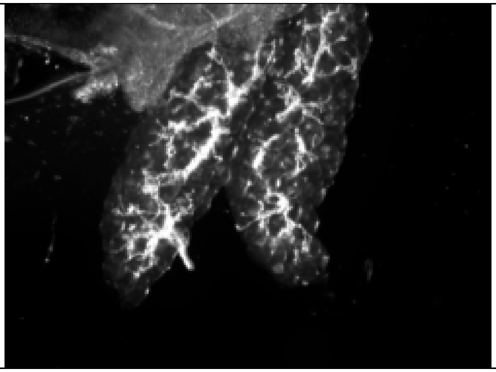	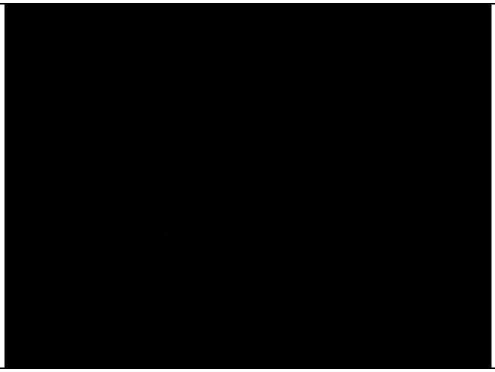	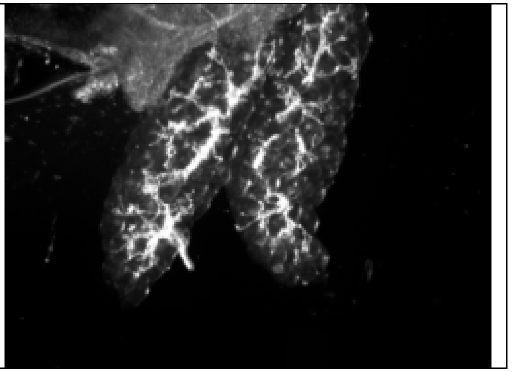

Figure S11 – Adult female *An. gambiae* – topical exposure

Tissue	Bright Field	RNAi trigger	Merge
Head / auto-fluorescence	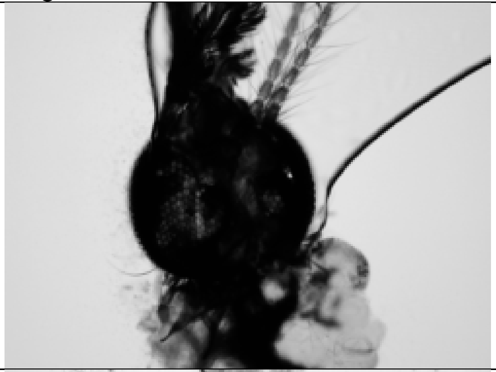	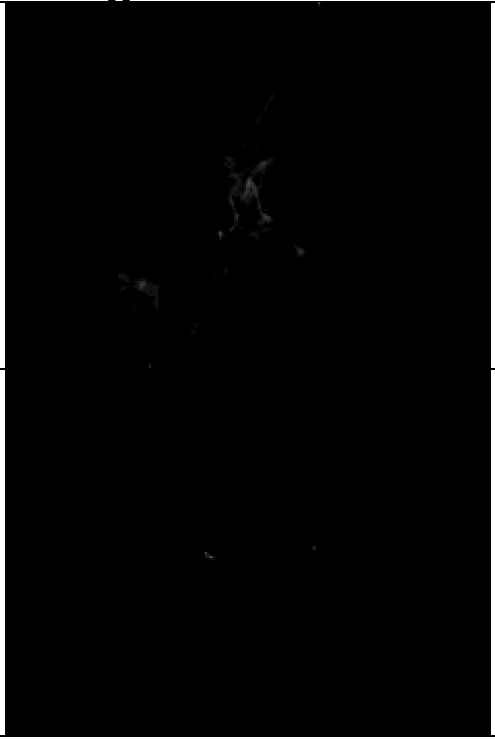	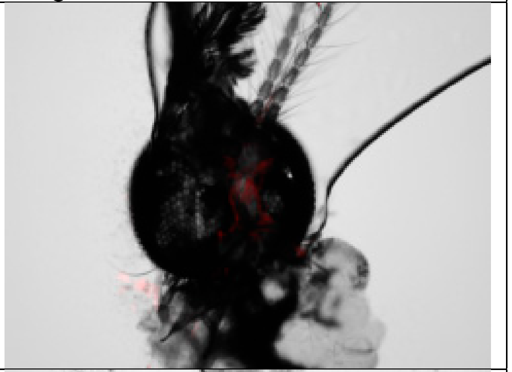
Abdomen / no signal	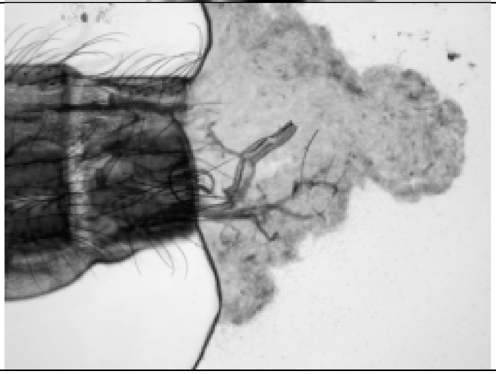		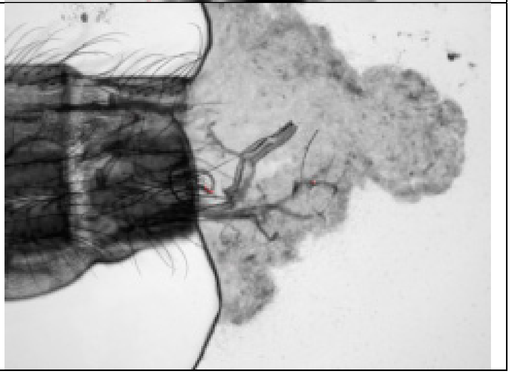

Abdomen / signal on cuticle	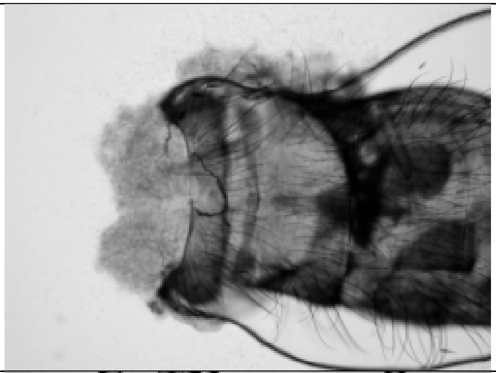	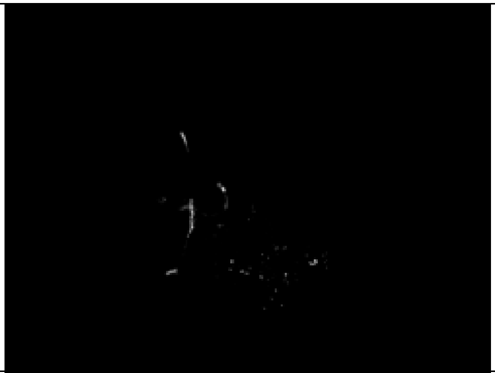	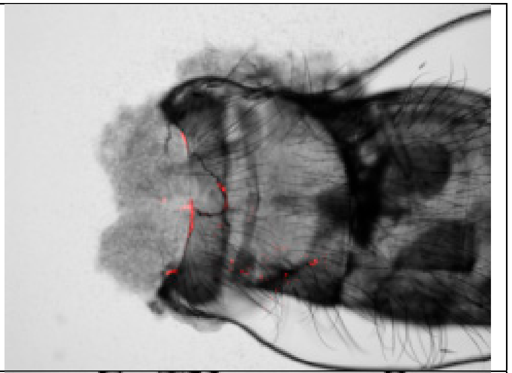
Alimentary tract (midgut and Malpighian tubules) no signal	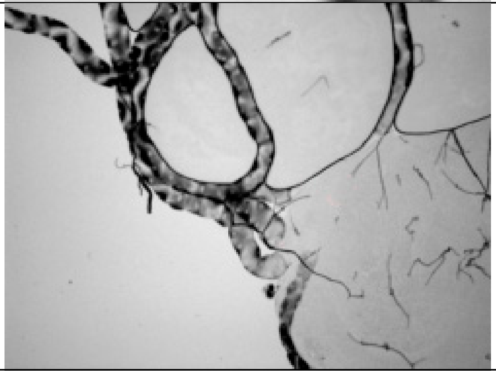	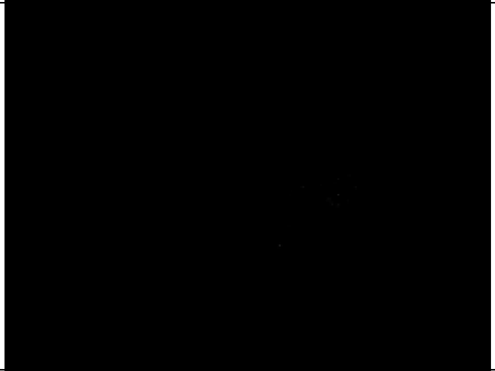	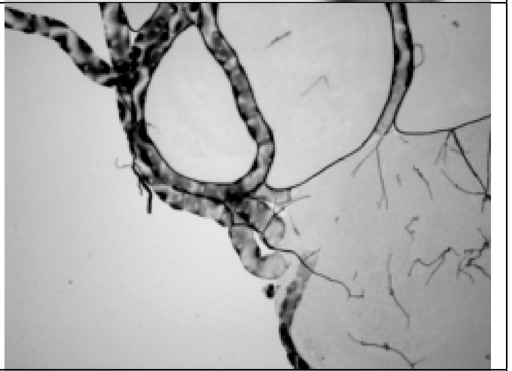
Alimentary tract (foregut, midgut and Malpighian tubules) with no signal	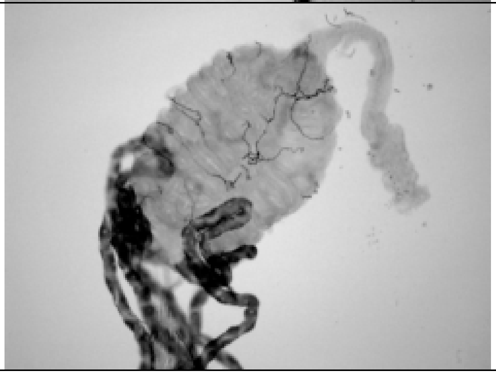	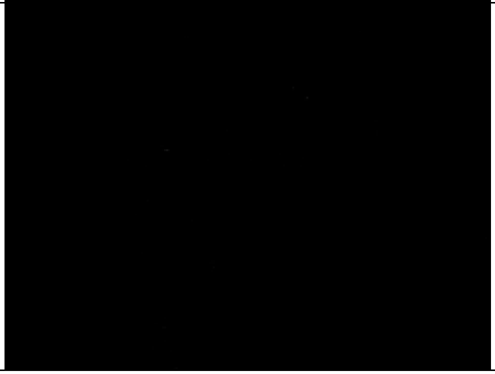	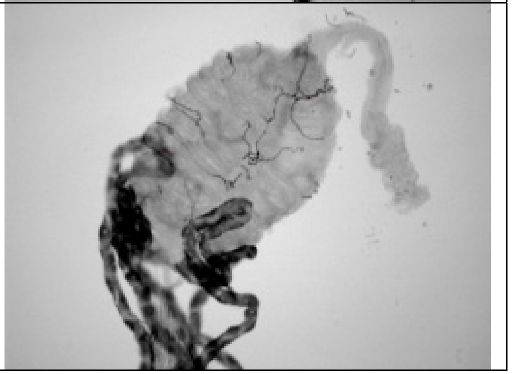

Alimentary tract (midgut and Malpighian tubules) and ovary with no signal	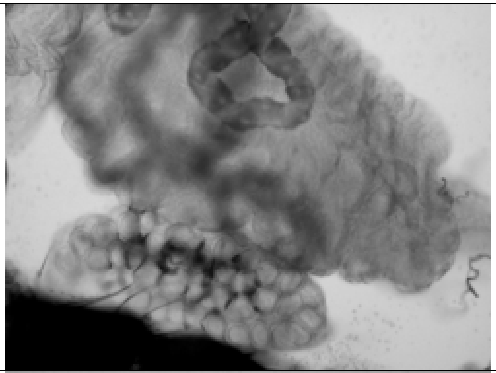	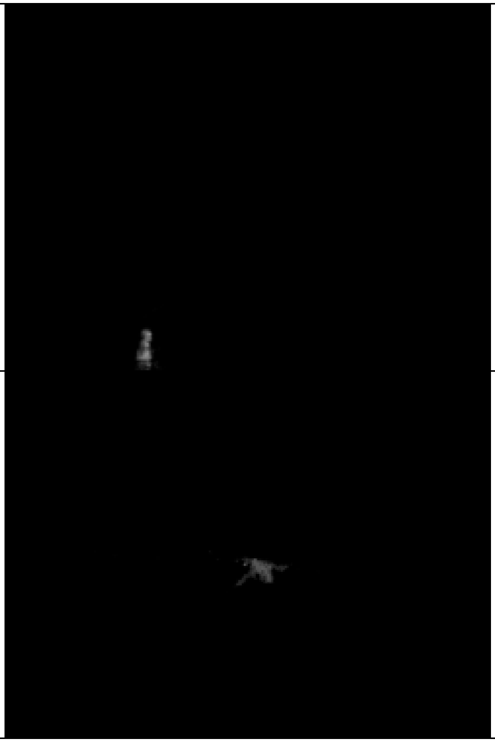	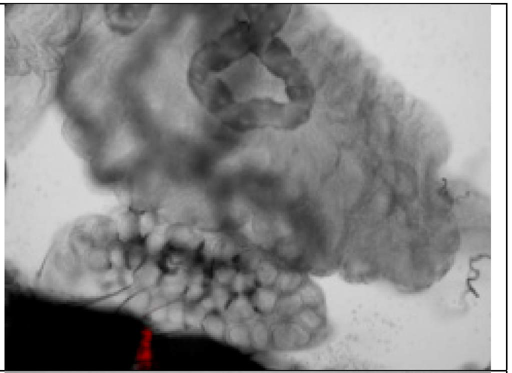
Ovary / auto-fluorescence in tracheole	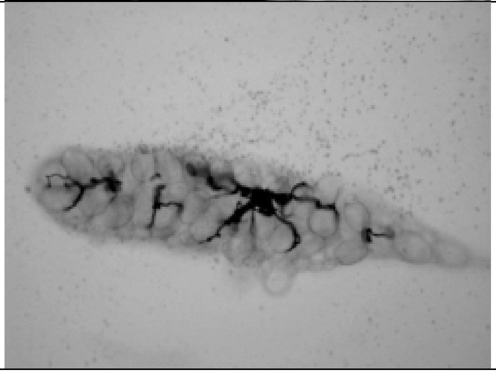		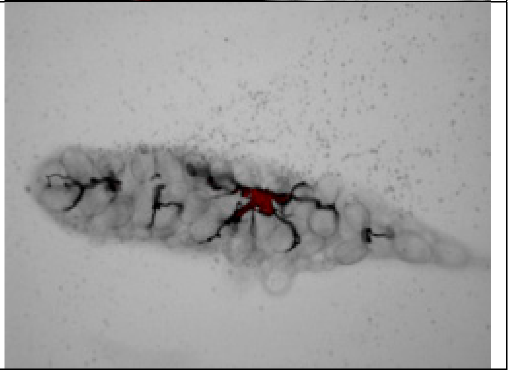

Figure S12 – Adult female *Cx. pipiens* – topical exposure

Tissue	Bright Field	RNAi trigger	Merge
Head / signal on cuticle and auto-fluorescence (reflected) in diverticula	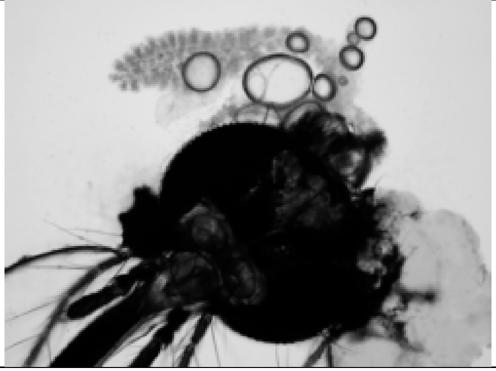	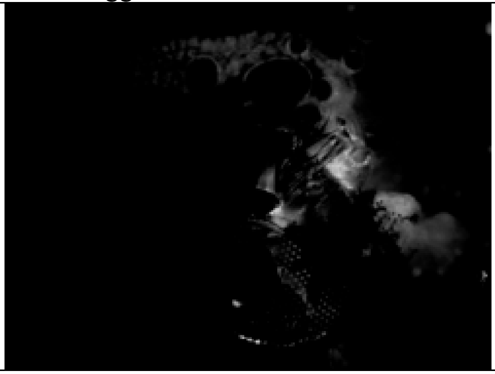	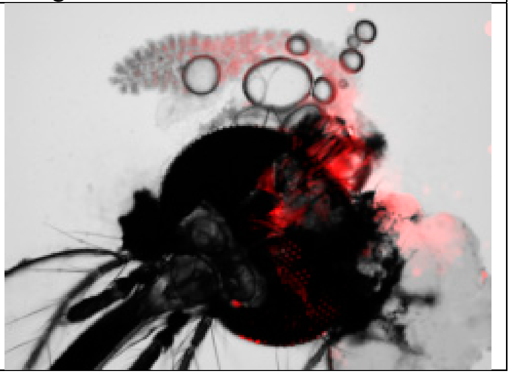

Head / signal on cuticle	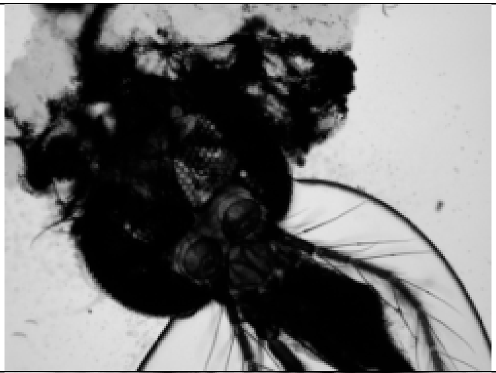	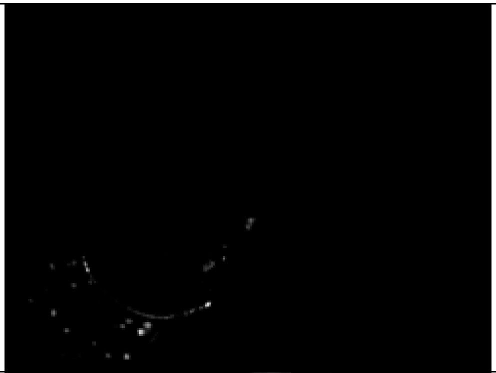	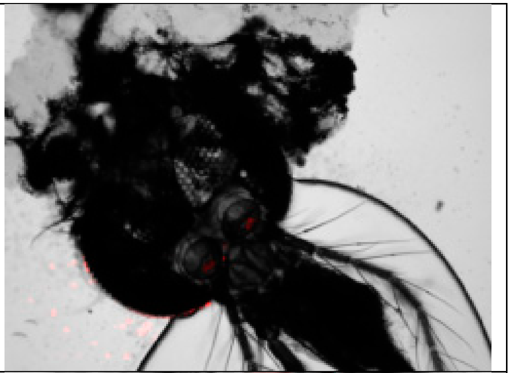
Thorax / signal on cuticle	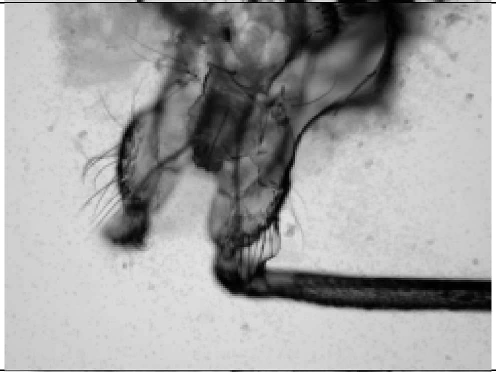	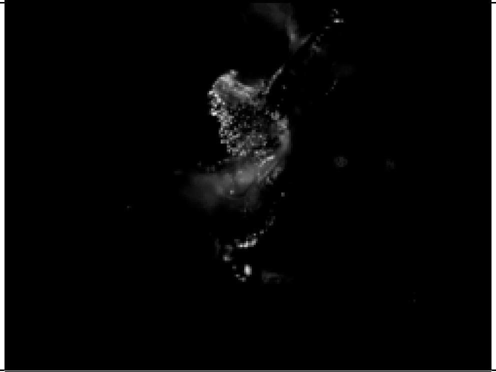	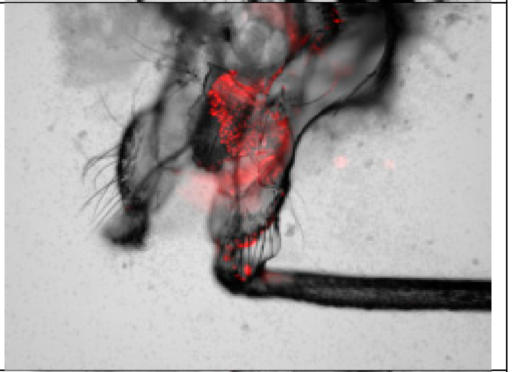
Abdomen /signal on cuticle	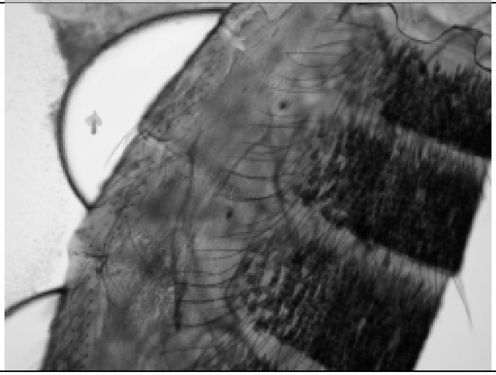	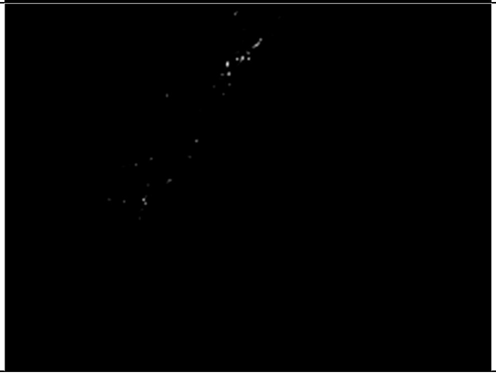	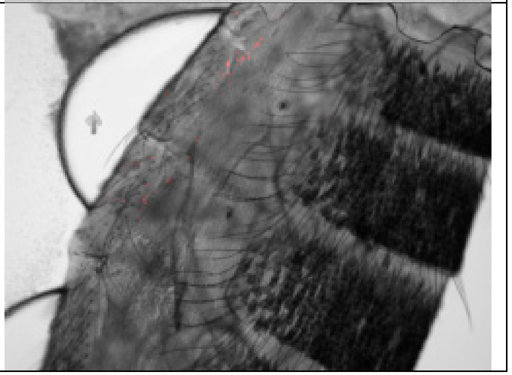

Abdomen / fat body no signal	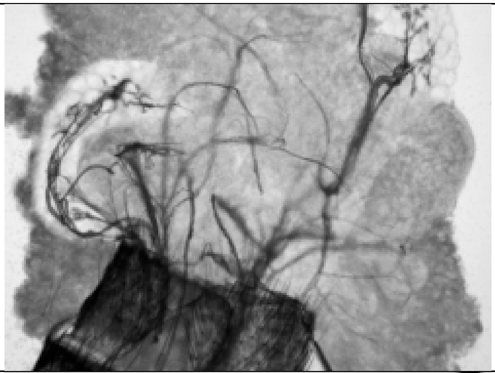	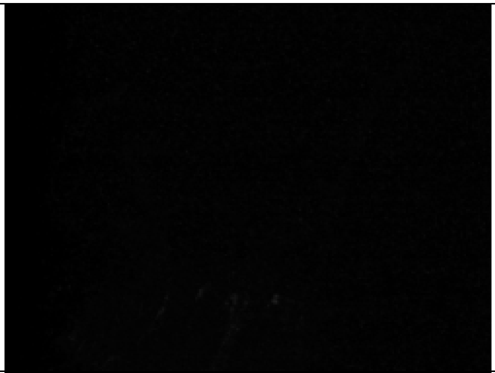	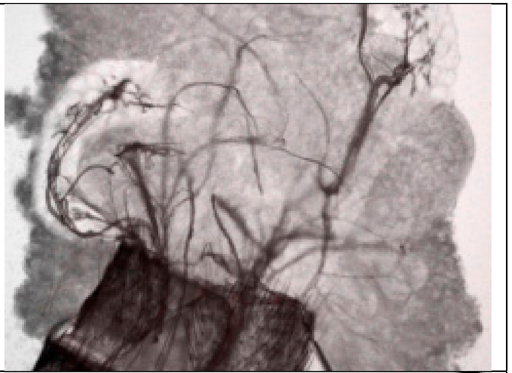
Leg / signal on cuticle	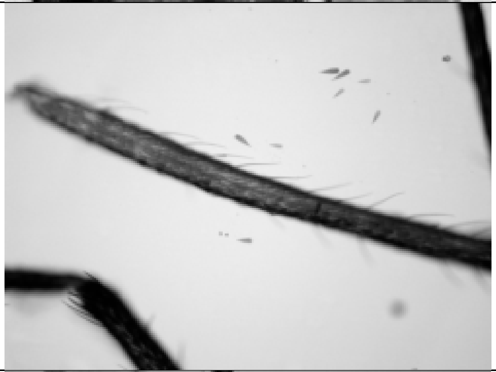	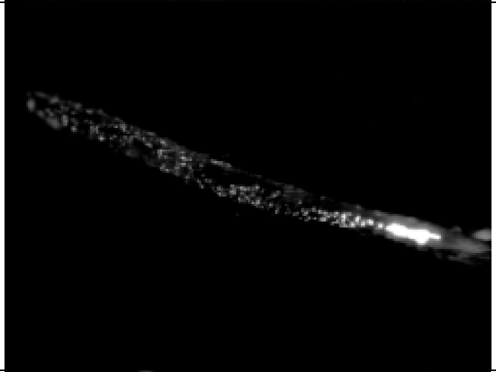	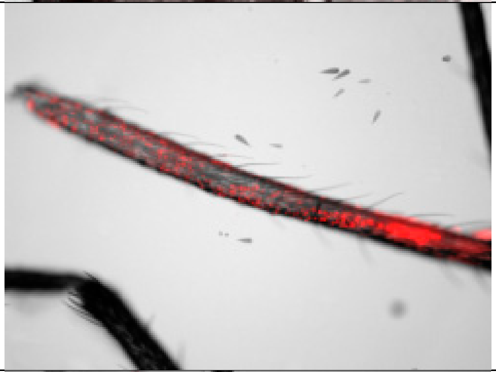
Alimentary tract / diverticula with auto-fluorescence	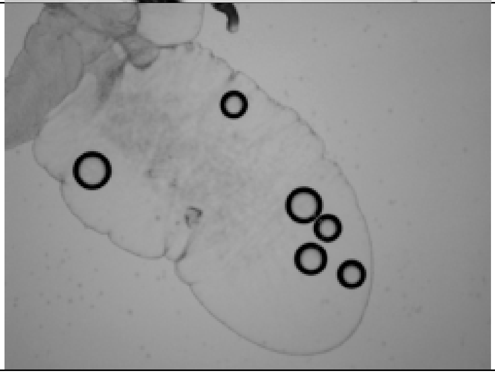	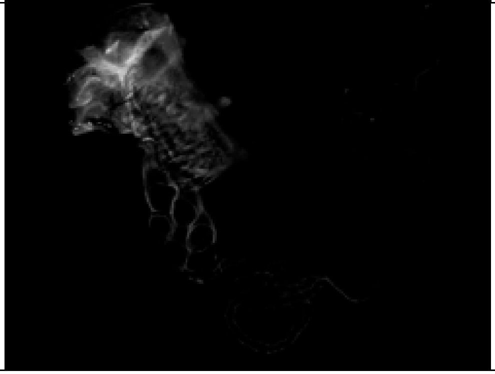	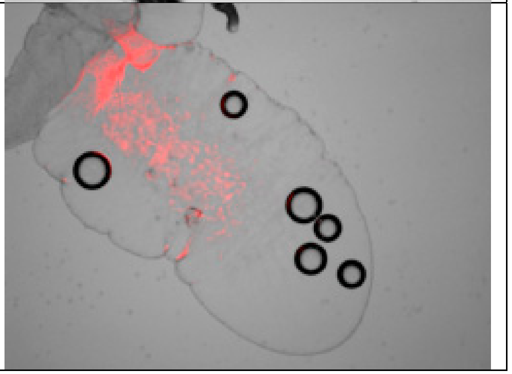

Alimentary tract (foregut, midgut and Malpighian tubules) with no signal	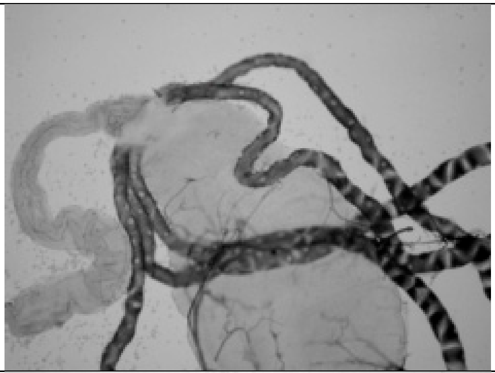	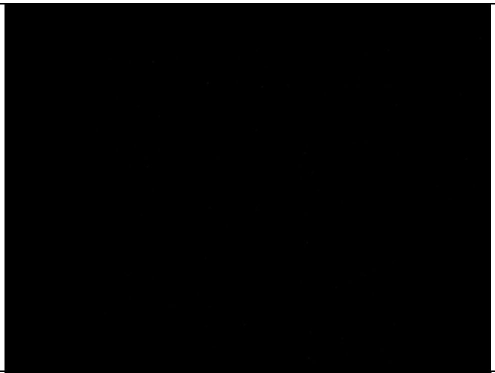	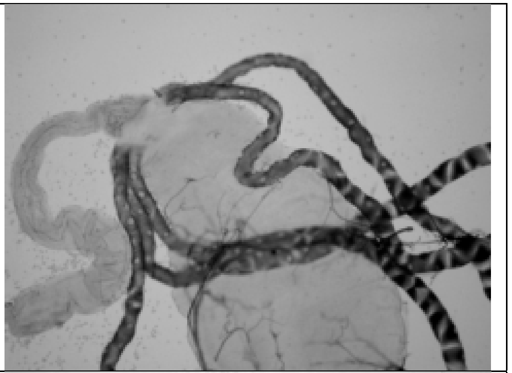
Alimentary tract (hindgut) and ovaries, fluorescent bubble in hindgut lumen probable auto-fluorescence	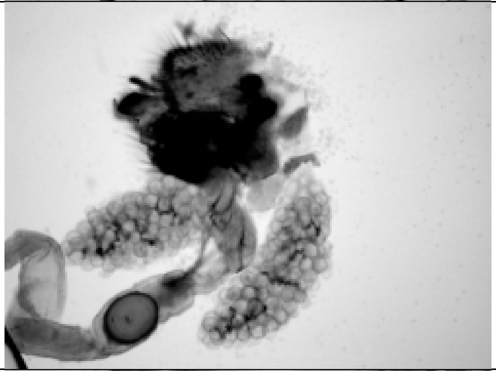	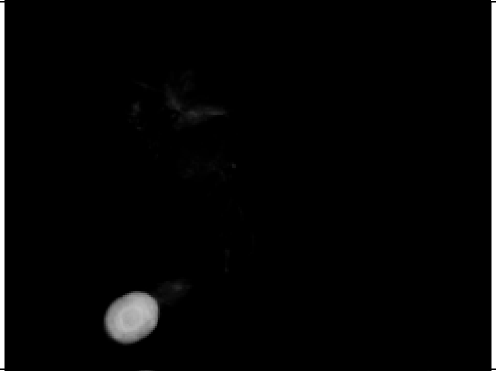	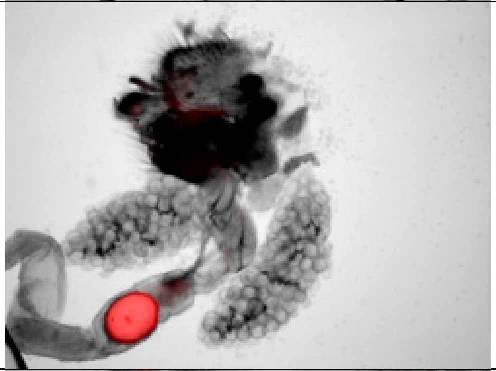
Abdomen / signal on cuticle and alimentary tract (hindgut, rectum, and Malpighian tubules) with auto-fluorescence (reflected)	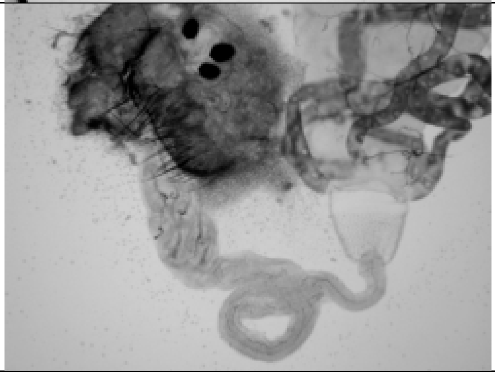	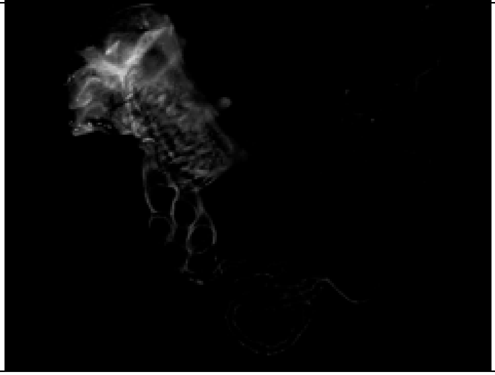	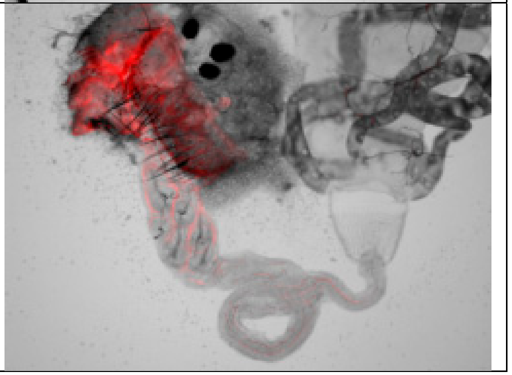

Adult exposure *Per os*

Figure S13 – Adult female *Ae. aegypti – per os*

Tissue	Bright Field	RNAi trigger	Merge
Head / signal at injection site / 24 HPE	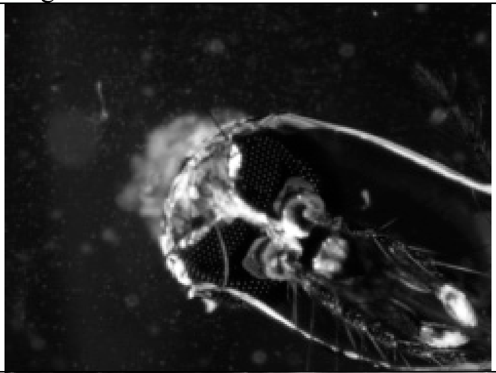	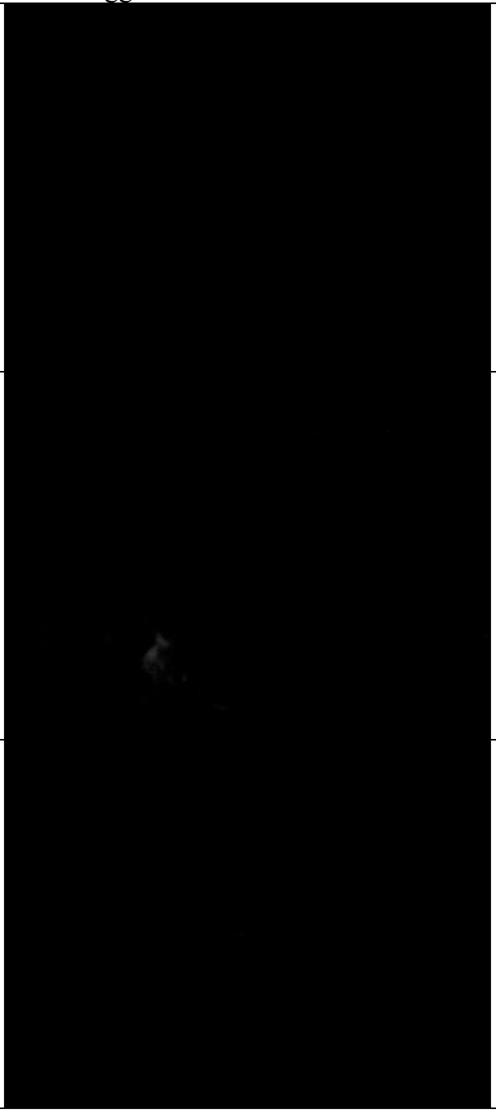	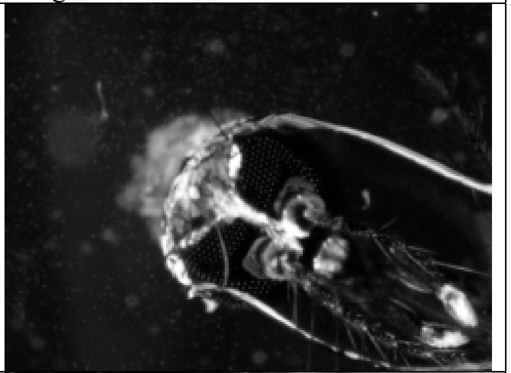
Thorax / auto-fluorescence / 72 HPE	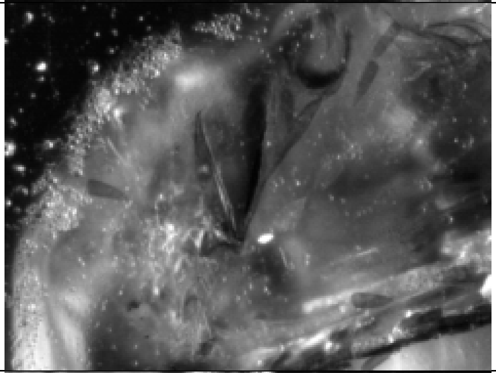		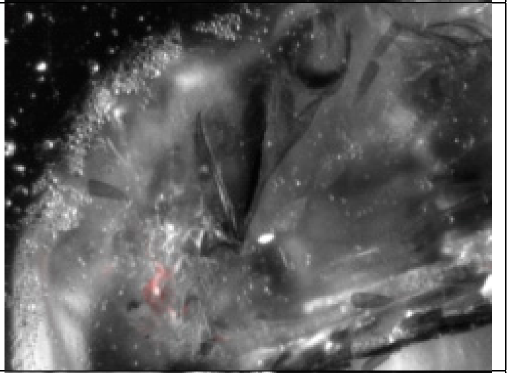
Thorax / no signal / 72 HPE	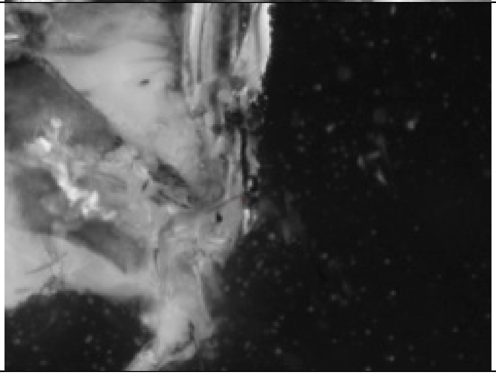		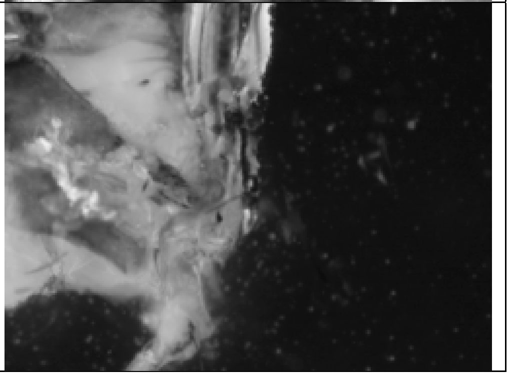

Abdomen / pericardial cells around dorsal vessel / 72 HPE	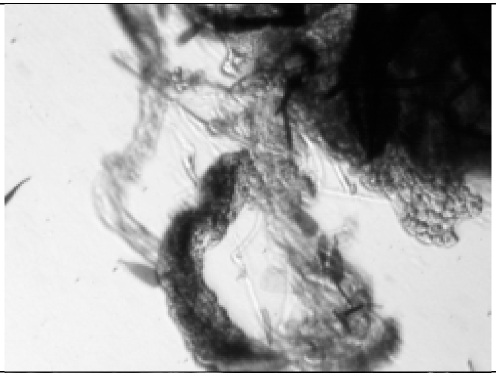	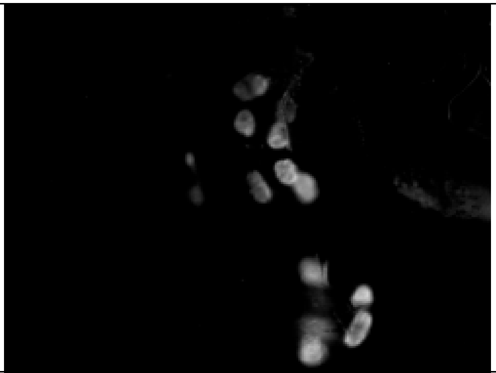	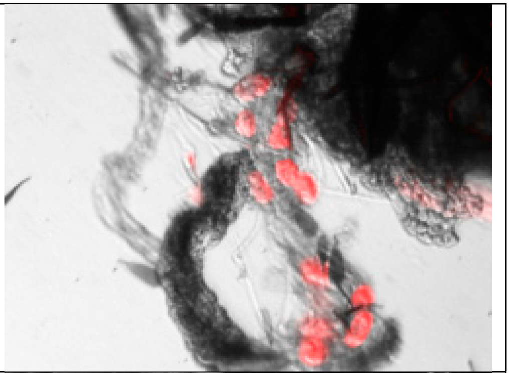
Abdomen / no signal / 120 HPE	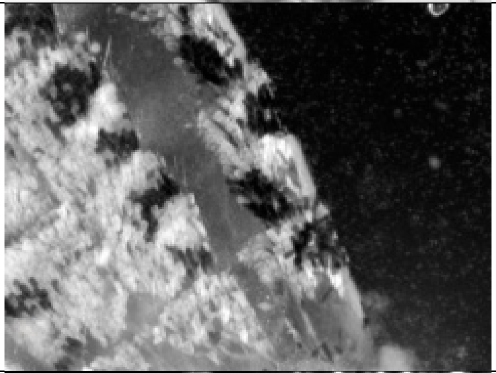	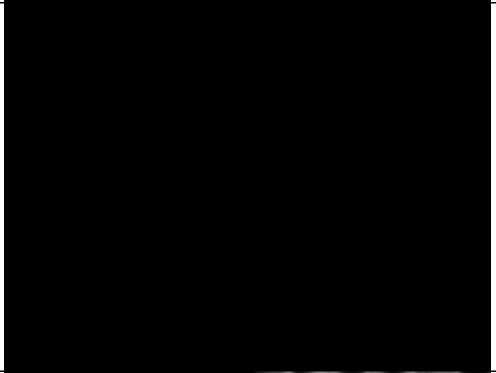	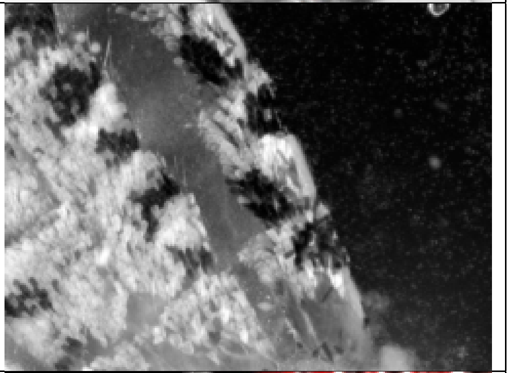
Diverticula with strong diffuse signal / 24 HPE	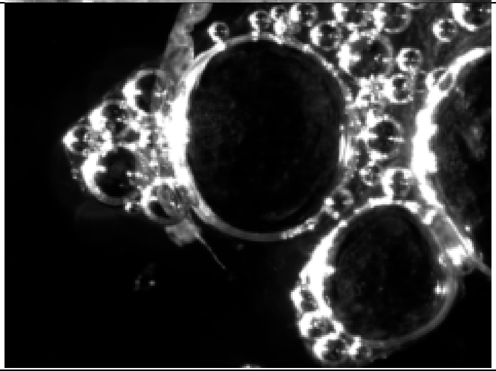	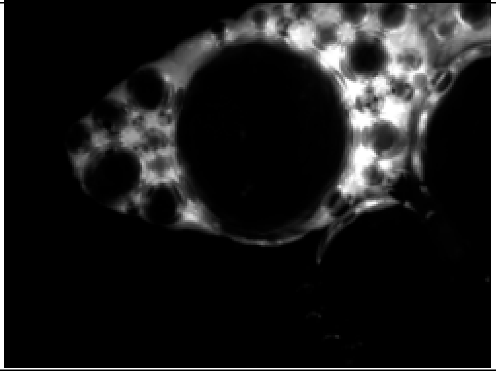	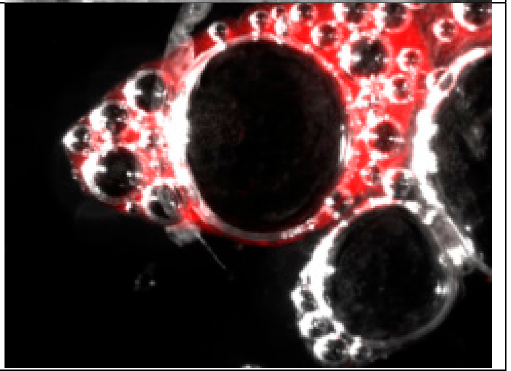

Diverticula with no signal / 120 HPE	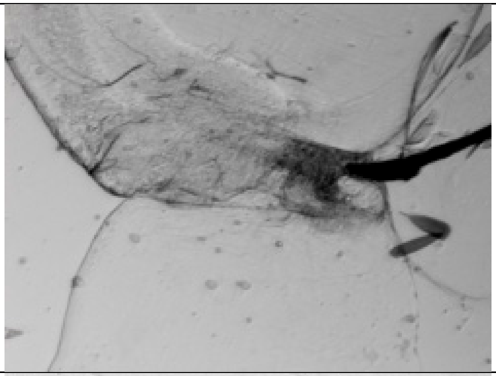	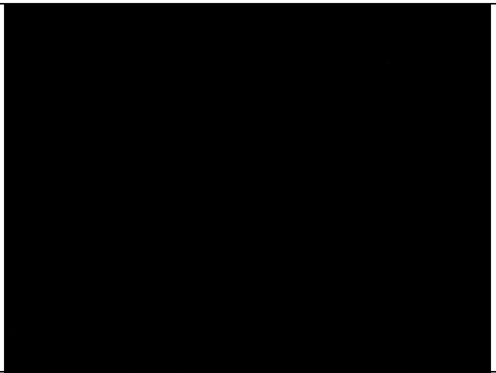	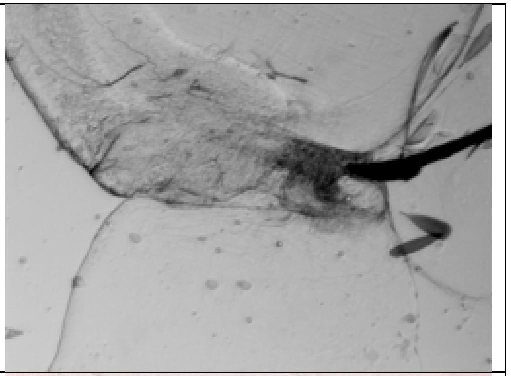
Alimentary tract / foregut with extracellular signal / 24 HPE	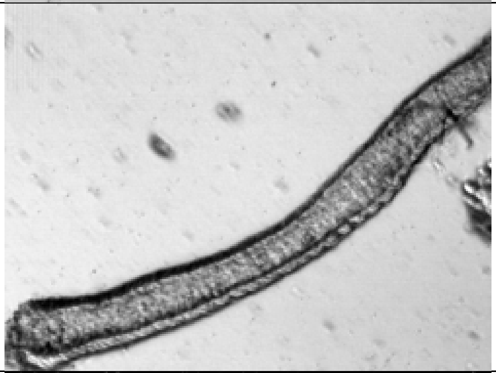	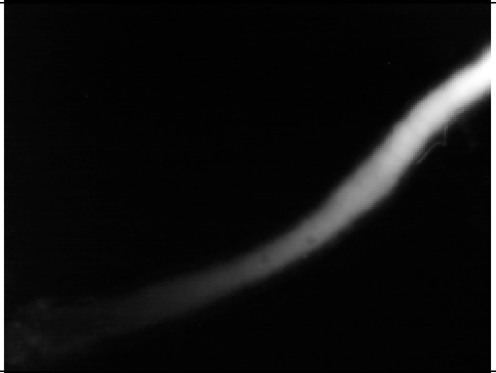	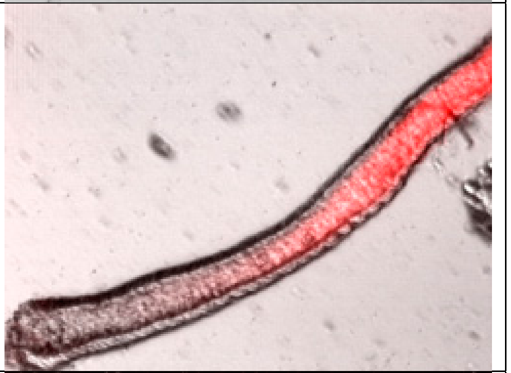
Alimentary tract (foregut, midgut, Malpighian tubules) with extracellular signal / 24 HPE	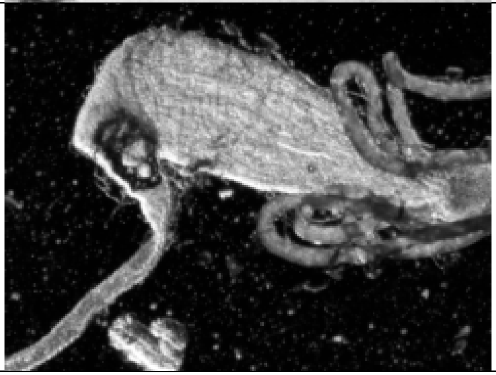	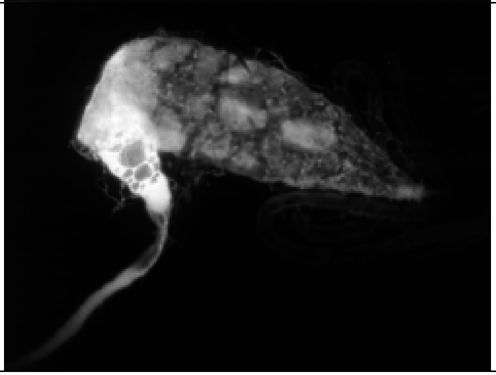	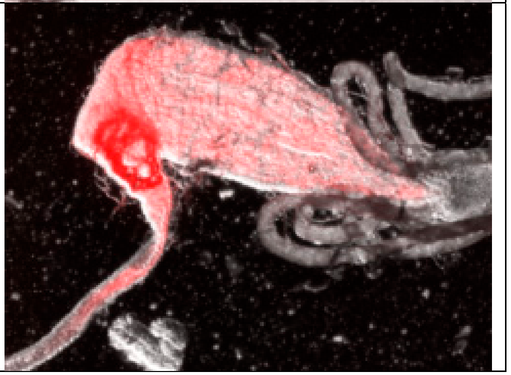

Alimentary tract (foregut, midgut, Malpighian tubules) no signal / 24 HPE	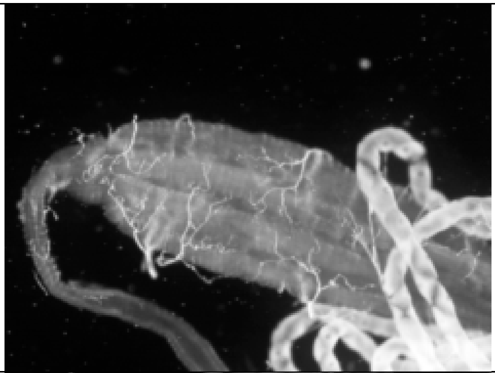	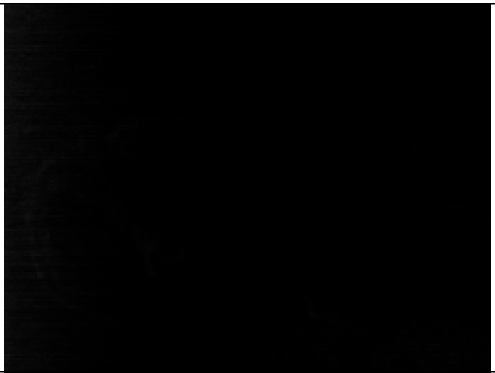	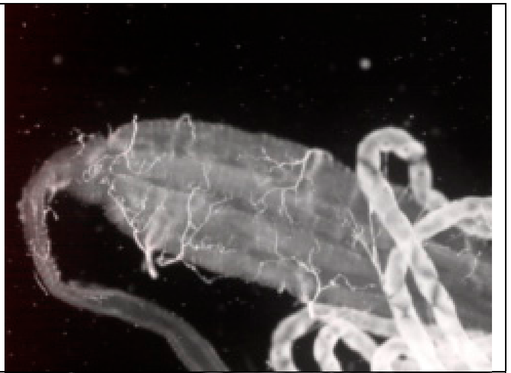
Alimentary tract / Malpighian tubules, no signal (72 HPE	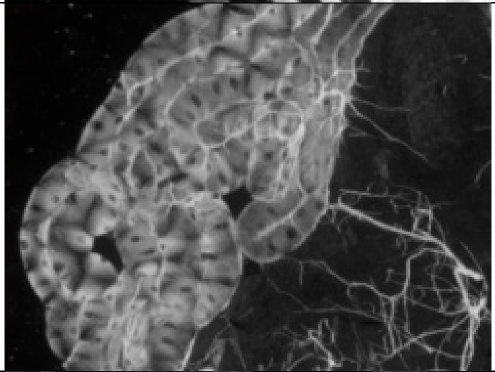	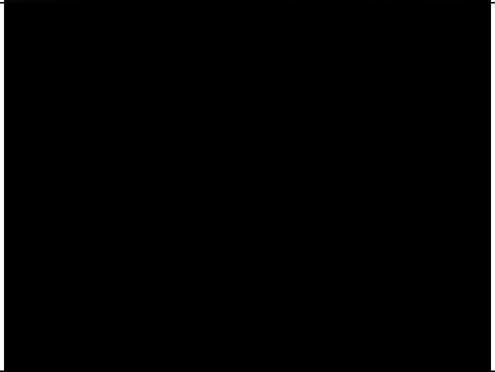	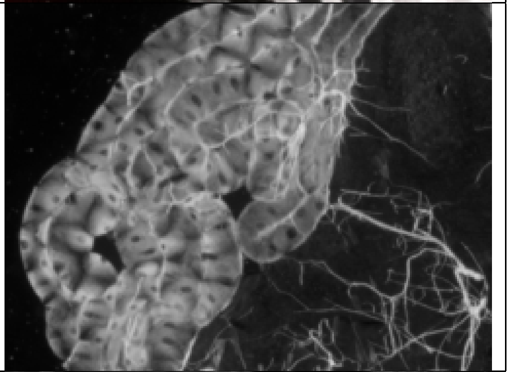
Alimentary tract / rectum, no signal / 120 HPE	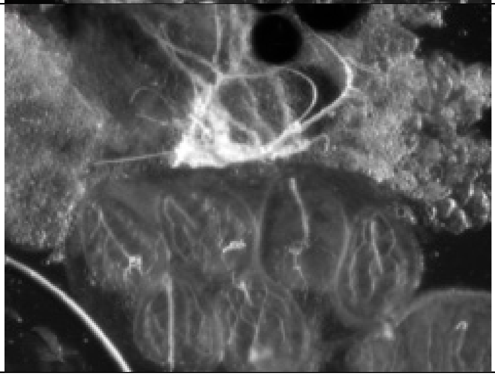	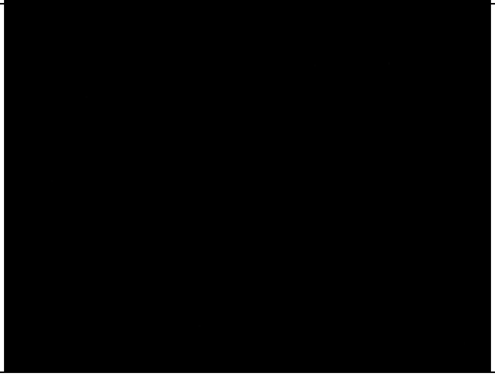	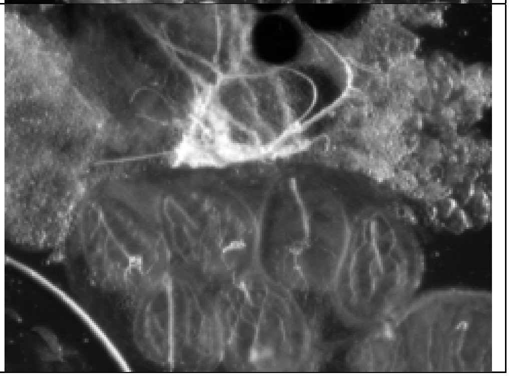

Ovary / no signal / 24 HPE	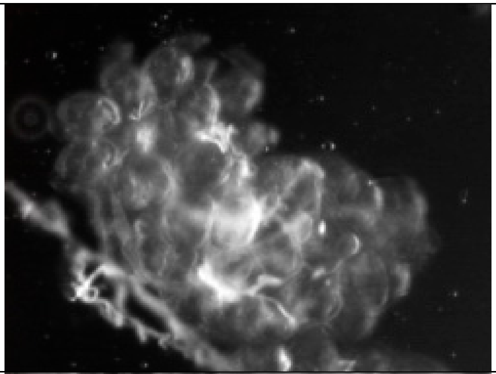	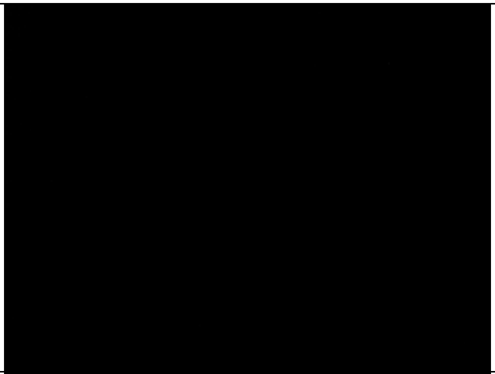	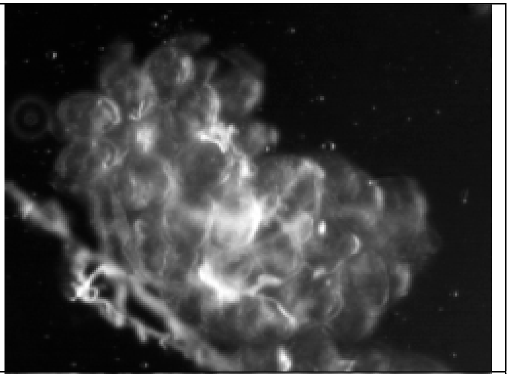
Ovary and Malpighian tubules / no signal / 72 HPE	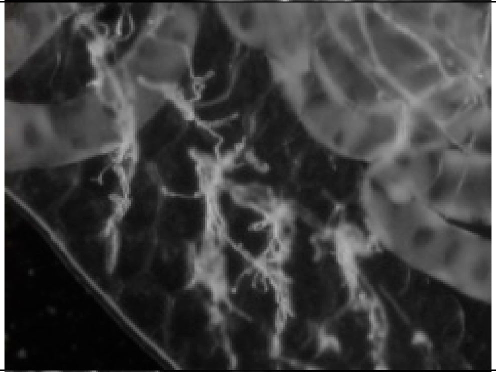	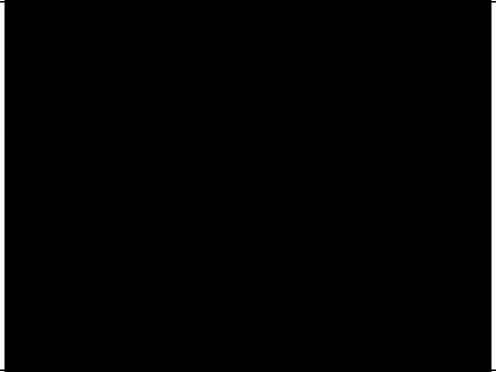	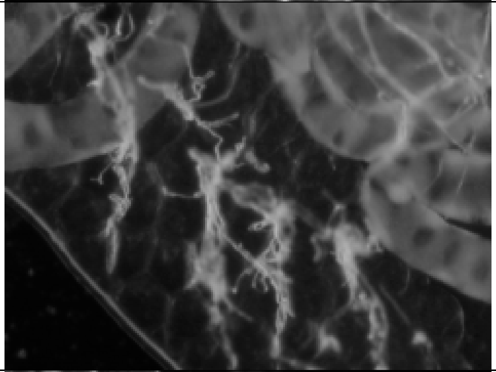
Ovary / no signal / 120 HPE	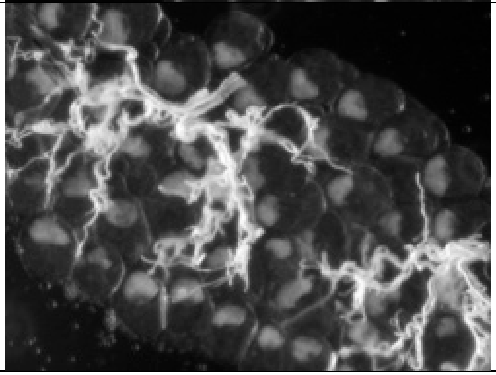	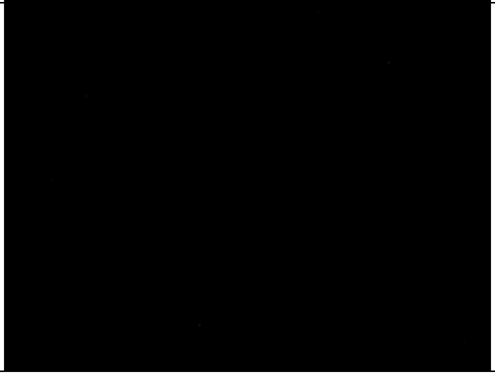	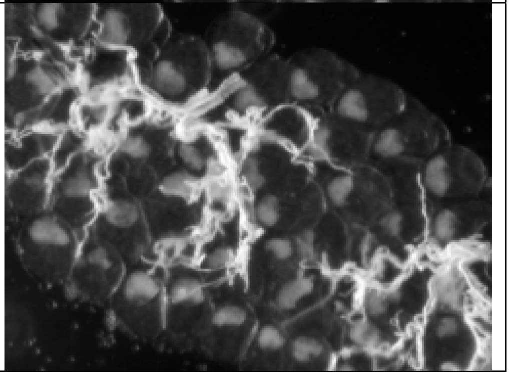

Figure S14 – Adult female *Cx. pipiens* – *per os*

Tissue	Bright Field	RNAi trigger	Merge
Head / signal at injection site / 24 HPE	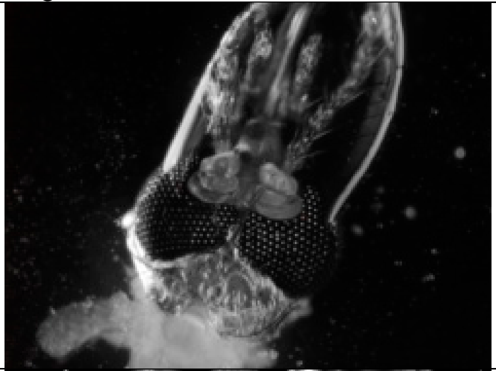	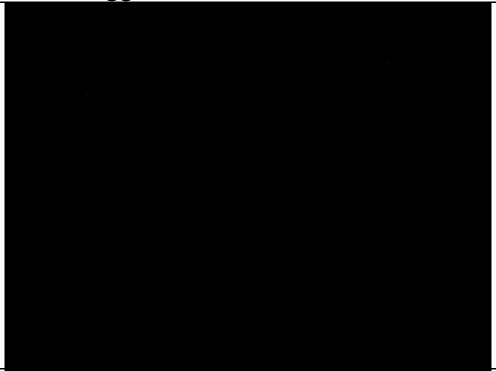	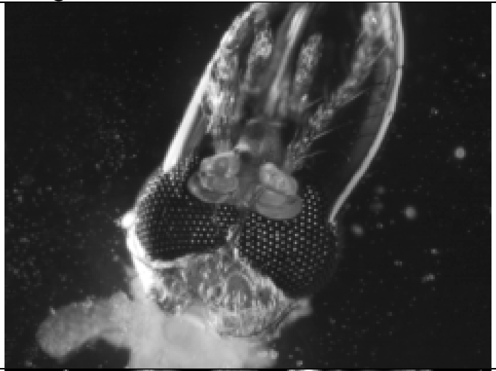
Head / hemocytes / 72 HPE	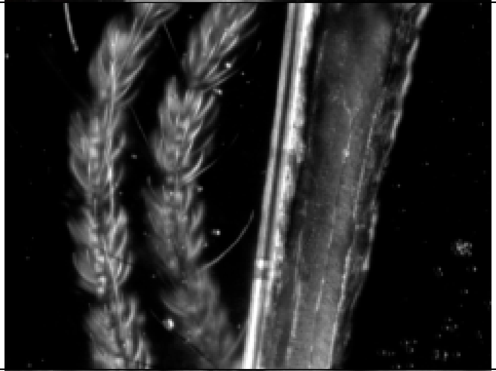	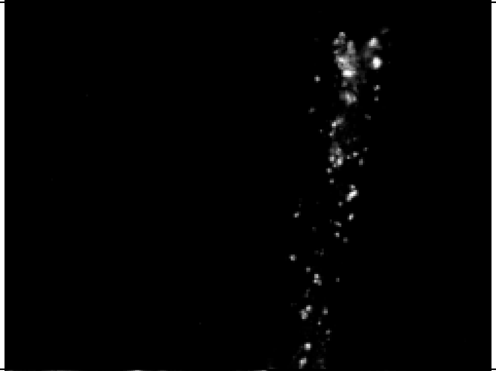	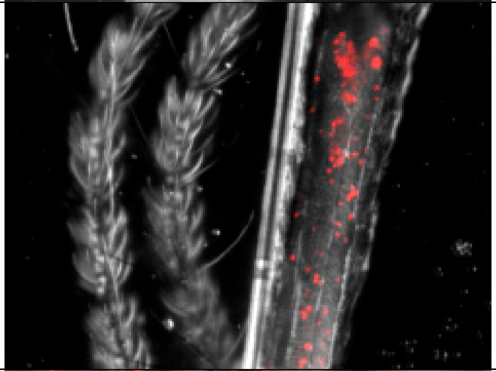
Thorax / hemocytes / 72 HPE	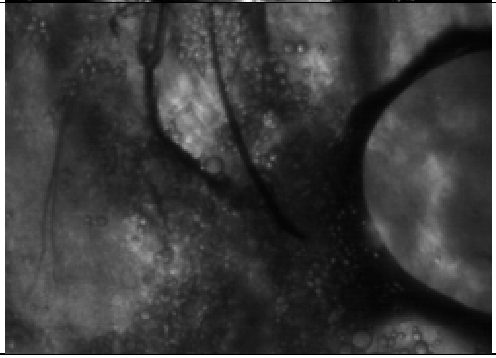	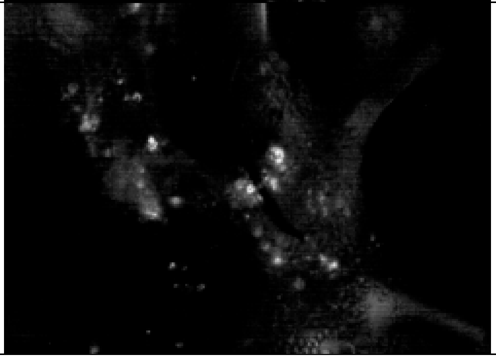	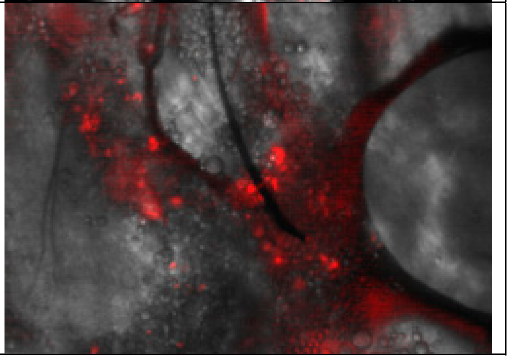

Thorax / hemocytes near muscle tissue / 72 HPE	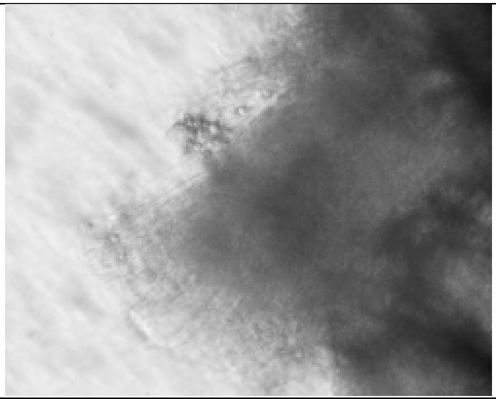	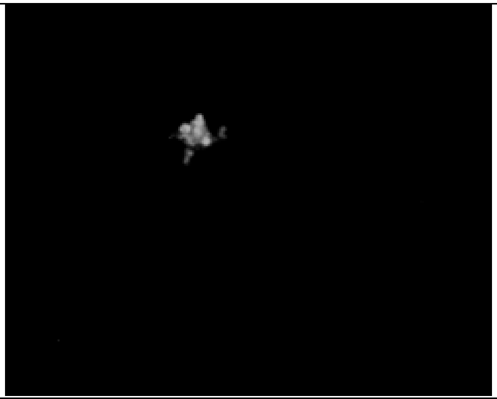	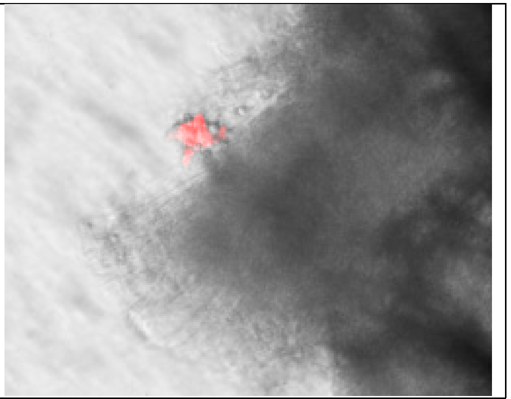
Abdomen / pericardial cells / 24 HPE	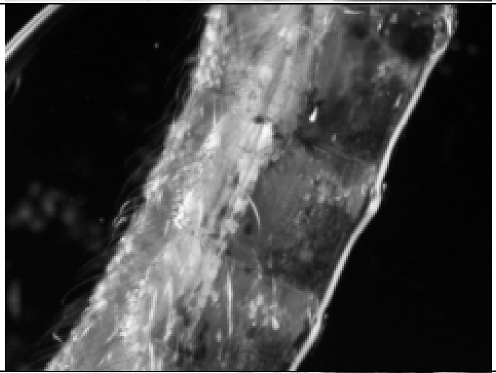	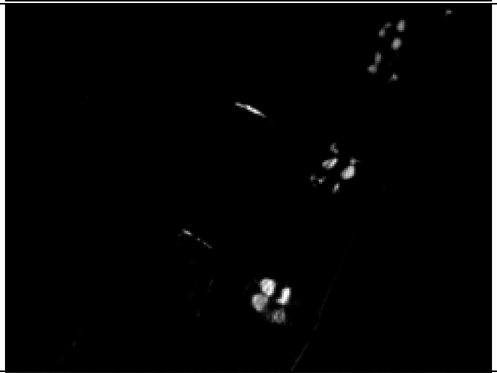	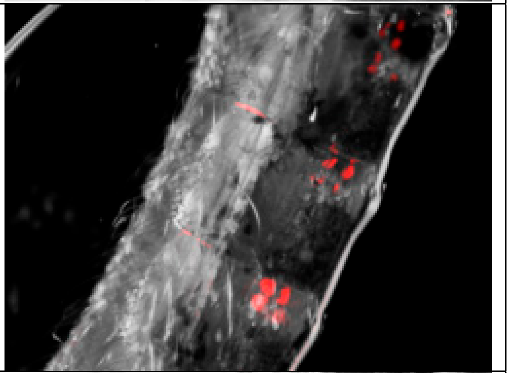
Abdomen / no signal / 120 HPE	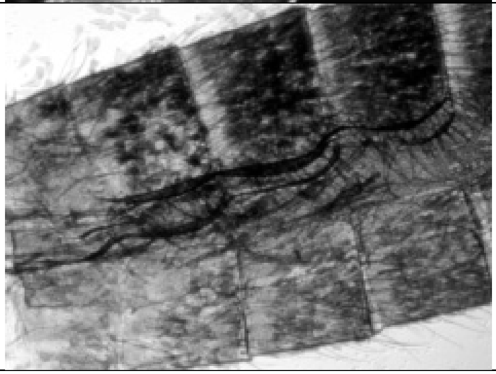	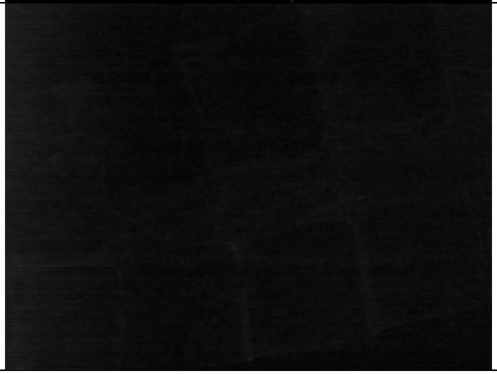	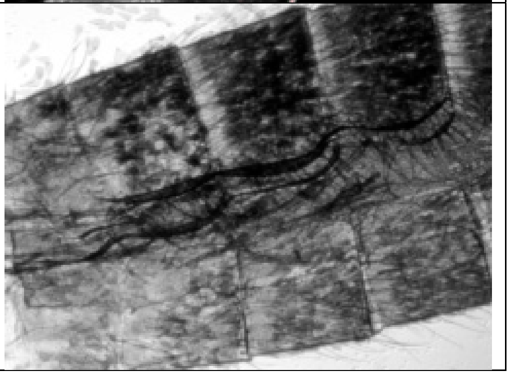

Malpighian tubules / hemocytes bound to tracheoles / 24 HPE	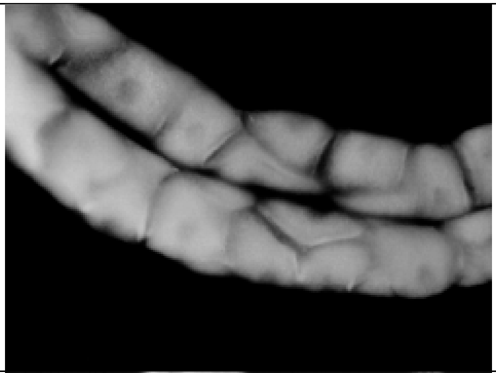	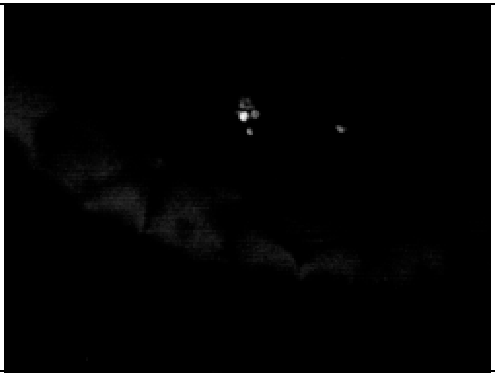	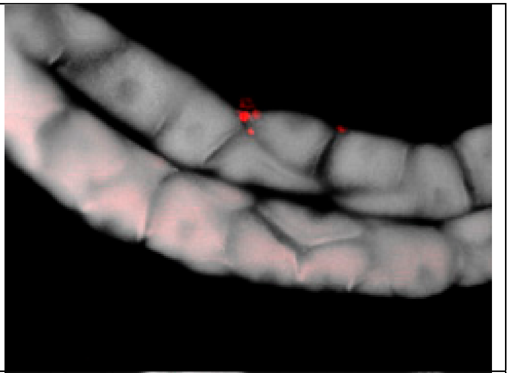
Leg / hemocytes / 72 HPE	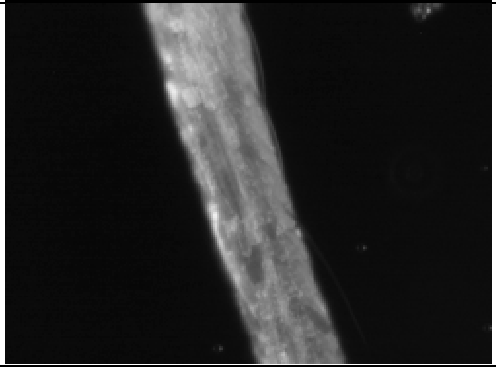	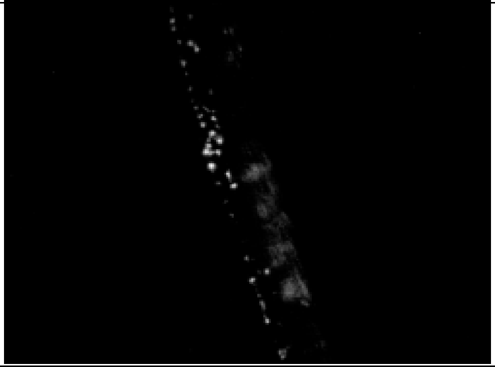	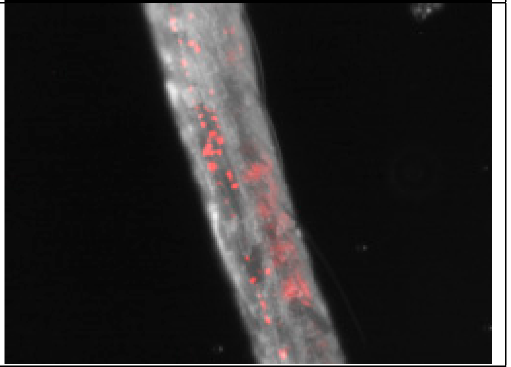
Diverticula with strong signal / 24 HPE	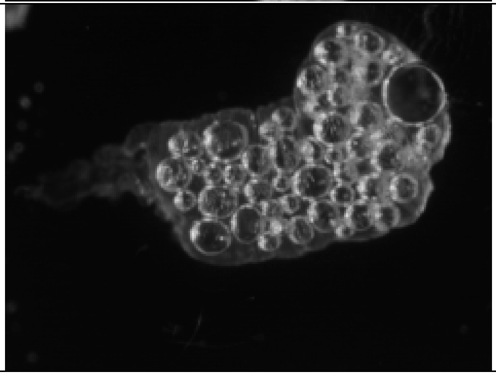	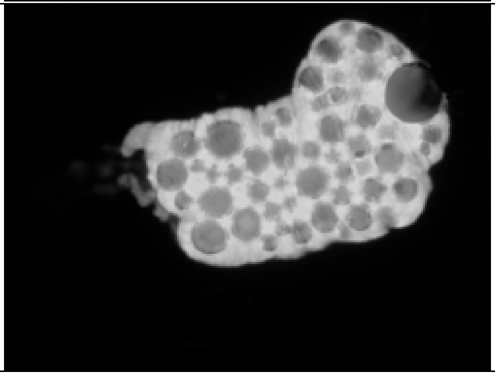	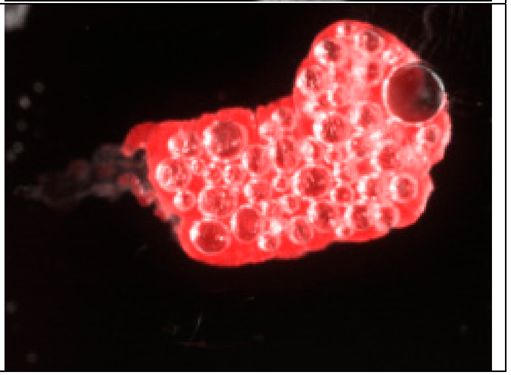

Foregut / punctate staining in tissue / 72 HPE	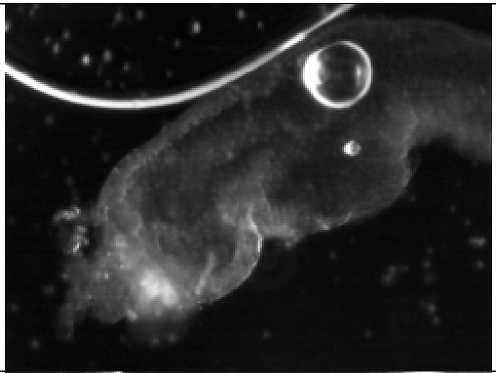	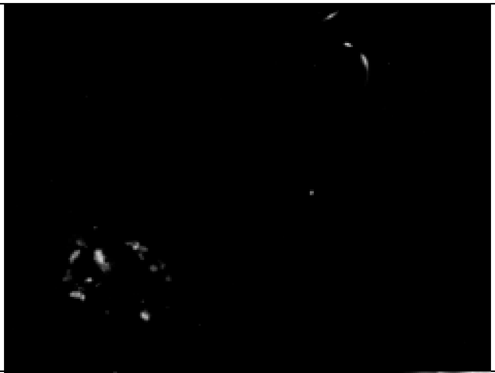	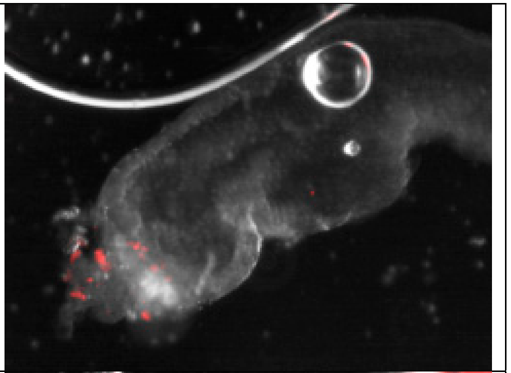
Foregut lumen / 72 HPE	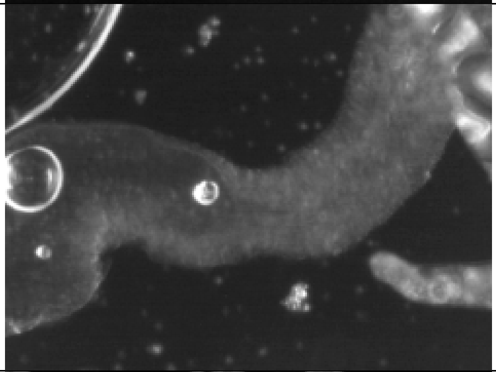	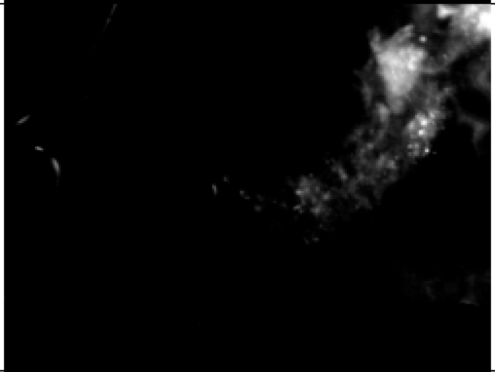	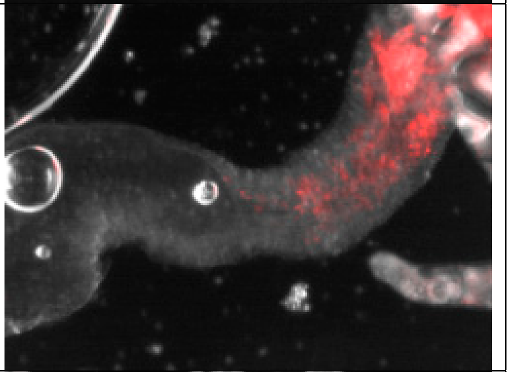
Alimentary tract with strong midgut / 24 HPE	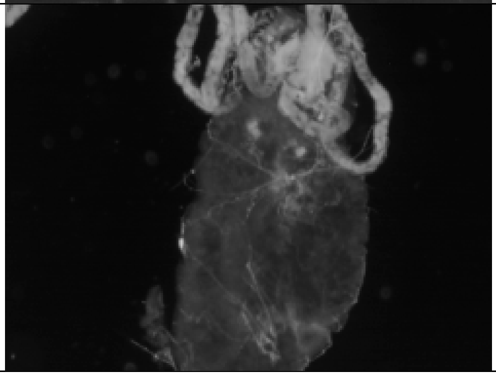	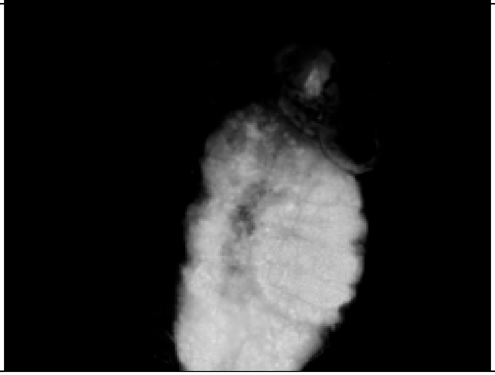	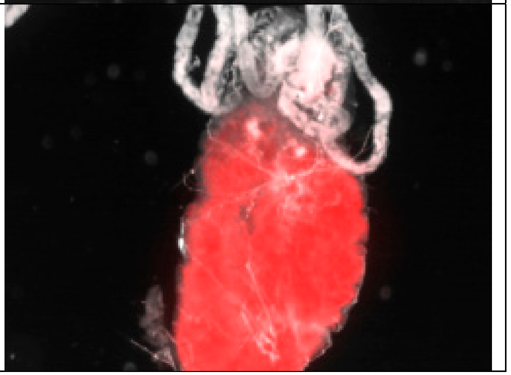

Alimentary tract with strong midgut and foregut / 72 HPE	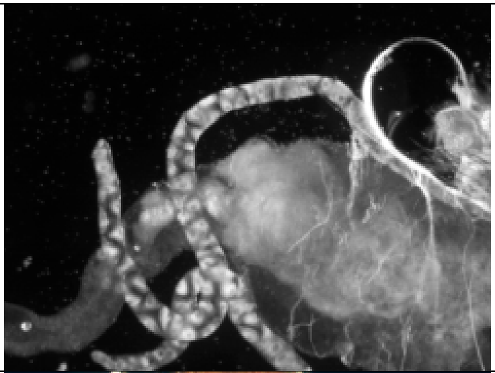	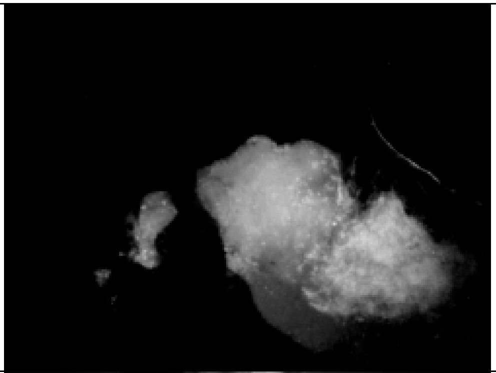	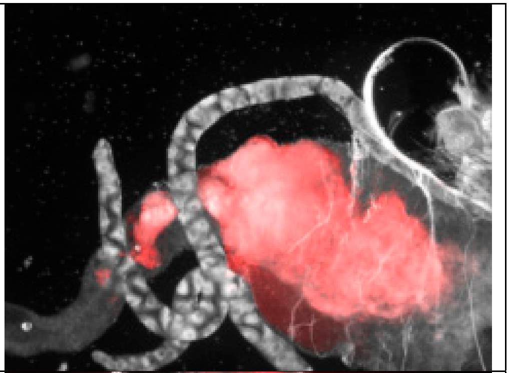
Hindgut lumen and ovaries / 24 HPE * a color version of this image was included to demonstrate visible Cy3	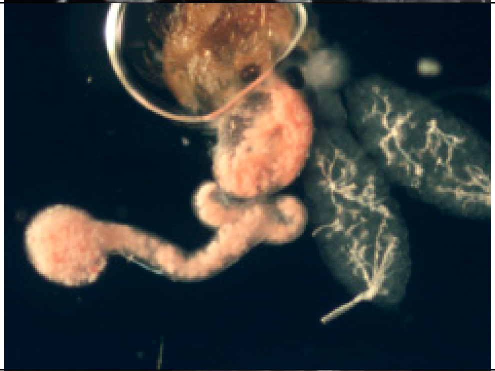	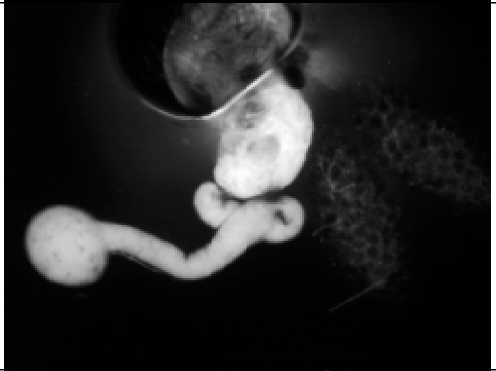	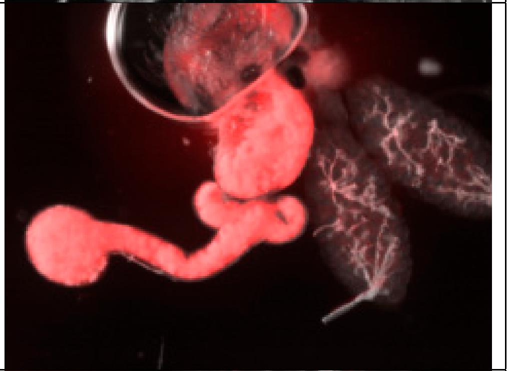
Ovary / hemocytes / 72 HPE	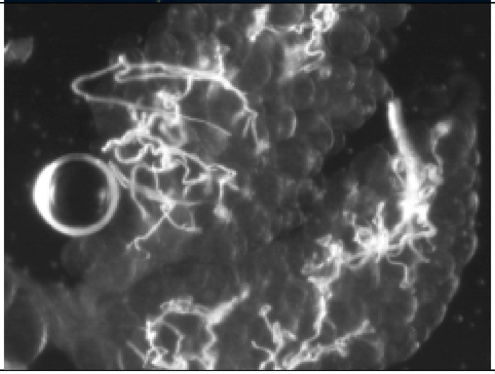	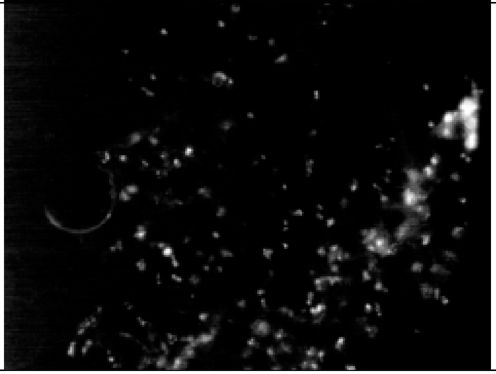	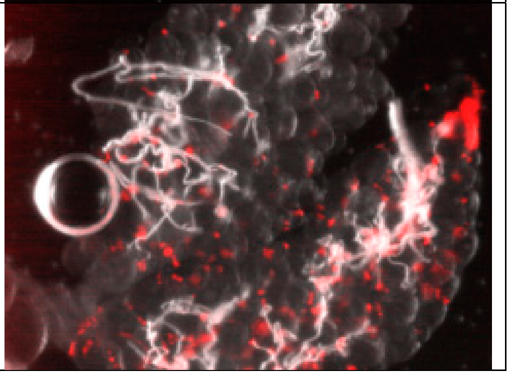

Ovary / no signal / 72 HPE	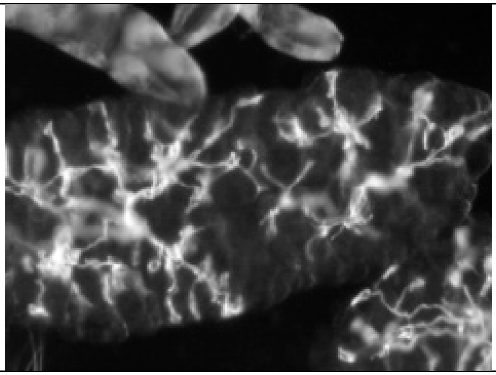	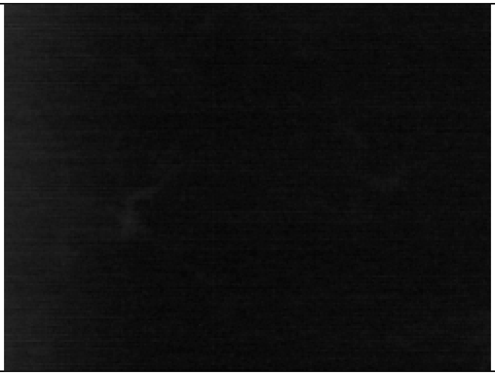	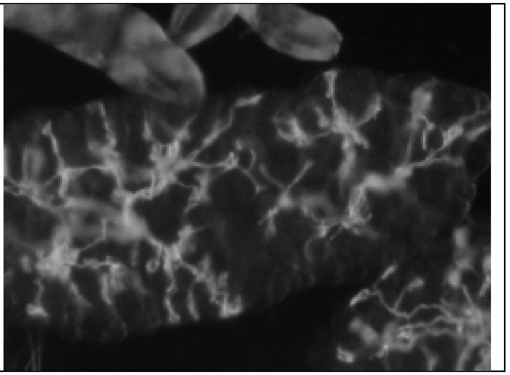

Larval exposure by Soaking

Figure S15 – 1st instar *Ae. aegypti* - soaking

Tissue	Bright Field	RNAi trigger	Merge
Head, thorax and abdomen / small punctate intestinal tract / 24 HPE	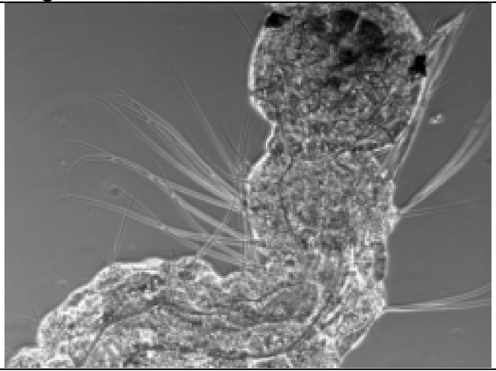	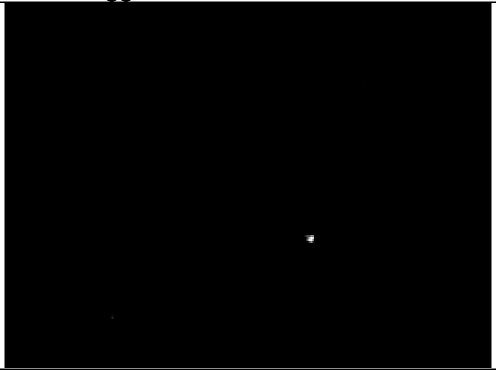	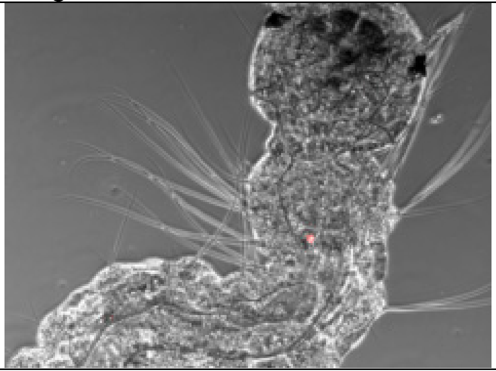

Head and thorax / caecum with signal / 24 HPE	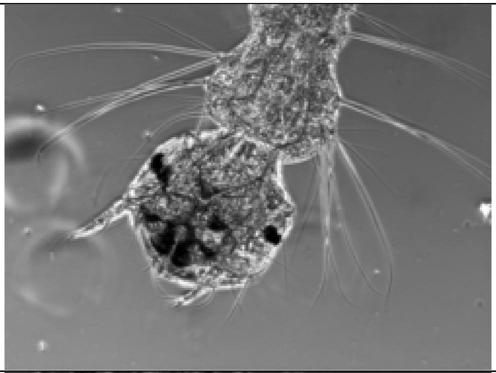	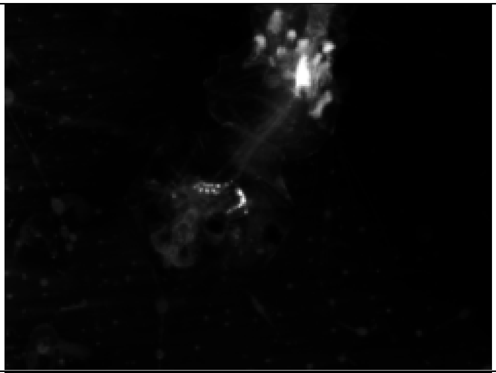	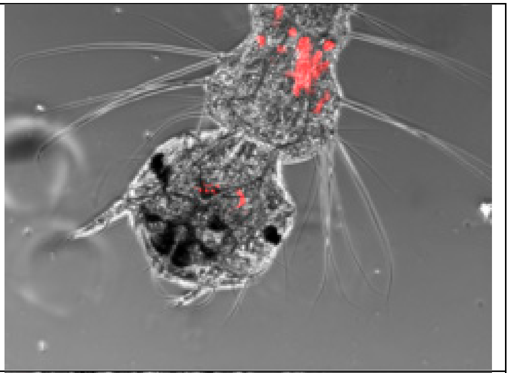
Abdomen with intestinal lumen / 24 HPE * Dark spot indicates yeast feed which was not auto-fluorescent	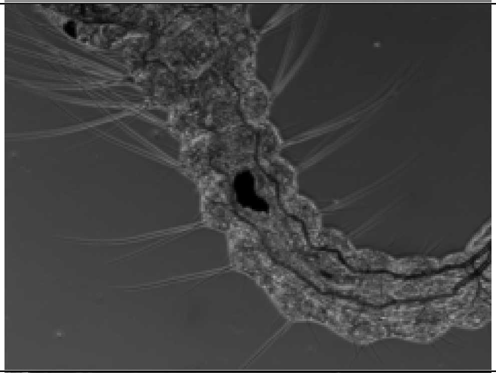	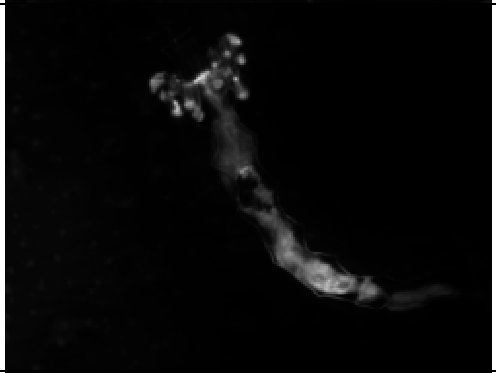	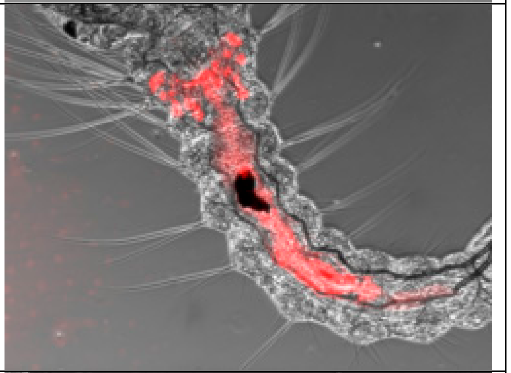
Abdomen with intestinal lumen and excreted from rectum / 24 HPE	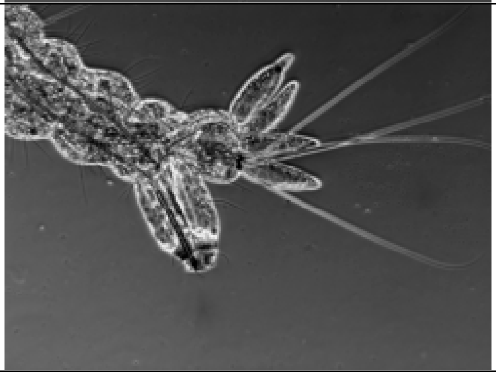	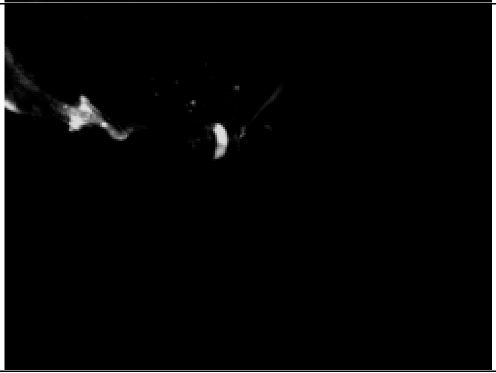	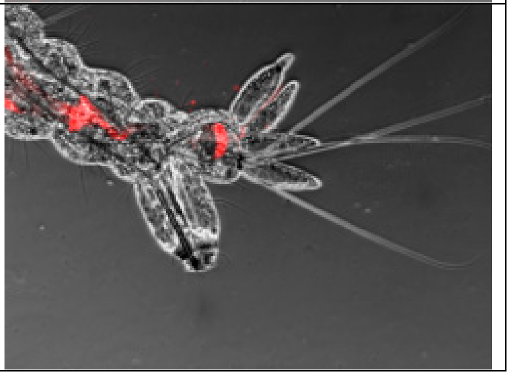

Head, thorax, and abdomen with intestinal lumen and head / 72 HPE	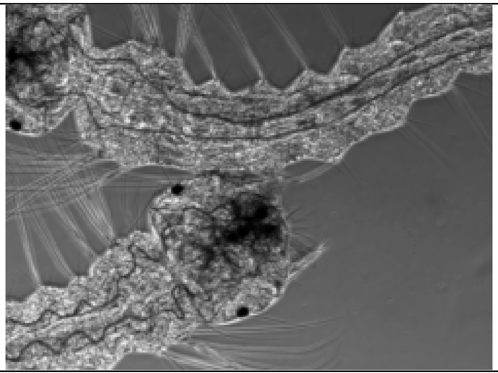	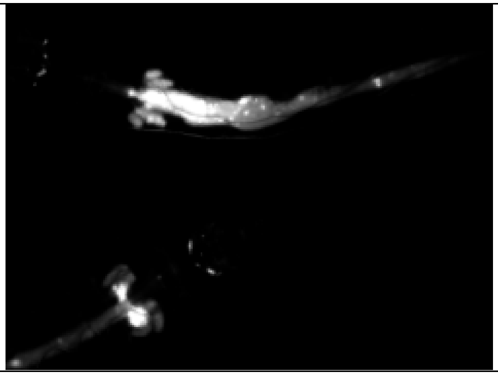	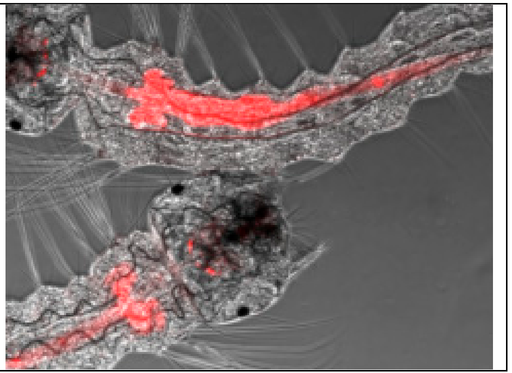

Figure S16 – Stability of iLacZ in sucrose meal following exposure to *Ae. aegypti* in insectary conditions.
iLacZ does not degrade following exposure to mosquitoes in sucrose solutions. Sucrose solutions containing 1 mg/ml iLacZ were incubated for 12 hours in insectary conditions in a: closed micro/centrifuge tube (closed), open micro/centrifuge tube lid (open), or an open micro/centrifuge tube lid in a carton of 20 starved adult female *Ae. aegypti* (open + *Ae. aegypti*). Integrity of iLacZ was measured by band intensity following gel electrophoresis compared to frozen iLacZ stock. No significant differences were detected between groups as measured by one way ANOVA.

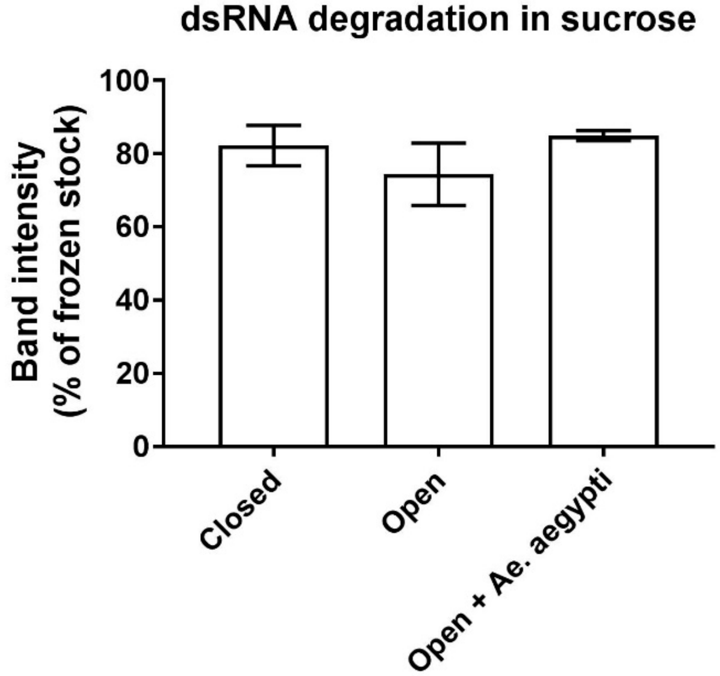

Figure S17 – Confocal Z/stack of *Ae. aegypti* ovary 24 hours post peritoneal exposure of fluorescent/iLacZ.
iLacZ uptake assessed by confocal microscopy in non/bloodfed *Ae. aegypti* ovaries. Cross section stack (Z/stack) of *Ae. aegypti* ovaries 24 hours post injection with labelled iLacZ showing DAPI stained nuclei (blue) and Cy5 labelled iLacZ (magenta).

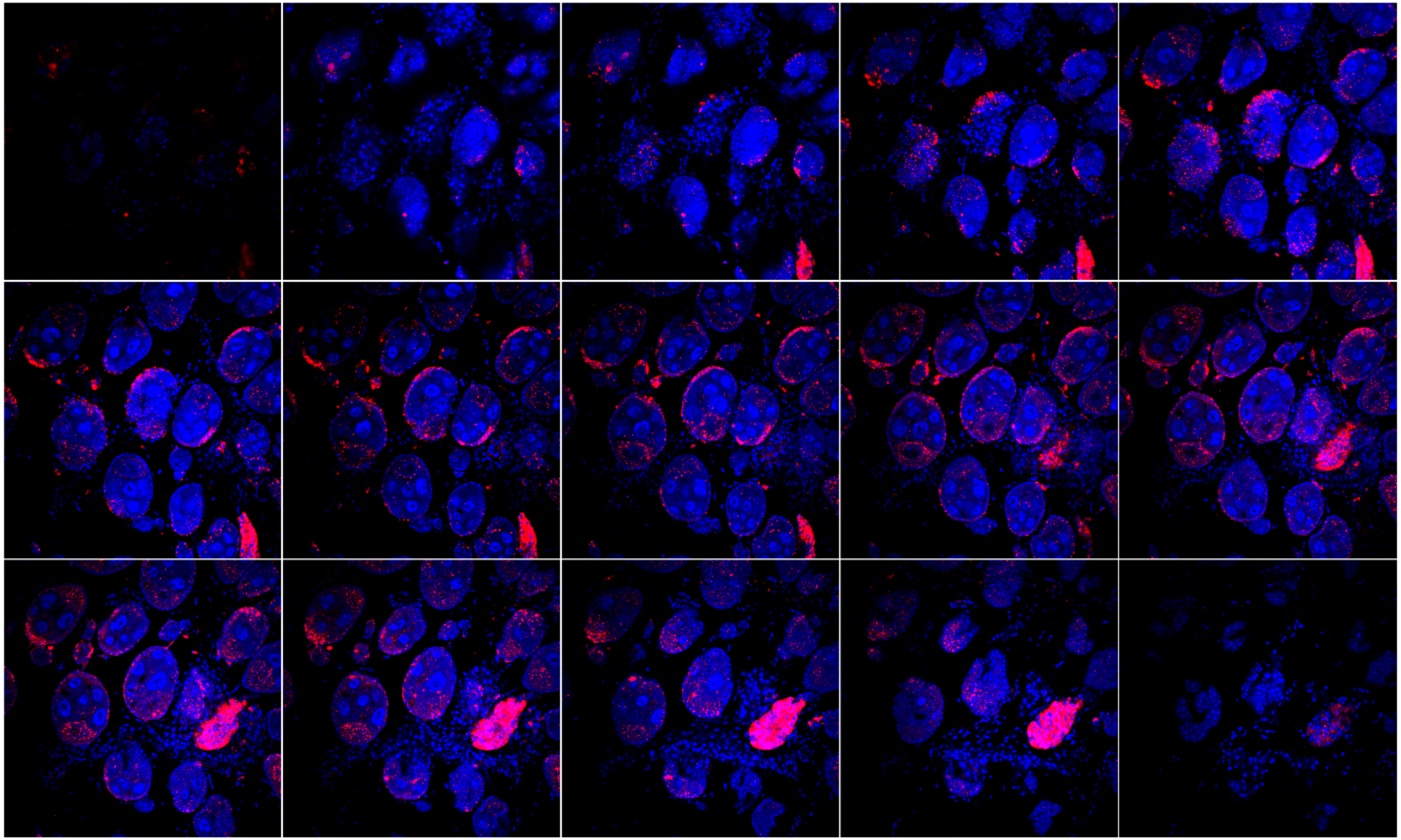

Figure S18 – Northern blot time-course of C6/36 cells following iLacZ exposure.

Persistence of a 377 base-pair iLacZ tracked by Northern blot following exposure to *Ae. albopictus* C6/36 cells. Cell extracts (1 µg) were tested at 0.25, 1, 24, and 72 hours post exposure against a 400 bp iLacZ RNA probe (iL). Ribosomal RNA loading controls (LC) are shown from *Ae. albopictus* RNA extracts imaged immediately prior to Northern membrane transfer.

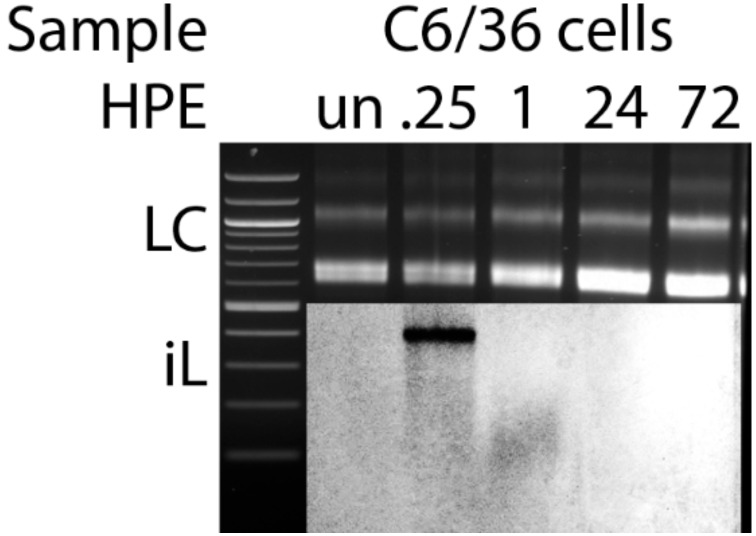

Supplement: Supplementary file 1 [file insects-14-00556-s001.zip › insects-2403396-supplementary.pdf]
